# Notes from the taxonomic disaster zone: Evolutionary drivers of intractable species boundaries in an Australian lizard clade (Scincidae: *Ctenotus*)

**DOI:** 10.1111/mec.17074

**Published:** 2023-07-17

**Authors:** Ivan Prates, Mark N. Hutchinson, Sonal Singhal, Craig Moritz, Daniel L. Rabosky

**Affiliations:** ^1^ Department of Ecology and Evolutionary Biology and Museum of Zoology University of Michigan Ann Arbor Michigan USA; ^2^ South Australia Museum Adelaide South Australia Australia; ^3^ Department of Biology California State University — Dominguez Hills Carson California USA; ^4^ Division of Ecology and Evolution and Centre for Biodiversity Analysis Australian National University Canberra Australian Capital Territory Australia

**Keywords:** cryptic species, hybridization, introgression, isolation‐by‐distance, speciation, species delimitation

## Abstract

Genomic‐scale datasets, sophisticated analytical techniques, and conceptual advances have disproportionately failed to resolve species boundaries in some groups relative to others. To understand the processes that underlie taxonomic intractability, we dissect the speciation history of an Australian lizard clade that arguably represents a “worst‐case” scenario for species delimitation within vertebrates: the *Ctenotus inornatus* species group, a clade beset with decoupled genetic and phenotypic breaks, uncertain geographic ranges, and parallelism in purportedly diagnostic morphological characters. We sampled hundreds of localities to generate a genomic perspective on population divergence, structure, and admixture. Our results revealed rampant paraphyly of nominate taxa in the group, with lineages that are either morphologically cryptic or polytypic. Isolation‐by‐distance patterns reflect spatially continuous differentiation among certain pairs of putative species, yet genetic and geographic distances are decoupled in other pairs. Comparisons of mitochondrial and nuclear gene trees, tests of nuclear introgression, and historical demographic modelling identified gene flow between divergent candidate species. Levels of admixture are decoupled from phylogenetic relatedness; gene flow is often higher between sympatric species than between parapatric populations of the same species. Such idiosyncratic patterns of introgression contribute to species boundaries that are fuzzy while also varying in fuzziness. Our results suggest that “taxonomic disaster zones” like the *C. inornatus* species group result from spatial variation in the porosity of species boundaries and the resulting patterns of genetic and phenotypic variation. This study raises questions about the origin and persistence of hybridizing species and highlights the unique insights provided by taxa that have long eluded straightforward taxonomic categorization.

## 1 INTRODUCTION

Species delimitation has long relied on the phenotypic attributes of organisms under the premise that character differences reflect evolutionary divergence (de Queiroz, 1998, 2007; Hennig, 1966). Molecular approaches revolutionized taxonomy by revealing that species boundaries inferred from morphological characters can be inconsistent with patterns of evolutionary divergence, sometimes supporting the refinement of morphological diagnoses (Prates, Hutchinson, et al., 2022; Sites & Marshall, 2004; Teixeira et al., 2016). Observations of uncoupled genetic and morphological differentiation have now become ubiquitous, as illustrated by increasing reports of polymorphic and cryptic species (Vacher et al., 2020; Veijalainen et al., 2012; Zamudio et al., 2016). Over the last decades, a suite of methods has been proposed to infer species boundaries based primarily on patterns of genetic variation (Carstens et al., 2013). These methods include genotypic clustering algorithms (Pritchard et al., 2000), metrics of genealogical discordance (Cummings et al., 2008), tests of alternative species schemes under the multispecies coalescent model (Yang & Rannala, 2010), estimates of population gene flow (Smith & Carstens, 2020), and genetic‐based estimates of reproductive isolation (Singhal et al., 2018). With the promise of providing process‐based and objective inference of species boundaries (Fujita et al., 2012), molecular delimitation approaches have become a standard component of systematic and evolutionary investigations.

Many clades regarded as taxonomically challenging have now been scrutinized using large genetic datasets and sophisticated delimitation methods (e.g., Leaché et al., 2018; Pyron et al., 2020; Rivera et al., 2021; Wagner et al., 2013). Typically, multiple analytical approaches are combined in an integrative assessment of whether populations are on separate evolutionary trajectories. However, different approaches can yield incongruent inferences of species boundaries, leading some authors to advocate for consensus species partitions across multiple analytical frameworks (Carstens et al., 2013; Shaik et al., 2021). Still, objectively deriving a single scheme based on conflicting or inconclusive results can be difficult to impossible (e.g., Firneno et al., 2021; McKay et al., 2013; Tilley et al., 2013; Willis, 2017). Even in the face of the same patterns, different authors often disagree on the number of species involved (Burbrink & Ruane, 2021; de Queiroz, 2020; Hillis et al., 2021; Zachos et al., 2020). Moreover, the units emerging from delimitation algorithms might represent distinct populations rather than species (Sukumaran & Knowles, 2017). Finally, despite the increasing consensus around the definition of species as separately evolving metapopulation lineages (de Queiroz, 1998), translating conceptual definitions of species into empirical delimitation is not trivial (de Queiroz, 2007; Prates, Doughty, & Rabosky, 2022). Speciation is an extended and often non‐linear process, whereby species may constitute historical and geographic continuums that can be difficult to partition (Bouzid et al., 2022). Therefore, modern datasets and analytical techniques, integrative approaches, and conceptual advances have not always made species delimitation more accurate or objective (Barley et al., 2018; Garnett & Christidis, 2017; Hillis, 2019; Sites & Marshall, 2004; Sukumaran & Knowles, 2017).

Many existing taxonomies are based on morphological data, and limitations of these data might in part explain inconsistent or unclear species boundaries (Cadena & Zapata, 2021; Winker, 2009). However, such inconsistencies might also originate from patterns of genetic variation that map poorly onto how populations are represented in inferential frameworks. This poor mapping might result from biological processes. For instance, inferences of genetic structure and phylogenetic relationships can overestimate population differentiation in the presence of isolation‐by‐distance (Barley et al., 2018; Bradburd et al., 2018; Irwin, 2002), particularly if substantial sampling gaps exist (Battey et al., 2020). Moreover, genetic introgression can lead to oversplitting of species. Admixed populations can be mistaken as cryptic species due to unique allele combinations, particularly when admixture is geographically structured (Chan et al., 2020, 2021). Introgressive hybridization and incomplete allele sorting can also lead to heterogeneous divergence among genome regions. This heterogeneity hampers inferences of species boundaries and relationships, as reported for groups ranging from lizards to cichlid fishes and oak trees (Camargo et al., 2012; McVay et al., 2017; Turner et al., 2001). In addition, unclear species boundaries might result from processes that shape patterns of phenotypic variation. For instance, geographically segregated phenotypes (i.e., polytypism) owing to phenotypic plasticity or local adaptation can be interpreted as evidence of population separation, even when gene flow is high (Mayr, 1963; Zamudio et al., 2016). Conversely, morphological crypsis owing to developmental or ecological constraints can conceal genetically divergent species (Struck et al., 2018; Zamudio et al., 2016). These findings suggest that logistical challenges to species delimitation might emerge from particular evolutionary processes. Uncovering these processes might allow us to understand why certain clades have eluded, and perhaps are not amenable to, taxonomic categorization (Willis, 2017).

Here, we seek to understand the processes that might underlie “taxonomic disaster zones” — i.e., clades within which phenotypic and genotypic species boundaries are frequently unclear, inconsistent, or conflicting. To this goal, we focus on one of the most challenging Australian vertebrate clades: the *inornatus* group of the lizard genus *Ctenotus* (Scincidae). In this group, identifying species has long been regarded as difficult owing to ambiguous morphological diagnoses based on few or poorly understood characters, which, in turn, renders geographic ranges uncertain (Bush et al., 2007). Species in the *C. inornatus* group are a conspicuous and often dominant component of Australian arid zone lizard assemblages (James & Shine, 2000; Pianka, 1969, 1972, 1986; Rabosky et al., 2007, 2011). The 16 or so species‐level taxa commonly recognized in this group have been defined primarily based on dorsal coloration patterns, scalation, and body proportions (Horner & King, 1985; Storr, 1969, 1970, 1971, 1978; Storr et al., 1999). More recently, studies incorporating genetic data have suggested that variation in these traits does not necessarily reflect evolutionary divergence. For instance, a combined analysis of morphological characters, one mitochondrial DNA marker, and one nuclear DNA locus from more than 350 specimens inferred rampant taxon paraphyly, within‐species polymorphism, and among‐species sharing of characters previously thought to diagnose species (Rabosky et al., 2014). This scenario suggests that several traditionally recognized taxa in the *C. inornatus* species group lack genetic coherence or distinctiveness.

**FIGURE 1:**
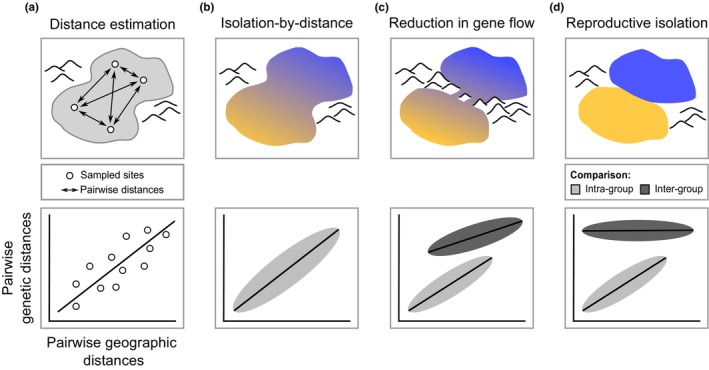
Expected relationships between genetic and geographic distances in the presence of one versus two species and implications for species delimitation. For dispersal‐limited organisms, genetic differentiation between sampled localities should increase as a function of geographic separation, corresponding to Wright's (1943) isolation‐by‐distance (IBD) model (a). In the absence of geographic or reproductive barriers, we should observe continuous IBD among conspecific populations (b). In contrast, in the presence of additional factors restricting gene flow (e.g., unsuitable habitat), the relationship between genetic and geographic distances may become discontinuous (c). In this case, two IBD patterns emerge from the same analysis: one for pairs of populations with unrestricted gene flow (light grey ellipse), and a second, offset relationship for pairs of populations with restricted gene flow (dark grey ellipse). Note that these clusters emerge from the data, irrespective of a priori assignments to candidate species or taxa. Finally, population differentiation can become uncoupled from geographic separation when sampling encompasses fully reproductively isolated units, regardless of the underlying geography (d). This latter case would provide strong evidence for two separately evolving species. [Colour figure can be viewed at wileyonlinelibrary.com]

In principle, the unexpected lack of genetic support for several taxa in the *C. inornatus* species group might reflect an over‐reliance on mitochondrial DNA and limited geographic sampling (Rabosky et al., 2014). Here, we revisit species limits with improved sampling of populations and sequencing of thousands of nuclear loci. Our approach comprises six steps. We start with analyses performed without reference to taxonomic assignments, namely (i) inferring phylogenetic relationships among hundreds of samples, and (ii) characterizing population genetic structure using genotypic clustering. We then (iii) compared the results with a mitochondrial analysis that incorporated additional samples beyond what was used in previous studies. Given evidence for multiple genetic units within particular widespread nominal taxa, we then (iv) tested whether inferred genetic breaks are explained by simple models of isolation‐by‐distance (Figure 1). This simple assessment is rarely included in delimitation studies but provides critical information on the extent to which genetic groups interpreted as putative species might emerge from spatial heterogeneity in sampling alone (Battey et al., 2020). Based on evidence of incomplete lineage separation and mitochondrial capture, we (v) test for introgression by determining if patterns of nuclear allele sharing exceed expectations from simple stochastic allele sorting. Finally, we (vi) estimate migration rates between pairs of putative species or geographically defined populations using historical demographic modelling. Based on the results, we discuss the biological underpinnings of fuzzy species boundaries in this and other challenging clades.

## 2 MATERIALS AND METHODS

### 2.1 Terminology and species criteria

This study uses the terms “taxa” and “taxon” to refer to the nominal entities currently recognized in *Ctenotus* taxonomy. “Traditional” and “traditionally recognized” taxa refer to definitions proposed based on morphological attributes (Horner & King, 1985; Storr, 1969, 1970, 1971, 1978). Six traditional taxa were deemed invalid by the combined genetic‐morphological analysis of Rabosky et al. (2014). These taxa are indicated between quotes: *C*. “*borealis*”, *C*. “*brachyonyx*”, *C*. “*fallens*”, *C*. “*helenae*”, *C*. “*saxatilis*”, and *C*. “*severus*”.

We consider “taxa” as operational classification devices that may or not correspond to “species” in an evolutionary sense. By “species”, we specifically refer to a conceptual category corresponding to separately evolving metapopulation lineages (de Queiroz, 1998, 2007). Applying this concept to species delimitation practice is often not trivial. Our approach employs operational taxonomic units (OTUs), which we delimit based on attributes widely proposed as *species criteria* (Cracraft, 1987; de Queiroz, 1998; Mallet, 2013, 2020; Mayr, 1963; Singhal et al., 2018). These criteria include: (1) conspecific individuals tend to cluster phylogenetically owing to shared derived genetic variants (i.e., branching patterns reflect species cohesiveness and distinctiveness); (2) conspecifics comprise a cohesive genotypic pool, sharing strongly correlated genome‐wide allele frequency patterns; (3) conspecifics span a coherent and mostly continuous geographic area, allowing gene flow between populations; and (4) regional differences in allele frequencies across a species' range follow a continuous isolation‐by‐distance pattern, without sharp breaks that might arise from geographic or reproductive barriers. We consider the OTUs (or “units”) delimited based on these criteria as candidate species.

We found multiple OTUs within four taxa: *C. inornatus*, *C. robustus*, *C. spaldingi*, and *C. superciliaris*. We refer to these intra‐taxon OTUs using labels that refer to their geographic ranges, such as *inornatus*‐N, *inornatus*‐S, and *superciliaris*‐W. Other taxa corresponded to a single OTU each; we refer to them using their corresponding taxon name: *burbidgei*, *eutaenius*, *lateralis*, *mastigura*, and *rimacola*.

### 2.2 Motivating case studies of taxonomic discord in the *Ctenotus inornatus* species group

Below, we briefly introduce four taxonomic issues to illustrate the confusion surrounding the *C. inornatus* species group and set up the structure of our analyses. Recently, the Australian Society of Herpetologists (ASH, 2022) recommended broad spatial and genomic sampling to properly address these issues.

### 2.3 What is *Ctenotus robustus*?

**FIGURE 2:**
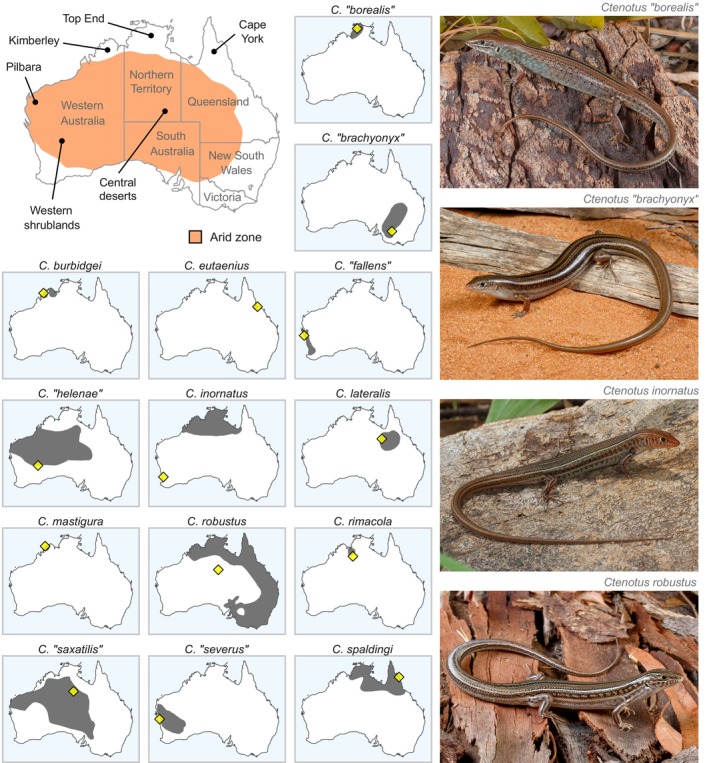
Location of geographic regions mentioned in this manuscript (upper left), presumed ranges of morphologically defined taxa traditionally recognized in the *Ctenotus inornatus* species group (smaller maps), and pictures of representative taxa (right). Maps of taxa not included in our analyses but otherwise presumed to belong to this species group (*C. capricorni* and *C. nullum*) were omitted. Dark polygons on taxon maps are expert‐validated ranges derived for the IUCN Red List Australian squamate assessment as compiled by Roll et al. (2017). Yellow diamonds indicate the type locality of each taxon based on the original descriptions. Note that the type localities of *C. robustus* and *C. inornatus* lie outside the broadly accepted ranges of these taxa. All lizard pictures by Jules Farquhar. [Colour figure can be viewed at wileyonlinelibrary.com]

*Ctenotus robustus* Storr, 1970 and *Ctenotus spaldingi* (Macleay, 1877) are morphologically similar taxa whose definitions and geographic ranges have been transformed by genetic evidence (Rabosky et al., 2014). *Ctenotus robustus* had been thought to encompass a broad arc from the Flinders Ranges (South Australia) to the Pilbara region (Western Australia), while *C. spaldingi* had been restricted to Australia's northeast (Queensland, Northern Territory) (Figure 2). However, a genetic analysis found nominal populations of *C. robustus* to be nested in a northwestern lineage, while nominal *C. spaldingi* occurs from northern (Cape York) to southern (Victoria) eastern Australia (Rabosky et al., 2014). Consequently, all eastern and southeastern populations of *C. robustus* were assigned to *C. spaldingi*. Adding to confusion, the presumed type locality of *C. robustus* lies outside this taxon's traditional range (Figure 2). We revisit the genetic limits between *C. robustus* and *C. spaldingi* and reassess recently proposed changes in their geographic distributions.

### 2.4 Is *Ctenotus borealis* just an atypical *C. robustus*?

*Ctenotus* “*borealis*” Horner & King, 1985 is a taxon from Australia's Top End that is morphologically similar to *C. robustus* but shows subtle coloration differences (Figure 2). This taxon was recently synonymized to *C. robustus* based on lack of differentiation in two loci (Rabosky et al., 2014), but this recommendation remains controversial (ASH, 2022). We employ genome‐wide data to assess whether these two names correspond to distinct lineages.

### 2.5 *Ctenotus superciliaris* and *C. “saxatilis”*: Same or different?

*Ctenotus* “*saxatilis*” Storr, 1970 is a widespread taxon (Figure 2) characterized by strongly marked dorsolateral stripes and spots. However, mitochondrial data suggest that this phenotype is shared by multiple divergent lineages in northern Australia (Figure 3 in Rabosky et al., 2014). One of these lineages is distinguished by its supraciliary scale configuration and was thus described as a new taxon, *Ctenotus superciliaris* Rabosky et al., 2014. However, Storr et al. (1999) claimed that this same character diagnoses *C*. “*saxatilis*”, albeit not mentioning it in this taxon's formal description (Storr, 1970). Rabosky et al. (2014) examined the holotype of *C*. “*saxatilis*” and found it to have the scalation pattern typical of *Ctenotus inornatus* (Gray, 1845) rather than that of *C. superciliaris*, thus synonymizing *C*. “*saxatilis*”. Increasing confusion, the name *C*. “*saxatilis*” remains widely used (ASH, 2022; Uetz et al., 2022). We employ genome‐wide data to test if *C. superciliaris* and *C*. “*saxatilis*” are genetically coherent and distinctive from *C. inornatus*.

**FIGURE 3:**
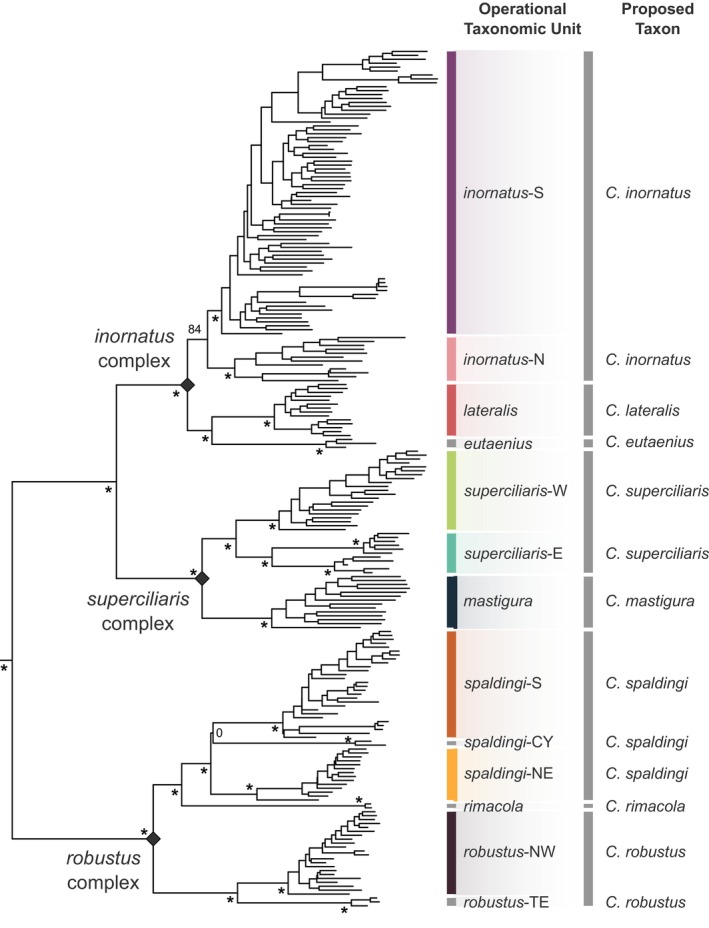
Overview of major inferred clades, operational taxonomic units (OTUs), and proposed provisional taxon scheme in the *Ctenotus inornatus* species group. Phylogenetic tree based on 524,324 base pairs from 3694 ddRAD loci (outgroups not shown). Asterisks indicate bootstrap nodal support >95. For clarity, only support for the OTUs and deeper relationships are shown. OTUs shown resulted from our delimitation analyses and are considered candidate species (see text). Pending detailed analyses of morphological variation and additional geographic sampling of the OTUs, we outline a provisional taxonomic scheme with the goal of supporting field and museum workers. [Colour figure can be viewed at wileyonlinelibrary.com]

### 2.6 Arid zone members of the *C. inornatus* complex: One species or five?

Four additional taxa were synonymized with *C. inornatus* (Rabosky et al., 2014): *Ctenotus* “*brachyonyx*” Storr, 1971, a striped taxon from the southeastern deserts; *Ctenotus* “*fallens*” Storr, 1973, with dorsal stripes and lateral spotting, from the western coast; *Ctenotus* “*helenae*” Storr, 1969, a weakly patterned taxon from the central deserts; and *Ctenotus* “*severus*” Storr, 1969, with lateral elements but no vertebral or paravertebral stripes, from the western deserts (Figure 2). Individuals with the traits presumed to diagnose each of these taxa did not cluster in a mitochondrial tree, suggesting that the group's taxonomy might be confounded by evolutionary lability in coloration (e.g., Figure 7 in Rabosky et al., 2014). However, these names remain broadly recognized (ASH, 2022; Uetz et al., 2022). We investigate whether each of them comprises a coherent and divergent genetic pool.

### 2.7 Genetic sampling

Our nuclear dataset included 13 ingroup taxa under the traditional morphological taxonomy (Storr et al., 1999), corresponding to seven taxa following Rabosky et al. (2014). The original identification of specimens in our molecular dataset (as made by field collectors and museum staff, including ourselves) largely applied that traditional scheme, as follows (numbers in parentheses indicate the number of samples originally assigned to each taxon): *C*. “*borealis*” (four specimens), *C*. “*brachyonyx*” (five), *C. eutaenius* (three), *C*. “*fallens*” (three), *C*. “*helenae*” (52), *C. inornatus* (28), *C. lateralis* (15), *C. mastigura* (one), *C. rimacola* (two), *C. robustus* (55), *C*. “*saxatilis*” (31), *C*. “*severus*” (three), and *C. spaldingi* (16). As outgroups, we included representatives of other *Ctenotus* species groups and the closely related genus *Lerista*: *C. atlas* (two), *C. australis* (four), *C. essingtonii* (two), *C. leonhardii* (two), *C. nigrilineatus* (two), *C. pantherinus* (two), *C. schomburgkii* (two), *C. taeniolatus* (two), *L. bipes* (two), and *L. ips* (two) (Table S1 provides nuclear DNA sample information).

Our analyses incorporated a double‐digest restriction site‐associated (ddRAD) dataset generated by comprehensive analyses of sphenomorphine skinks (Prates, Singhal, et al., 2022; Singhal et al., 2017) and available in the Sequence Read Archive (BioProjects PRJNA755251 and PRJNA382545). Briefly, DNA was digested with the restriction enzymes EcoRI and MspI, tagged with individual barcodes, size‐selected (150–250 bp), PCR‐amplified, multiplexed, and sequenced on an Illumina platform. We used *ipyrad* v. 0.9.84 (Eaton & Overcast, 2020) to demultiplex reads (allowing no mismatches from individual barcodes), perform de novo assembly (minimum clustering similarity = 0.90), align loci, and call single nucleotide polymorphisms (SNPs). We enforced a minimum Phred quality score (=33), sequence coverage (=6x), and read length (=35 bp); and a maximum proportion of heterozygous sites per locus (=0.5) and number of alleles per nucleotide site within an individual (=2, i.e., a diploid genome). After these steps, we generated a final dataset for phylogenetic inference by retaining loci present in at least 30% of the sampled individuals. The final phylogenetic dataset included 524,324 base pairs from 3694 ddRAD loci. Moreover, to reduce missing data in population genetic analyses, we generated three datasets corresponding to each of three major inferred clades (“species complexes”; see Results), retaining loci present in at least 50% of the sampled individuals. The final population genetic datasets included 6287 (*spaldingi* complex), 7447 (*superciliaris* complex), and 3534 (*inornatus* complex) unlinked SNPs.

This nuclear phylogenetic dataset included a total of 242 specimens. To further expand our sampling, we also incorporated a mitochondrial dataset comprising 485 samples (see Table S2 for sample information; see Figure S1 and Figure S2 for the geographic localities of samples partitioned by delimited OTU and proposed taxon respectively). Of those, 348 samples were sequenced by previous efforts (Rabosky et al., 2011, 2014). Besides broader geographic sampling for each taxon, this dataset included *Ctenotus burbidgei* Storr, 1975, for which nuclear data were not available. We amplified, sequenced, edited, and aligned a 1143 base pair fragment of the cytochrome B gene following standard protocols (Rabosky et al., 2009). Newly generated mitochondrial sequences were uploaded to GenBank (accessions numbers OQ091785–OQ091921; Table S2).

### 2.8 Inferring phylogenetic relationships

We inferred evolutionary relationships based on the nuclear and mitochondrial datasets separately. Phylogenetic inference incorporated both variant and invariant sites under maximum likelihood using RAxML‐HPC v. 8.2.12 (Stamatakis, 2014) and employing the GTRCAT model of nucleotide evolution. For this analysis, loci were concatenated, and polymorphic sites were coded as ambiguities. We also inferred a nuclear SNP‐based tree under the multispecies coalescent framework using SVD Quartets (Chifman & Kubatko, 2014) as implemented in the command line version of PAUP v. 4 (Swofford, 2002). This analysis incorporated one SNP from each locus for a total of 3692 SNPs and sampled all possible quartets. In both phylogenetic analyses, we estimated node support based on 1000 bootstraps.

### 2.9 Inferring genetic structure

We estimated patterns of nuclear admixture and allele sharing using sNMF, a genotypic clustering method that does not assume Hardy–Weinberg equilibrium to identify clusters (Frichot et al., 2014). To avoid the spurious grouping of densely sampled localities (Lawson et al., 2018; Puechmaille, 2016), we limited the maximum number of samples per collecting site to five. In these analyses, we dropped four highly divergent lineages; although we consider these lineages as OTUs (see Results), they are represented by <4 samples, and small sample sizes can lead to spurious grouping of samples in genotypic clustering analyses (Lawson et al., 2018; Puechmaille, 2016). We removed SNPs with a minimum allele frequency <0.05 (within each of the three complexes) to improve the inference of population structure (Linck & Battey, 2019) and minimize spurious SNPs from sequencing errors (Ahrens et al., 2018) using VCFtools v. 0.1.16 (Danecek et al., 2011). After extracting a single SNP per locus, individuals that had data for less than 50% of the final SNPs were excluded. Outgroups were not included in these analyses. We ran sNMF using the *LEA* R package (Frichot & François, 2015). Preliminary analyses supported that the number of inferred clusters is robust to the regularization parameter in sNMF; this parameter was set to 500 in the final analyses. The tolerance parameter was set to the default value (0.00001). To infer the best‐fitting number of genotypic clusters (K), we compared K = 1–10 with 20 replicates for each K. The K value that yielded the lowest cross‐entropy value across replicates was considered to be the best‐fit K.

We then confirmed whether samples inferred in the same genotypic cluster also group in genotypic space. For that purpose, we performed a Principal Component Analysis on the unlinked SNP data using the *LEA* R package and inspected biplots of principal components.

### 2.10 Testing the robustness of results to isolation‐by‐distance

Phylogenetic and population genetic structure are widely employed as indicative of species divergence and boundaries. However, genetic breaks can emerge over the range of a continuously distributed species due to isolation‐by‐distance (IBD), despite high gene flow connecting adjacent locations (Irwin, 2002; Wright, 1943). Thus, we investigated whether IBD alone can account for the population structure emerging from our genetic analyses.

Methods are available that can account for IBD while performing genotypic clustering (Bradburd et al., 2018), but these approaches were not computationally tractable for our large dataset. Thus, we assessed IBD patterns based on pairwise *F*_ST_ (Weir & Cockerham, 1984; Weir & Hill, 2002), applying the following reasoning. Isolation‐by‐distance is the relationship described by genetic (here, *F*_ST_) versus geographic distances (Irwin, 2002; Wright, 1943) (Figure 1a). Under simplifying assumptions (e.g., equilibrium demography, landscape homogeneity), we expect the IBD relationship between populations of the same species to follow a simple linear relationship (Figure 1b), whose slope is a function of population density and effective gene dispersal (Rousset, 1997). In this case, geographic separation alone can explain genetic differentiation, reflecting the continuous decay of population connectivity over a species' range. By contrast, when a single analysis includes populations from distinct species, we may expect the IBD relationship within and between populations to be discontinuous (i.e., to be described by multiple curves) (Figure 1c). In some cases, populations could be highly differentiated with minimal effects of geographic separation on levels of genetic differentiation (Figure 1d). This situation points to mechanisms that restrict gene flow in parapatry, such as reproductive isolation, and, as such, supports delimitation of species. To determine which patterns best match our taxa, we estimated pairwise individual *F*_ST_ based on the SNP data using the *BEDASSLE* R package (Bradburd et al., 2013). To calculate a matrix of geographic distances, we used the R package *fossil* (Vavrek, 2011).

### 2.11 Modelling demographic history

To characterize gene flow among populations, we performed historical demographic inference using G‐PhoCS v. 1.3 (Gronau et al., 2011), implementing an isolation‐with‐migration model (Nielsen & Wakeley, 2001; Pinho & Hey, 2010). We estimated gene flow between three classes of population pairs (see Figure S3 for their geographic locations):

1. Pairs of populations corresponding to the OTUs resulting from the phylogenetic and genotypic clustering analyses. This set often included pairs of populations that are sympatric and divergent, thus providing estimates of gene flow across candidate species.
2. Pairs including populations corresponding to morphology‐defined taxa that were recently synonymized based on multi‐locus data (Rabosky et al., 2014). This set aims to assess the degree of separation of populations assigned to *C*. “*borealis*”, *C*. “*brachyonyx*”, *C*. “*fallens*”, and *C*. “*severus*” relative to closely related samples from adjacent geographic regions but assigned to another taxon. We defined four such adjacent populations: Pilbara, central deserts, western shrublands, and northern Australia.
3. Pairs corresponding to a single population randomly split into two. This set aims to provide a reference of panmixia by artificially separating samples from the same lineage and geographic region.

G‐PhoCS imposes prior distributions (given by shape, α, and rate, β) on three classes of population genetic parameters: θ (=4Nμ, where N corresponds to the effective population size and μ to the per‐generation mutation rate); the splitting time parameter τ (=Tμ, where T corresponds to the tree height in number of generations); and m (=M/μ, where M corresponds to the proportion of individuals in one population that originated from another population in each generation) (note that these migration parameter definitions follow the developers of G‐PhoCS; see supplementary Material in Gronau et al., 2011). For θ and τ, we used gamma (shape = 1, rate = 100) prior densities. For m, we used a migration band in each direction under a gamma (shape = 0.001, rate = 0.00001) prior. Prior distributions were chosen to encompass a wide range of biologically plausible population histories (i.e., effective population sizes ranging from tens of thousands to millions, migration levels ranging from zero to high enough to constrain population divergence; an R script used to help define prior distributions is provided in GitHub). We then simulated posterior parameter distributions using Markov chain Monte Carlo (MCMC). We ran two MCMC simulations per pair, each consisting of 300,000 steps, sampling every 100 steps, and discarding the first 25% of the steps as burn‐in. Chain stationarity and convergence were confirmed by plotting parameter traces in R. Fine‐tuning parameters controlling MCMC acceptance rates were defined automatically. Owing to computational times, G‐PhoCS analyses used a maximum of 12 samples per population and of 4500 loci (which included both invariant and variant sites).

To convert mutation rate‐scaled estimates of population genetic parameters into absolute estimates, we assumed 7.6 × 10^−9^ substitutions per site per year in lizards (Gottscho et al., 2017) and a generation time of 2 years based on an age at maturity of 22 months in *C*. “*helenae*” (James, 1991a, 1991b). Based on posterior parameter estimates, we calculated the effective number of gene migrations received by a population per generation, 2NM, also known as the population migration rate. Theory predicts that, when 2NM is greater than 1, divergence between constituent subpopulations will be constrained (Nielsen & Slatkin, 2013; Pinho & Hey, 2010; Wright, 1931). Given caveats pertaining to 2NM estimates (Whitlock & McCauley, 1999), we interpret the results carefully within the broader scenario of introgression emerging from the phylogenetic, genotypic clustering, IBD, and ABBA‐BABA analyses.

### 2.12 Estimating excess allele sharing from introgression

Finally, to further test nuclear introgression among candidate species, we estimated two ABBA‐BABA‐class statistics (Green et al., 2010): Patterson's D (Patterson et al., 2012) and f‐branch (Malinsky et al., 2018). These approaches employ a four‐population tree with structure (((P2, P1), P3), O), where O is an outgroup. Typically, many ancestral (A) and derived (B) alleles show a BBAA structure (with allele ordering following the tree given above). However, incomplete lineage sorting leads to ABBA and BABA patterns, which should occur in equal frequencies. Introgression between P3 and P1 or P2 leads to an excess of ABBA or BABA patterns, which is captured by D and related statistics. One limitation of this approach is that multiple closely related populations can appear introgressed owing to a single introgression event involving a common ancestor. To minimize this issue, the f‐branch metric accounts for correlated allele frequencies among populations. This approach allows identifying gene flow events involving the internal branches of a tree, which correspond to the ancestors of sampled populations (Malinsky et al., 2018). We estimated D and f‐branch metrics based on the unlinked SNP data (i.e., one SNP per locus) in Dsuite (Malinsky et al., 2021), using the Benjamini‐Hochberg correction to control for false‐discovery rates (Benjamini & Hochberg, 1995) and estimating significance using a block‐jackknifing approach (Durand et al., 2011; Green et al., 2010). We considered all combinations of candidate species given constraints from the estimated coalescent‐based phylogenetic tree and used *C. essingtonii* as an outgroup.

## 3 RESULTS

### 3.1 Overall phylogenetic patterns

Phylogenetic trees of the *C. inornatus* species group based on nuclear DNA under maximum likelihood on the concatenated loci (Figure 3) or using a coalescent‐based approach on unlinked SNPs (Figure S4) identified three major clades, which we informally refer to as the *inornatus*, *robustus*, and *superciliaris* complexes (Figure 3). We comment on patterns of genetic structure and admixture within each of these complexes separately.

### 3.2 Lineage delimitation in the *robustus* complex

**FIGURE 4:**
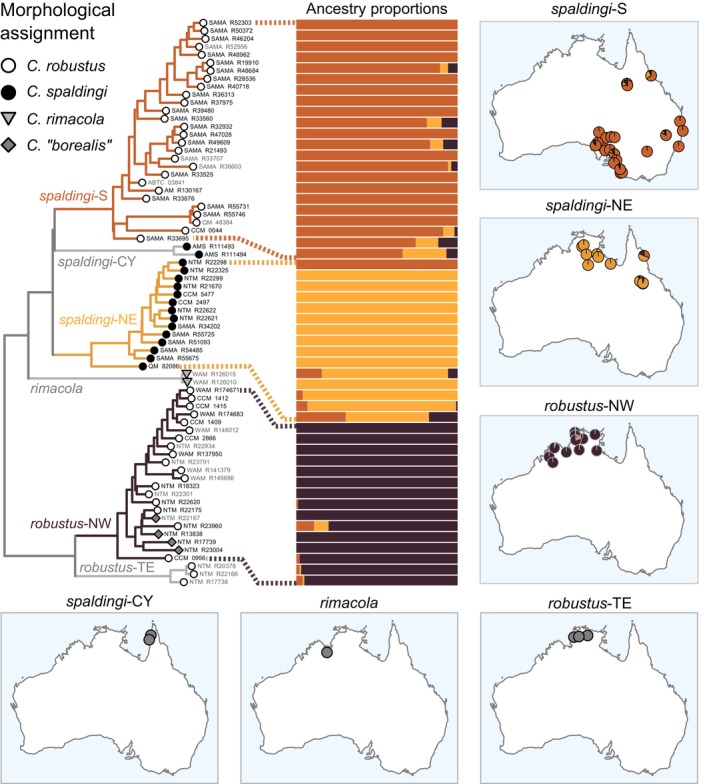
Genotypic clustering results (bars) and phylogenetic support for traditionally recognized taxa in the *robustus* complex. Nuclear phylogenetic relationships (left) as in Figure 3 but pruned to this complex. Individuals shown in grey tip labels were not included in the clustering analysis due to high missing data or scarce sampling of the corresponding candidate species. Pie charts on maps indicate the average ancestry proportions corresponding to each cluster at each site. Clustering results support two clusters corresponding to the taxon *C. spaldingi*: *spaldingi*‐S and *spaldingi*‐NE, which might overlap in northeastern localities. Unit *robustus‐*NW from northern‐northwestern Australia corresponds to a subset of the taxon *C. robustus*. *Ctenotus rimacola*, *robustus‐TE*, and *spaldingi*‐CY are highly divergent from other units and considered candidate species, yet not included in clustering analyses owing to scarce sampling. Note that, despite a wide geographic sampling gap between northern and southern populations of *spaldingi*‐S, our analyses did not support them as independent genetic groups. [Colour figure can be viewed at wileyonlinelibrary.com]

The *robustus* complex contains six deeply divergent and well‐supported lineages that we consider operational taxonomic units (OTUs) (Figure 3). Three of these lineages were well‐sampled and included in genotypic clustering analyses; each corresponded to a cluster (Figure 4). One such cluster spans a large latitudinal range in eastern Australia (northern Queensland to Victoria); we refer to it as the OTU *spaldingi*‐S (dark orange in Figure 4). Another OTU, *spaldingi*‐NE (light orange in Figure 4), occurs in northern and northeastern Australia (Northern Territory, Queensland). A third OTU, *robustus*‐NW (maroon in Figure 4), occurs along northern and northwestern Australia reaching the Pilbara region. PCA analyses on the unlinked SNP data confirmed that samples inferred in the same cluster grouped together in genotypic space. Moreover, each inferred cluster occupied a distinct portion of genotypic space (Figure S5).

**FIGURE 5:**
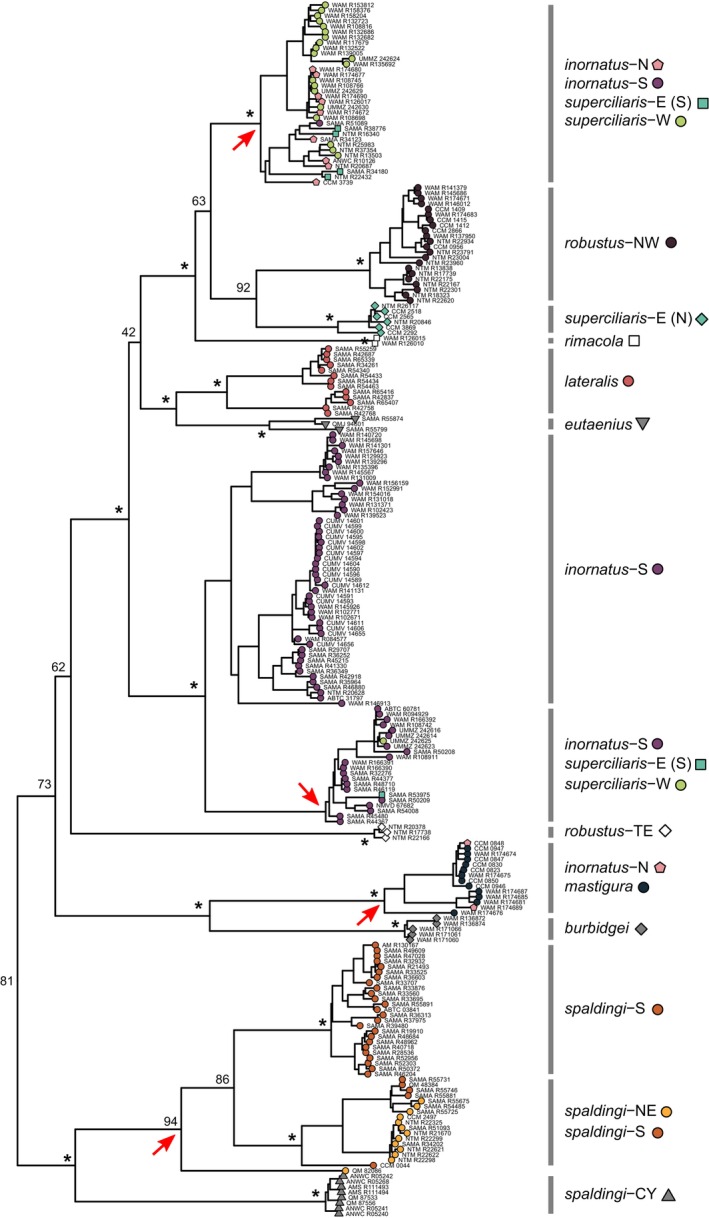
Phylogenetic relationships in the *Ctenotus inornatus* group based on the *cytochrome B* mitochondrial marker. The colours of tip symbols correspond to the operational taxonomic units (OTUs) delimited based on nuclear loci. For clarity, only samples with matching nuclear data are shown, except for individuals of *C. burbidgei* and the OTU *spaldingi*‐CY, which had scarce or no nuclear data (for a complete mitochondrial tree, see Figure S6). Asterisks indicate bootstrap nodal support >95. For clarity, only support for the OTUs and deeper relationships are shown. There were notable instances of mitochondrial paraphyly of nuclear OTUs (lineages involved are indicated with red arrows). In particular, a mitochondrial lineage grouped samples of four nuclear OTUs from two distinct species complexes: *inornatus*‐N, *inornatus*‐S, *superciliaris*‐E, and *superciliaris*‐W. Note also that *superciliaris*‐E was split into two distant mitochondrial lineages, each matching a divergent nuclear lineage (Figure 8). Similarly, *robustus*‐NW and *robustus*‐TE, sisters in the nuclear tree (Figure 4), formed distant mitochondrial lineages. [Colour figure can be viewed at wileyonlinelibrary.com]

To avoid spurious grouping of sparsely sampled but highly divergent lineages, we removed three highly divergent lineages from our clustering analysis. However, our phylogenetic analyses of both mtDNA and nuclear SNPs support these lineages as distinct OTUs (Figures 4 and 5). Among them is a lineage composed of northern Australian specimens morphologically assigned to *Ctenotus rimacola* Horner & Fisher, 1998. Another such lineage is the sister of *robustus*‐NW, which we refer to as *robustus‐*TE. This OTU is sympatric with *robustus*‐NW in the Top End region and thus likely represents a separate species. OTUs *robustus‐*NW and *robustus‐*TE composed coherent but non‐sister mitochondrial lineages (Figure 5; Figure S6). Another highly divergent lineage occurs in the Cape York Peninsula; we refer to it as *spaldingi*‐CY. Mitochondrial results further support that this OTU is highly divergent. We note that the relative position of *spaldingi*‐CY was the only difference between the concatenated and coalescent‐based nuclear trees; this OTU was inferred as sister to *spaldingi*‐S under concatenation, but as sister to *spaldingi*‐NE under a coalescence‐based framework (Figure S4).

**FIGURE 6:**
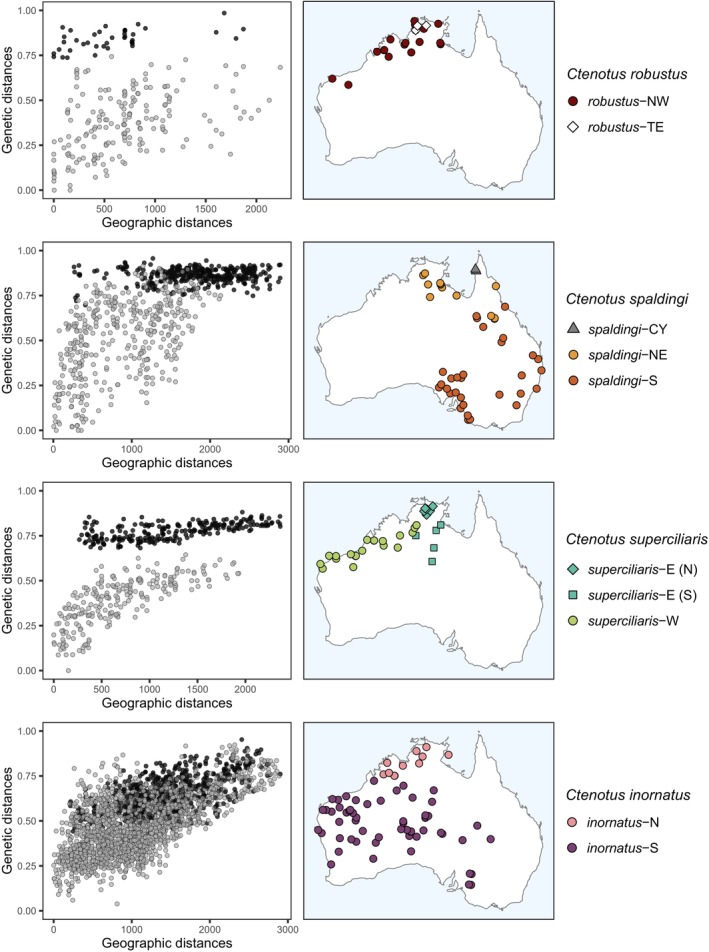
Pairwise *F*_ST_ between individuals from the same (grey) or different (black) groups, as a function of the geographic distances between them. Groups are the operational taxonomic units (OTUs) delimited within currently recognized taxa, whose ranges are shown on maps. Genetic and geographic distances within and between groups should form a continuous relationship when these groups correspond to the same species (see Figure 1). This appears to be the case for OTUs corresponding to the taxon *Ctenotus inornatus*. By contrast, the relationship between genetic and geographic distances within and between groups should be discontinuous in the presence of separate species (Figure 1). This appears to be the case of OTUs within the taxa *C. spaldingi*, *C. robustus*, and *C. superciliaris*. [Colour figure can be viewed at wileyonlinelibrary.com]

To assess whether evidence of distinct genetic pools across regions might simply reflect isolation‐by‐distance (IBD), we estimated genetic distances across space within and between the delimited OTUs. The results further support that OTUs in the *robustus* complex correspond to separate species. For instance, the relationship between genetic and geographic distances (i.e., the IBD pattern) within and between *robustus*‐NW and *robustus*‐TE, which are sympatric in northern Australia, was largely discontinuous (Figure 6). This pattern of genetic differentiation decoupled from levels of geographic separation points to mechanisms limiting gene flow across distinct species (Figure 1). A similar pattern was observed between *spaldingi*‐CY, *spaldingi*‐NE, and *spaldingi*‐S, which, based on current sampling, appear parapatric in northeastern Australia (Figure 6). These results further support that these OTUs correspond to separate species in an evolutionary sense, even though we conservatively treat them as intra‐taxon units (Figure 3).

**FIGURE 7:**
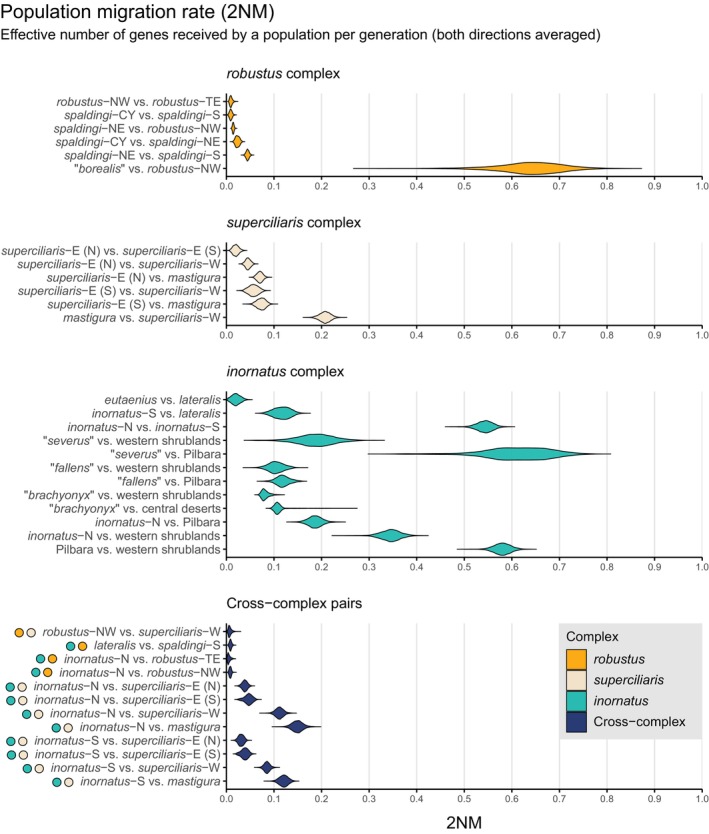
Estimates of population migration rates (2NM), the effective number of gene migrations received by a population per generation. Each violin plot corresponds to a pair of populations, including (i) the operational taxonomic units (OTUs) inferred in this study, (ii) traditional taxa of disputed validity, and (iii) populations geographically adjacent to these taxa. In each pair, values are averages between the two directions of gene flow (see Table S3 for all estimates). To facilitate comparison, x‐axes span the same 2NM range across plots. OTUs in the same complex showed relatively low (e.g., *spaldingi*‐CY and *spaldingi*‐S) to high (e.g., *inornatus*‐N and *inornatus*‐S) gene flow. We also find low (*C*. “*fallens*”, *C*. “*brachyonyx*”) to high (e.g., *C*. “*borealis*”, *C*. “*severus*”) absolute numbers of gene migrations among traditional taxa and their respective geographically adjacent populations. Note that gene flow levels between OTUs from different complexes (i.e., divergent species; dark blue violins) were often comparable or higher than intra‐complex estimates. [Colour figure can be viewed at wileyonlinelibrary.com]

Consistent with the IBD patterns, historical demographic modelling suggests low gene flow across candidate species in the *robustus* complex (Figure 7). Population migration rates (2NM) approached zero in both directions between all OTUs in this complex (median value range = 0.01–0.07), including sympatric pairs. This was the case, for instance, of the pairs comprising *robustus*‐NW, *robustus*‐TE, and *spaldingi*‐NE, all of which co‐occur in the Top End (Table S3).

### 3.3 Support for traditional taxa in the *robustus* complex

We also assessed whether traditionally recognized taxa are consistent with the genetic patterns. Figure 4 (left) shows the morphology‐based assignment of individuals to taxa relative to the inferred tree. The results support that the traditional (i.e., morphology‐based) taxa are broadly paraphyletic. For instance, specimens assigned to *C. robustus* (white circles in Figure 4) were found nested in two non‐sister lineages, corresponding to *robustus*‐NW and *spaldingi*‐S. Both of these OTUs occur far from the presumed type locality of *C. robustus* (Figure 2), previously associated with the northern lineage (Rabosky et al., 2014). Similarly, samples morphologically assigned to *C. spaldingi* (black circles in Figure 4) formed two non‐sister lineages, corresponding to *spaldingi*‐CY and *spaldingi*‐N. Likewise, samples identified as *C*. “*borealis*” (dark grey in Figure 4) were nested among geographically adjacent samples identified as *C. robustus*, both corresponding to *robustus*‐NW. Historical demographic analyses inferred high gene flow between populations assigned to *C*. “*borealis*” and *C. robustus* (Figure 7), with relatively high 2NM between them in both directions (0.46 and 0.82).

Pending analyses of morphological variation and additional geographic sampling to formally describe new taxa based on the inferred OTUs, we provisionally assign the OTUs to existing taxon names (Figure 3). In doing so, we aim to provide labels that reflect evolutionary relationships while minimizing change relative to the most recent assessment (Rabosky et al., 2014). We refer the populations corresponding to *spaldingi*‐CY, *spaldingi*‐S, and *spaldingi*‐N to the taxon *C. spaldingi*. OTUs *robustus*‐NW and *robustus*‐TE are referred to *C. robustus*. This definition of *C. robustus* excludes populations from eastern and southern Australia traditionally assigned to this name (Figure 2) but found to correspond to the *C. spaldingi* lineage (Figure 4), corroborating the findings of Rabosky et al. (2014).

### 3.4 Lineage delimitation in the *superciliaris* complex

**FIGURE 8:**
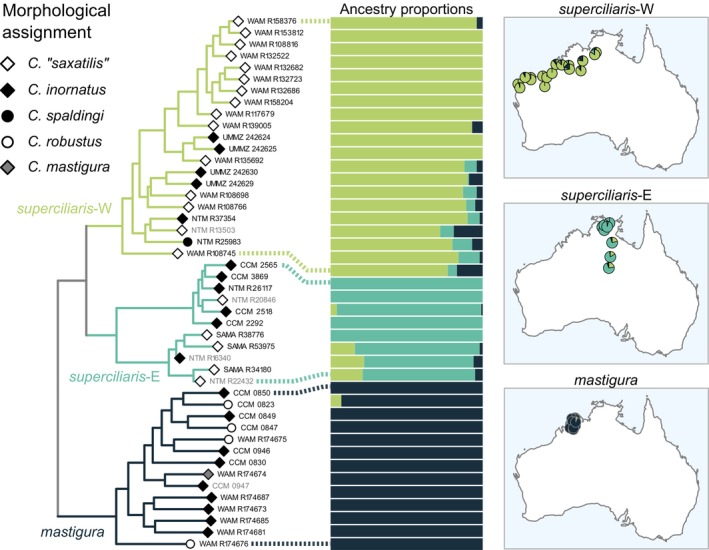
Genotypic clustering results (bars) and phylogenetic support for traditionally recognized taxa in the *superciliaris* complex. Nuclear phylogenetic relationships (left) as in Figure 3 but pruned to this complex. Individuals shown in grey tip labels were not included in the clustering analysis due to high missing data. Pie charts on maps indicate the average ancestry proportions corresponding to each cluster at each site. Genotypic clustering identified three groups that may overlap geographically. Units *superciliaris*‐W and *superciliaris*‐E correspond to the taxon *C. superciliaris*. [Colour figure can be viewed at wileyonlinelibrary.com]

The *superciliaris* complex contains three well‐supported lineages that we treat as OTUs (Figure 3); each corresponds to a genotypic cluster (Figure 8). One such OTU spans Australia's northwestern coast into the northern interior (Western Australia, Northern Territory); we refer to it as *superciliaris*‐W (green in Figure 8). This OTU is sister to *superciliaris*‐E, which occurs from the Top End into the central deserts (Northern Territory) (cyan in Figure 8). The third OTU occurs in the Kimberley, a western Australian region scarcely represented in previous analyses (dark blue in Figure 8). This OTU appears to correspond to the taxon *Ctenotus mastigura* Storr, 1975. Our mitochondrial analysis inferred this putative *C. mastigura* to be the sister of *C. burbidgei* (Figure 5; Figure S6), a taxon also from the Kimberley not represented in the nuclear dataset, in agreement with their presumed close relationships based on morphology (Storr, 1975).

Similar to the *robustus* complex, IBD analyses suggest that delimited OTUs in the *superciliaris* complex correspond to separate species. The relationship between genetic and geographic distances within and between OTUs was largely discontinuous, with genetic differentiation largely independent from geographic separation (Figure 6). IBD patterns also suggest that *superciliaris‐*E might correspond to two separate units, one restricted to the Top End and another further south (see Figure S7). Consistent with this scenario, the mitochondrial analysis inferred samples corresponding to each of these two regions as nested in divergent lineages (Figure 5; Figure S6). Population migration rate estimates (2NM) suggest relatively low gene flow among OTUs in the *superciliaris* complex (0.08–0.21), consistent with multiple separate species.

### 3.5 Support for traditional taxa in the *superciliaris* complex

**FIGURE 9:**
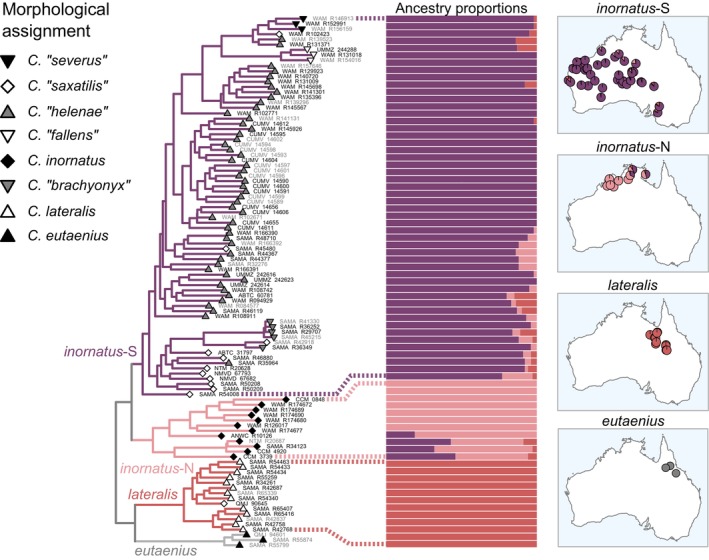
Genotypic clustering results (bars) and phylogenetic support for traditionally recognized taxa in the *inornatus* complex. Nuclear phylogenetic relationships (left) as shown in Figure 3 but pruned to this complex. Individuals shown in grey tip labels were not included in the clustering analysis due to high missing data or scarce sampling of the corresponding candidate species. Pie charts on maps indicate the average ancestry proportions corresponding to each cluster at each site. Genotypic clustering identified two clusters corresponding to the taxon *C. inornatus* (sensu Rabosky et al. (2014)): *inornatus*‐N and *inornatus*‐S, which nonetheless establish substantial gene flow (see text). *Ctenotus eutaenius* is highly divergent from other units but not included in clustering analyses owing to scarce sampling. Note that unit *inornatus*‐S, spanning most of Australia's central arid zone, grouped specimens traditionally assigned to several distinct taxa based on coloration patterns. [Colour figure can be viewed at wileyonlinelibrary.com]

As in the *robustus* complex, we find paraphyly of traditionally recognized taxa in the *superciliaris* complex (Figure 8). The original morphological identification of specimens mostly corresponded to *C. inornatus* and *C*. “*saxatilis*”. These two taxa were inferred as broadly paraphyletic, with samples scattered throughout the *superciliaris* complex and also the *inornatus* complex (Figure 9). Previous morphological examinations established that the name‐bearing types of *C. inornatus* and *C*. “*saxatilis*” are *inornatus* complex specimens (Rabosky et al., 2014). Pending morphological assessments to support the description of new taxa from the OTUs, we refer the populations corresponding to *superciliaris*‐W and *superciliaris*‐E to the taxon *C. superciliaris*.

### 3.6 Lineage delimitation in the *inornatus* complex

The *inornatus* complex contains four well‐supported lineages, here treated as OTUs (Figure 3). Three of them were well‐sampled and included in the genotypic clustering analyses; each corresponded to a cluster (Figure 9). One such OTU occurs in Australia's northeast (Queensland), corresponding to the taxon *Ctenotus lateralis* Storr, 1978 (red in Figure 9). This lineage is sister to a highly divergent lineage from eastern Queensland that appears to correspond to *Ctenotus eutaenius* Storr, 1981. A third lineage occurs in the Top End and Kimberley regions (Western Australia, Northern Territory); we refer to it as *inornatus*‐N (pink in Figure 9). This OTU is sister to a lineage spanning most of Australia's arid zone, which we refer to as *inornatus*‐S (purple in Figure 9).

Contrasting with patterns in the *robustus* and *superciliaris* complexes, IBD relationships within and between groups were highly overlapping and nearly continuous across certain OTUs in the *inornatus* complex (Figure 6). Geographic separation alone appears to account for most of the genetic differentiation between *inornatus*‐N and *inornatus*‐S, consistent with a gradient of population connectivity over the range of a single species (Figure 1). In agreement with this finding, we estimated substantial gene flow between *inornatus*‐N and *inornatus*‐S, with the highest population migration rates (2NM) relative to all other OTU pairs (median values in each direction = 0.51 and 0.58). Gene flow between *inornatus*‐N and *inornatus*‐S was higher than that between other parapatric OTUs in the *inornatus* complex, such as the pairs *C. eutaenius* and *C. lateralis* or *inornatus*‐S and *C. lateralis* (Figure 7). These results support that *inornatus*‐N and *inornatus*‐S correspond to the same evolutionary species, albeit one with detectable population structure.

### 3.7 Support for traditional taxa in the *inornatus* complex

As in the *robustus* and *superciliaris* complexes, traditionally recognized taxa in the *inornatus* complex showed widespread paraphyly. This complex includes many samples morphologically assigned to *C*. “*saxatilis*” and *C. inornatus*, two taxa scattered throughout this (Figure 9) and the other (Figures 4 and 8) complexes. We also inferred polyphyly among several taxa corresponding to regional coloration phenotypes. Namely, *C*. “*helenae*” was interspersed among samples of *C. inornatus*, *C*. “*fallens*”, and *C*. “*severus*”. Among them, *C*. “*fallens*” and *C*. “*severus*” grouped in their own lineages, yet nested among *C*. “*helenae*” and *C*. “*saxatilis*”. In turn, *C*. “*brachyonyx*” formed a paraphyletic assemblage with *C*. “*saxatilis*”. We note that genotypic clustering grouped *C*. “*brachyonyx*”, *C*. “*helenae*”, *C. inornatus*, *C*. “*saxatilis*”, and *C*. “*severus*” into a single cluster, corresponding to OTU *inornatus*‐S (Figure 9). This finding suggests extensive allele sharing across the arid zone despite regional coloration variation, broadly agreeing with findings based on few loci (Rabosky et al., 2014).

To further assess the coherence and distinction of those recently disputed taxa, we estimated population migration rates between them and samples from adjacent geographic regions. We considered four such regions: western shrublands, Pilbara, central deserts (all corresponding to *inornatus*‐S), and a northern population (corresponding to *inornatus*‐N). Samples from these regions appear to correspond to the same species (see above), providing a reference of intraspecific gene flow levels. This exercise revealed relatively high gene flow between *C*. “*severus*” and samples from the adjacent western shrublands and Pilbara (Figure 7); in both cases, estimates were asymmetrical, with higher numbers of migrant genes out of *C*. “*severus*” (0.34–1.23) than into it (0–0.03). These estimates were comparable to those among localities presumed to correspond to the same species, such as the pair including the western shrublands and Pilbara samples (0.23 and 0.93) or that including the northern and Pilbara samples (0.15 and 0.22) (Figure 7; Table S3).

By contrast, the number of gene migrations was relatively lower between other taxa and their adjacent populations. This was the case of *C*. “*brachyonyx*” relative to both the central deserts and western shrublands. Gene flow was also asymmetrical in this case, higher into *C*. “*brachyonyx*” (0.15–0.21) than out of it (0–0.01). Similarly, we inferred higher gene migrations into *C*. “*fallens*” (0.10–0.16) than out of it with both the Pilbara and western shrublands samples (0.08–0.11).

Random partitions of single populations yielded 2NM higher than 1 (1.57–2.89) (Table S3), consistent with theoretical expectations of populations whose divergence is constrained by high gene flow (Nielsen & Slatkin, 2013; Pinho & Hey, 2010; Wright, 1931).

### 3.8 Evidence of introgression across distantly related lineages

Our phylogenetic and genetic clustering results revealed evidence of admixture between species from distinct complexes. For instance, we found highly similar or shared mitochondrial haplotypes between distantly related nuclear OTUs (Figure 5; Figure S6), which might indicate allele capture across species boundaries. An extreme case was that of a mitochondrial lineage grouping samples from four nuclear OTUs representing two complexes: *inornatus*‐N, *inornatus*‐S, *superciliaris*‐E, and *superciliaris*‐W (uppermost lineage in Figure 5). Notably, these four OTUs co‐occur in northern Australia.

Gene flow estimates based on the nuclear loci further supported that certain distantly related OTUs are mutually introgressed (Figure 7). For instance, we inferred some gene flow between *inornatus‐*N (*inornatus* complex) and putative *C. mastigura* (*superciliaris* complex) (0.13 and 0.16 in each direction) (Table S3). These analyses also inferred some gene flow between *inornatus*‐N and *superciliaris*‐W (0.09 and 0.13), as well as between *inornatus*‐S and *superciliaris*‐W (0.06 and 0.11).

**FIGURE 10:**
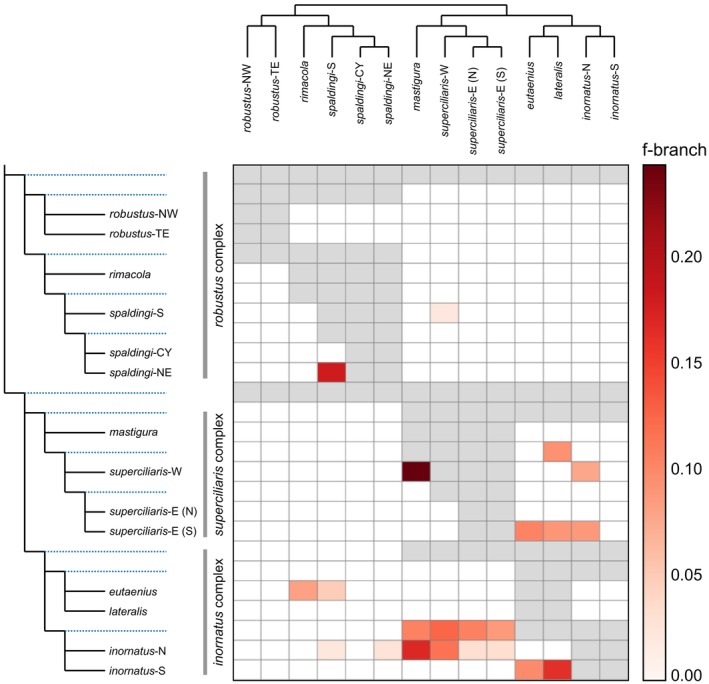
Excess allele sharing among operational taxonomic units (OTUs) in the *Ctenotus inornatus* species group based on f‐branch, an ABBA‐BABA‐class statistic. Colours indicate the proportion of an OTU's loci inferred to be introgressed from another OTU. Grey squares indicate comparisons not possible owing to tree topology constraints. The f‐branch metric accounts for correlated allele frequencies among species to minimize cases where multiple species appear to be introgressed owing to a single introgression event that involved an ancestor (a potential limitation of the broadly used Patterson's D; see Figure S8 for D results). Such ancestral events are indicated by the internal branches on the y‐axis (blue dotted lines). Note substantial introgression across OTUs from distinct complexes, particularly *inornatus* and *superciliaris*, but also among non‐sister OTUs within the three complexes. [Colour figure can be viewed at wileyonlinelibrary.com]

In agreement with these results, ABBA‐BABA allele patterns support substantial nuclear introgression between OTUs from distinct complexes (Figure 10). The proportion of loci inferred to have experienced introgression across delimited species were often high, up to 15%–25%. This was the case for *inornatus‐*N and each of *superciliaris*‐E, *superciliaris*‐W, and *C. mastigura*. Estimates of Patterson's D, a metric related to f‐branch, were broadly consistent with these results (Figure S8). ABBA‐BABA patterns also suggest that admixture events might have involved the ancestors of living species, as revealed by f‐branch estimates corresponding to internal branches of the tree (Figure 10). This was the case of the branch connecting *inornatus‐*N and *inornatus‐*S, found to have introgressed with each of the three *superciliaris* complex OTUs. Likewise, the presumed ancestor of *superciliaris*‐E and *superciliaris*‐W was inferred to have introgressed with both *inornatus*‐S and *C. lateralis*. These introgressed OTUs often showed high levels of cytonuclear discordance (Figure 5; Figure S6).

## 4 DISCUSSION

This study inferred population structure and history based on broad geographic and genetic sampling of lizards long considered to be challenging taxonomically. Beyond attempting to delimit species, we sought to identify the evolutionary processes that might underlie ambiguous genotypic and phenotypic species boundaries. Our results revealed a perfect storm of rampant taxon paraphyly, both morphologically cryptic and polytypic lineages, and a wide range of gene flow levels between candidate species. In addition, we found evidence of mitochondrial capture and heterogeneous patterns of nuclear introgression across divergent OTUs. Mutually introgressed OTUs have partially overlapping ranges, particularly in northern Australia. These findings suggest that taxonomic uncertainty may result from spatial variation in the porosity of species boundaries and the resulting geographic patterns of genetic and phenotypic variation. Below, we discuss the implications of our findings to inferences of speciation and the systematics of challenging clades.

### 4.1 Consequences of widespread admixture for inference of species boundaries

This study demonstrates that extensive sampling might be necessary to uncover instances of geographically widespread genetic admixture (Oliver et al., 2020; Singhal et al., 2018), which might otherwise lead to overestimation of population separation. In particular, IBD analyses support genetic intergradation across localities corresponding to *inornatus*‐N and *inornatus*‐S (Figure 6). This pattern suggests that these two OTUs comprise a genetic and geographic continuum, consistent with substantial gene flow estimated between them (Figure 7). Still, these two OTUs formed coherent and distinctive lineages and genotypic clusters (Figure 9), thereby conforming to species criteria that have been widely applied (Cracraft, 1987; de Queiroz, 1998; Mallet, 2013, 2020). This scenario supports that certain approaches can infer population separation, often interpreted as evidence of multiple species, in the presence of spatially extensive gene flow, in line with theoretical and empirical investigations (Barley et al., 2018; Battey et al., 2020; Bradburd et al., 2018; Irwin, 2002). Such continuous genetic differentiation frequently coincides with clinal phenotypic differentiation, further contributing to blurred species limits (e.g., Ahossou et al., 2020; Chambers & Hillis, 2020; Dickens et al., 2021; Pereira & Wake, 2009). In the case of *inornatus*‐N and *inornatus*‐S, denser sampling could lead to increased resolution of genotypic transitions in geographic space. More broadly, our findings illustrate how characterizing the geographic extent of such transitions is crucial to avoid imposing discrete taxonomic structures on continuous patterns of variation (Braby et al., 2012; Chambers & Hillis, 2020; Mayr, 1963; Prates, Doughty, & Rabosky, 2022; Wilson & Brown, 1953).

### 4.2 Phenotypic conservatism conceals evolutionary separation

This study supports the idea that poor taxonomic resolution can also result from species formation with little to no morphological change (Bickford et al., 2007; Camp & Wooten, 2016; Fišer et al., 2018). Unlike the *inornatus* complex, IBD does not explain the differentiation of OTUs in the *robustus* and *superciliaris* complexes. In these cases, genetic distances are primarily decoupled from geographic distances (Figure 6), matching expectations of evolutionary separation (Figure 1d). Accordingly, we inferred low gene flow between OTUs in both complexes (Figure 7). This scenario suggests that multiple morphologically cryptic species occur within single nominal taxa, as observed here within each of *C. robustus*, *C. spaldingi*, and *C. superciliaris*. One of these candidate species, *robustus*‐TE, is sympatric with its sister, *robustus*‐NW, passing what is typically considered the strongest test for evolutionary separation: the maintenance of distinct gene pools in sympatry (Mayr, 1963). Speciation without morphological change can result from stabilizing selection, developmental constraints, or neutral divergence between populations in similar, potentially constant environments (Zamudio et al., 2016). Moreover, diverging traits across species can be cryptic to humans, as is the case of chemical signals or ecophysiological tolerances (Cadena & Zapata, 2021; Zozaya et al., 2019). Future morphological examinations informed by the genetic patterns might help identify characters that better reflect evolutionary divergence (e.g., Prates, Hutchinson, et al., 2022; Teixeira et al., 2016).

### 4.3 Genetic introgression contributes to fuzzy species boundaries

Beyond IBD and cryptic divergence, we find that several unclear species boundaries in this lizard clade may result from genetic introgression. Our finding of conflicting genealogies between mitochondrial and nuclear DNA might originate from stochastic allele sorting among lineages (Firneno et al., 2020; Singhal & Moritz, 2012). However, it seems unlikely that coherent nuclear units would retain unsorted mitochondrial alleles given the shorter coalescent times of mitochondrial relative to nuclear DNA (Palumbi et al., 2001). Alternatively, mitonuclear discordances can arise from mitochondrial capture through hybridization, as reported in many organisms (e.g., Currat et al., 2008; Good et al., 2008; Irwin et al., 2009). This process might explain the mitochondrial DNA paraphyly of several coherent nuclear DNA‐based OTUs, such as *inornatus*‐N, *inornatus*‐S, *superciliaris*‐E, and *superciliaris*‐W. This propensity of mitochondrial genomes to introgress across species boundaries can limit their utility in species delimitation (Funk & Omland, 2003). To corroborate mitochondrial evidence of evolutionary separation (or lack thereof), investigators will continue to benefit from integrative analyses of morphological characters, behavioural traits, and multi‐locus datasets (Cadena & Zapata, 2021; Padial et al., 2010; Schlick‐Steiner et al., 2010).

In agreement with the mitochondrial patterns, analyses of nuclear loci support that excess allele sharing among species in the *C. inornatus* species group results from introgression. Remarkably, introgression levels appear largely decoupled from phylogenetic relatedness. ABBA‐BABA analyses found that some of the highest levels of allele sharing occur among OTUs from distinct complexes (Figure 10). Likewise, historical demographic modelling inferred higher gene flow between distantly related sympatric OTUs than between certain closely related OTUs (Figure 7). This decoupling between introgression levels and relatedness differs from patterns reported in other clades (Barley et al., 2022; Hamlin et al., 2020; Peñalba et al., 2019; Roux et al., 2016; Singhal & Bi, 2017; Winger, 2017). The proportions of loci inferred as admixed in the *C. inornatus* group are similar to those of organisms thought to hybridize extensively, such as the African cichlids and true toads (Malinsky et al., 2018; Rivera et al., 2021; but see Dagilis et al., 2022). As in *Ctenotus* lizards, species delimitation in these groups is challenging. Hybridization has long been attributed to blurred genotypic and phenotypic boundaries in plants (Lotsy, 1925; McVay et al., 2017; Novaković et al., 2022; Robinson et al., 2001; Shaw & Small, 2004), but our study contributes to a growing body of evidence that hybridization is also a source of taxonomic uncertainty in animal clades (Gill, 2014; Pyron et al., 2020).

Introgressive hybridization increases the variance in genealogical topologies and coalescent times across genome regions, confounding molecular species delimitation. On one hand, gene tree paraphyly owing to horizontal transfer can lead to spurious lumping of divergent lineages (Gill, 2014). On the other hand, emergent allele combinations in admixed populations can lead to spurious inference of separate lineages and genotypic clusters (Chan et al., 2020, 2021). Additionally, spatial phenotypic mosaics resulting from secondary contact can be interpreted as polytypism within a single species (O'Connell et al., 2021). In the *C. inornatus* species group, introgression might have contributed to phenotypic parallelisms that obscure species boundaries. Specimens corresponding to *inornatus*‐S and *superciliaris‐*E (S) appear to share the well‐marked “*saxatilis*” coloration pattern in the central deserts; *inornatus*‐N and *superciliaris‐*E (N) share the subdued “*inornatus*” pattern in the Top End; and *superciliaris‐*W shares both of these phenotypes with *inornatus* complex lineages along the northwestern coast (Rabosky et al., 2014). Trait sharing among species might result from introgression in the genes that underlie such traits (Smith & Kronforst, 2013; Svardal et al., 2020). However, this pattern can also result from retained ancestral polymorphisms or parallel or convergent evolution (Mims et al., 2010; Muir & Schlötterer, 2005; Zamudio et al., 2016; but see Edelman et al., 2019). While the mechanisms underlying phenotypic parallelisms in *Ctenotus* are unknown, our results confirm that certain characters broadly used to define scincid lizard taxa, such as coloration, do not always reflect evolutionary divergence (e.g., Prates, Doughty, & Rabosky, 2022; Rabosky et al., 2014; Rivera et al., 2020).

Lastly, our findings raise questions about the maintenance of species limits in this group. For instance, migration rates between some combinations of sympatric species appear to be higher than those inferred between parapatric species (Figure 7). This is the case, for instance, of the sympatric pair *inornatus*‐N and putative *C. mastigura*, whose gene flow estimates, albeit low, were higher than those between the closely related and parapatric *C. mastigura*, *superciliaris*‐E (N), and superciliaris‐E (S). How do species remain cohesive in the face of opportunities for hybridization? One possibility is that introgression from another species is restricted to a small subset of a species' range or only part of their divergence history. Alternatively, specific barrier loci might remain differentiated despite broad admixture across the genome (Baird, 1995; Barton, 1983; Harrison & Larson, 2014). Another emerging question is why mitochondrial and nuclear introgression in the *C. inornatus* group appear concentrated in northern Australia. Studies of animals and plants have found introgressive hybridization in regions of high climatic dynamism in the Quaternary (Dufresnes et al., 2020; Folk et al., 2018; Shu et al., 2022), including the northern Australian monsoonal‐arid zone interface and wet tropics (e.g., Catullo & Keogh, 2014; Hoskin et al., 2011; Laver et al., 2018; Singhal & Moritz, 2012). Climate‐driven habitat changes may lead to introgression by promoting species range shifts and secondary contact (Currat et al., 2008; Cutter & Gray, 2016; Folk et al., 2018; Garroway et al., 2010; Prates et al., 2018). However, other groups from the monsoonal tropics show deep phylogeographic structure, consistent with a history of range stability (e.g., Bowman et al., 2010; Moritz et al., 2016; Potter et al., 2018; Rosauer et al., 2018), while some analyses have inferred dynamism also in the arid zone (e.g., Byrne et al., 2008; Pepper & Keogh, 2021; Prideaux et al., 2007). We still have a limited understanding of the historical processes that have structured the genomes of *Ctenotus* lizards. Nevertheless, clades with elusive species boundaries, like the *C. inornatus* species group, emerge as promising candidates for investigating the environmental drivers and evolutionary consequences of introgressive hybridization.

### 4.4 Taxonomic recommendations and outstanding issues

Systematic uncertainty persists in our focal lizards, but our results provide clarity on several issues. We found continuous spatial genetic differentiation between *inornatus* complex populations from northern Australia (corresponding to *inornatus*‐N) and the central arid zone (*inornatus*‐S). Some population genetic structure between these regions appears to align with a physiographic transition from sandy desert to stony uplands and monsoonal woodlands in northern Australia. Still, gene flow across this transition seems high enough to generate a pattern consistent with IBD over the range of a single species. Populations corresponding to *inornatus*‐N and *inornatus*‐S have been assigned to different taxa (Storr, 1969), which nonetheless cannot be reliably identified based on current perceptions of their morphological variation (Rabosky et al., 2014). Future morphological examinations informed by the genetic patterns might help clarify whether some of the inferred OTUs are truly cryptic. For the time being, we recommend treating the northern and southern populations as a single taxon, with the name *Ctenotus inornatus* (Gray, 1845) retaining priority to refer to both.

We found that some traditionally recognized taxa have limited coherence or divergence. For instance, specimens assigned to “*C. saxatilis*” appear to correspond to multiple *inornatus* and *superciliaris* complex lineages. Likewise, *C*. “*borealis*” and *C*. “*severus*” grouped respectively with *C. robustus* and *C. inornatus*, with which they experience high gene flow. These findings corroborate phylogenetic patterns inferred based on fewer loci (Rabosky et al., 2014). The case of *C*. “*fallens*” and *C*. “*brachyonyx*” is less clear. Populations corresponding to these taxa were genetically clustered within other taxa (*C*. “*helenae*”, *C*. “*saxatilis*”), yet separated from other samples by long branches. Relatively fewer alleles migrate between *C*. “*fallens*” or *C*. “*brachyonyx*” and geographically adjacent populations, but these numbers appear to reflect lower effective population sizes rather than lower proportions of migrants. For instance, their N estimates were up to 20 times lower than those of other populations (Table S3). Such low N contributed to low 2NM for *C*. “*fallens*” and *C*. “*brachyonyx*” despite high estimates of M, that is, high proportions of individuals originating from other populations (Table S3). This scenario suggests that 2NM, a composite metric designed to express absolute numbers of gene migrations (Pinho & Hey, 2010), might conceal high relative gene flow when population sizes are small. Overall, our findings suggest that several traditionally recognized taxa correspond to regional forms within wide‐ranging polytypic species, but broader sampling may be needed to resolve specific cases.

Our analyses also revealed OTUs that might correspond to undescribed species. OTUs *robustus*‐NW and *robustus*‐TE, both corresponding to the taxon *C. robustus*, are sympatric but appear genetically isolated. Similarly, gene flow approached zero between *spaldingi*‐CY and *spaldingi*‐NE, both referred to *C. spaldingi*. Finally, *superciliaris*‐E (N), *superciliaris*‐E (S), and *superciliaris*‐W, all within *C. superciliaris*, appear divergent despite their potential geographic overlap. Further sampling in putative contact zones may be necessary to establish the degree of separation between these populations. From a nomenclatural perspective, it is also important to determine whether these OTUs correspond to valid yet poorly characterized taxa. For instance, *spaldingi*‐CY or *spaldingi*‐S might correspond to the eastern taxa *C. capricorni* Storr, 1981 or *C. nullum* Ingram & Czechura, 1990. Alternatively, they might represent names currently under synonymy, such as *C. harringtonensis* (Wells & Wellington, 1985), *C. josephinae* (Wells & Wellington, 1984), or *Ctenotus dorsale* (Boulenger, 1887). Lack of genetic data unambiguously corresponding to these taxa prevents testing whether they are present in our sample. Given the lack of DNA sequences for most name‐bearing type specimens, assigning names to lineages typically relies on morphological comparisons. It remains to be seen whether the emerging genetic patterns will help identify morphological features that confidently assign unsequenced specimens to lineages.

This investigation supports that resolving species boundaries in the *C. inornatus* species group and other “taxonomic disaster zones” will require developing a broad understanding of population history, spatial genetic variation, patterns of genomic introgression, and their effects on phenotypic variation. This comprehensive understanding may, in turn, require renewed field‐based collecting and specimen vouchering in undersampled geographic regions. These requirements illustrate the formidable challenges and opportunities brought by the increasing conceptual and operational unification of taxonomic practice and speciation biology.

## AUTHOR CONTRIBUTIONS

IP, DLR, and MNH conceived and designed the study. DLR, SS, and CM acquired funding. DLR, MNH, and CM provided samples and resources. IP wrote computer scripts, processed the data, and prepared the visualization of results. IP, DLR, MNH, SS, and CM interpreted the results. IP and DLR drafted the manuscript with suggestions and edits by MNH, SS, and CM.

## Supporting information


Data S1.

## Data Availability

Nuclear ddRAD data are available in the Sequence Read Archive (BioProjects PRJNA755251 and PRJNA382545); see Table S1 for ddRAD sample information. Newly generated mitochondrial data were uploaded to GenBank (accession numbers OQ091785–OQ091921); see Table S2 for the accession numbers of mitochondrial sequences, including those generated by previous studies. Computer scripts used to prepare the data and perform all analyses are available through GitHub (https://github.com/ivanprates/Ctenotus_inornatus_group). A copy of the supplementary material is available through GiHub.
