# Supplementary material for: Notes from the taxonomic disaster zone: Evolutionary drivers of intractable species boundaries in an Australian lizard clade (Scincidae: Ctenotus)
Source: Mol Ecol. 2023 Jul 17;33(20):e17074. doi: 10.1111/mec.17074 (PMC13084965; doi:10.1111/mec.17074)
Supplement: Supplementary file 1 — Data S1. [file MEC-33-e17074-s001.pdf]

SUPPLEMENTARY INFORMATION

Notes from the taxonomic disaster zone: Evolutionary drivers of intractable species boundaries in an Australian lizard clade (Scincidae: *Ctenotus*)

Ivan Prates, Mark N. Hutchinson, Sonal Singhal, Craig Moritz, Daniel L. Rabosky

Molecular Ecology

This document contains:

Supplementary Figures S1 to S8.

Supplementary Tables S1 to S3.

Figure S1. Geographic distribution of mitochondrial samples partitioned by the corresponding nuclear operational taxonomic units.

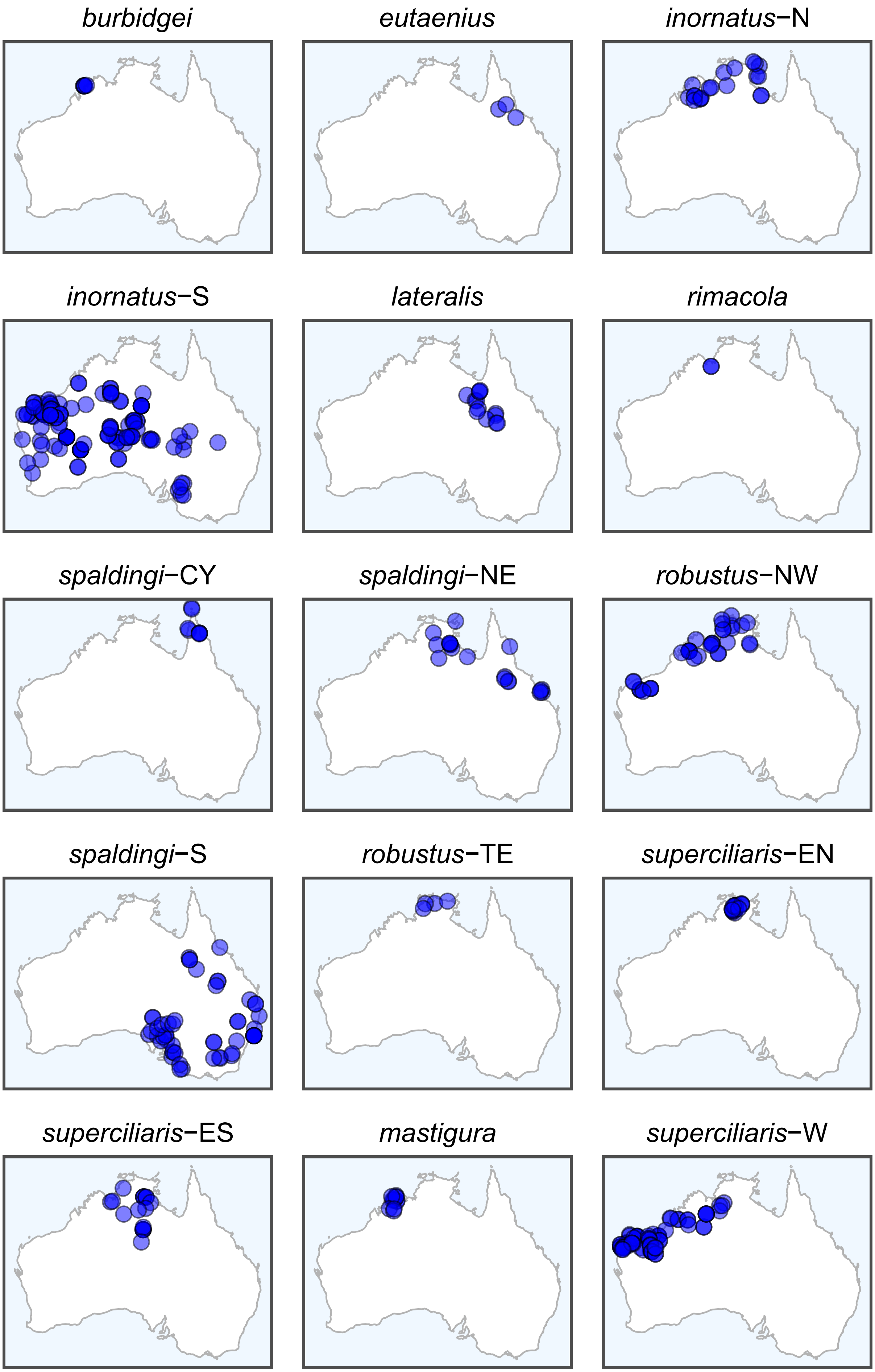

Figure S2. Geographic distribution of mitochondrial samples partitioned by the corresponding putative *Ctenotus* taxa.

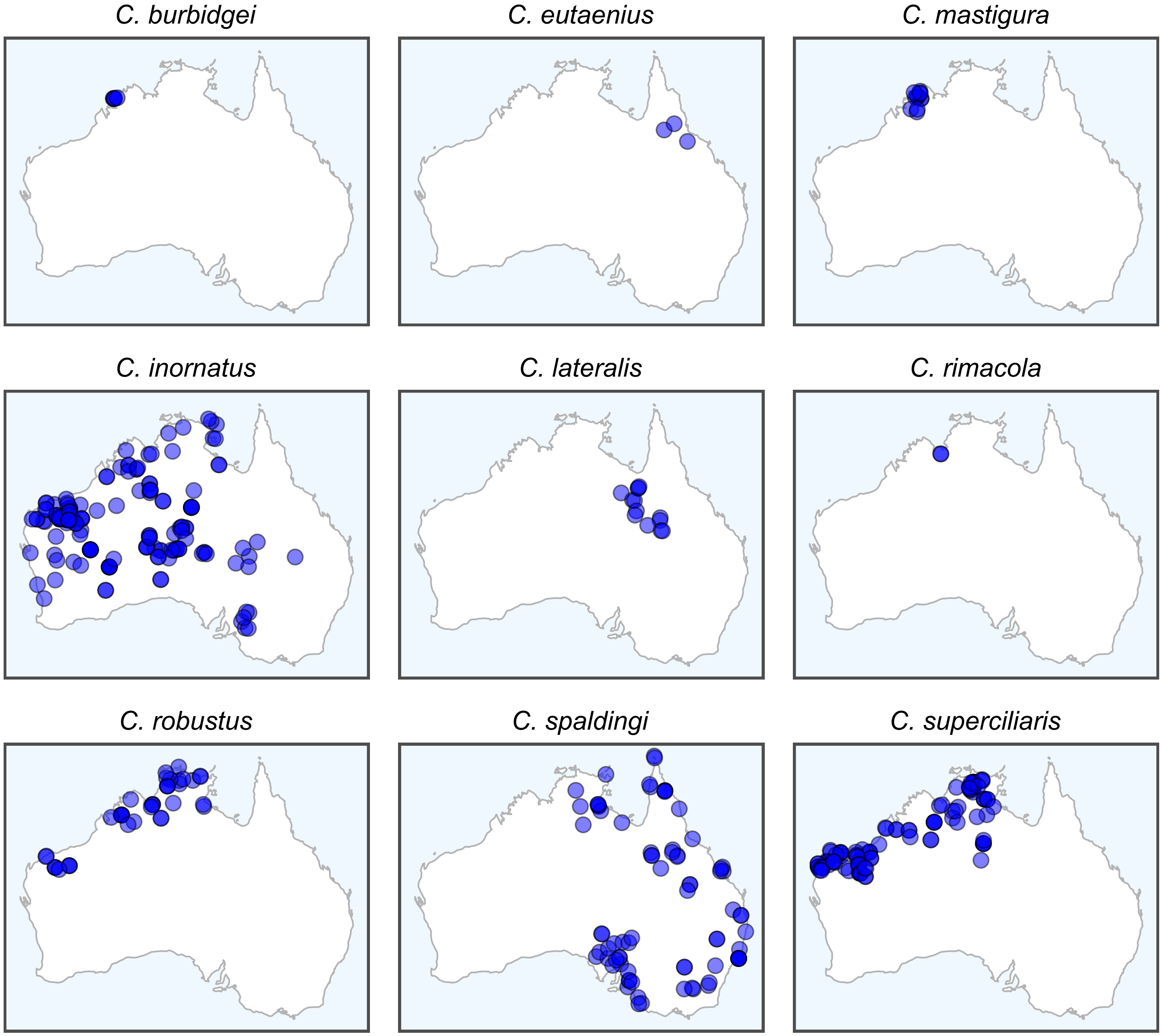

Figure S3. Geographic distribution of groups used in G−PhoCS analyses.

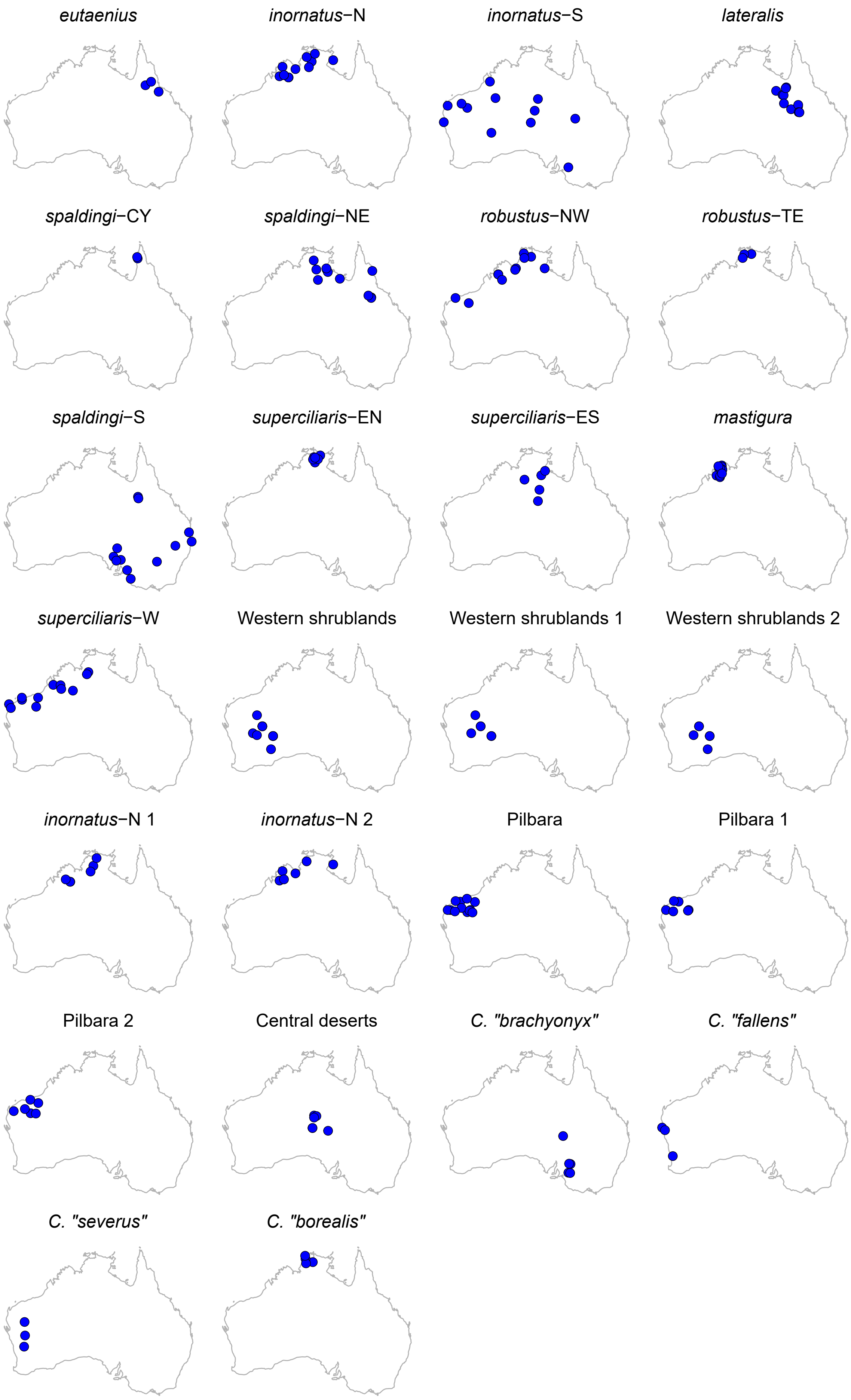

Figure S4. Phylogenetic relationships between nuclear operational taxonomic units in the *Ctenotus inornatus* species group inferred using the SVD Quartets species-tree method.

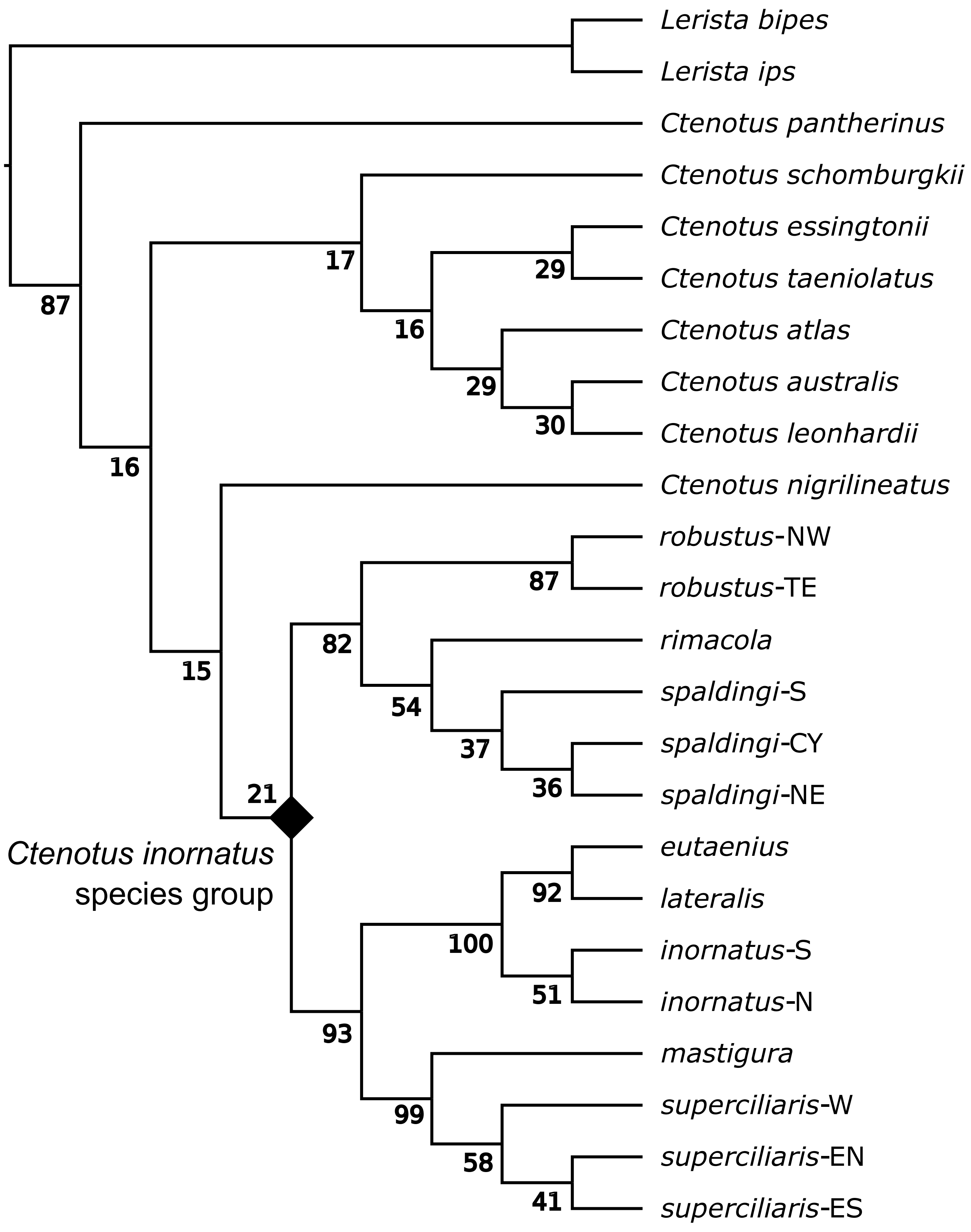

Figure S5. Sample clustering in genotypic space for each of the three complexes in the *Ctenotus inornatus* species group based on a principal component analysis on the SNP data. Colors correspond to the inferred Operational Taxonomic Units.

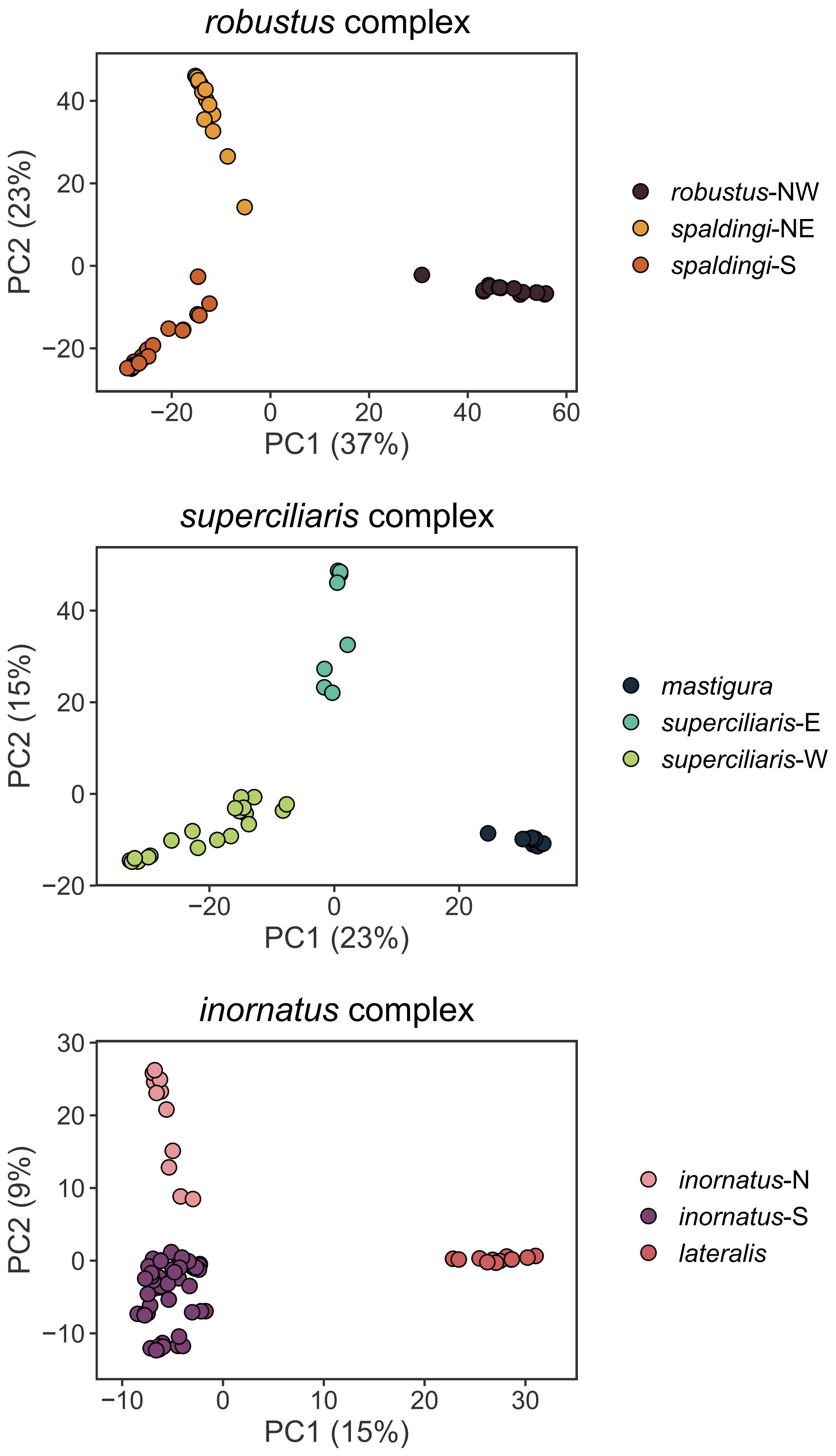

Figure S6. Phylogenetic relationships based on all mitochondrial samples.

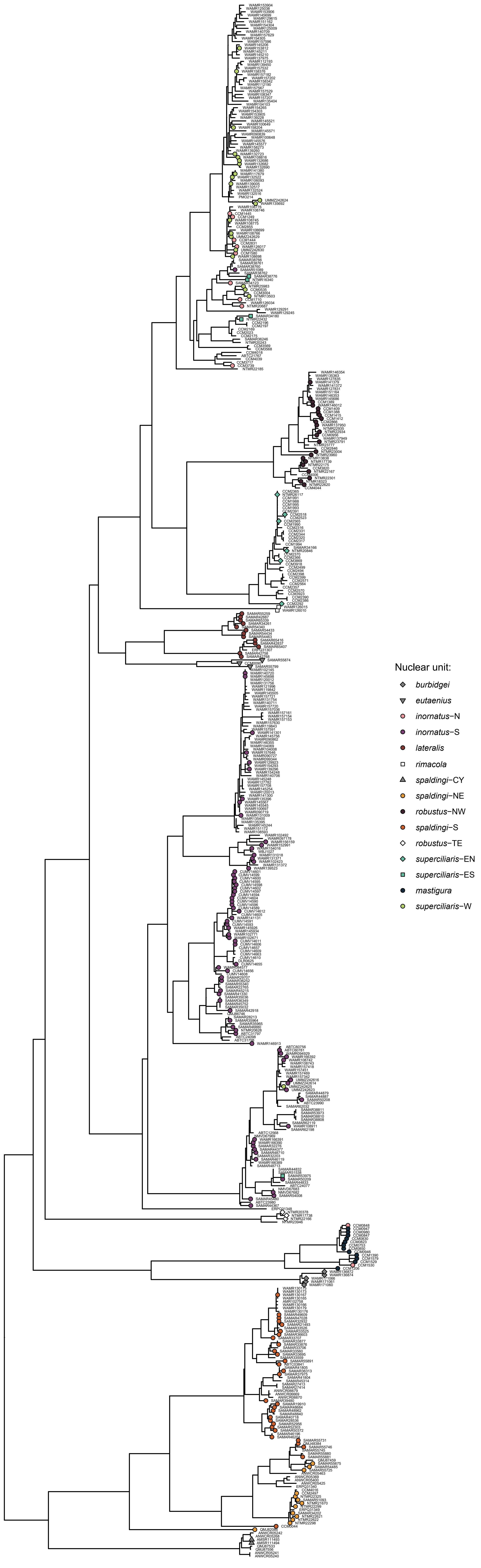

Figure S7. Evidence of two units within *superciliaris*-E based on isolation-by-distance analyses. Plot (left) shows pairwise Fst between individuals from same (gray) or different (black) genetic groups as a function of the geographic distances between them. In this case, *superciliaris*-E is assumed to correspond to a single unit, with northern (N) and interior (S) populations combined. Note the scattered gray points corresponding to the same genetic group in the upper left part of the plot (red arrows), which indicate high genetic divergence despite geographic proximity. This decoupling of geographic and genetic distances supports the action of isolating mechanisms and thus the present of separate species. For details, see text.

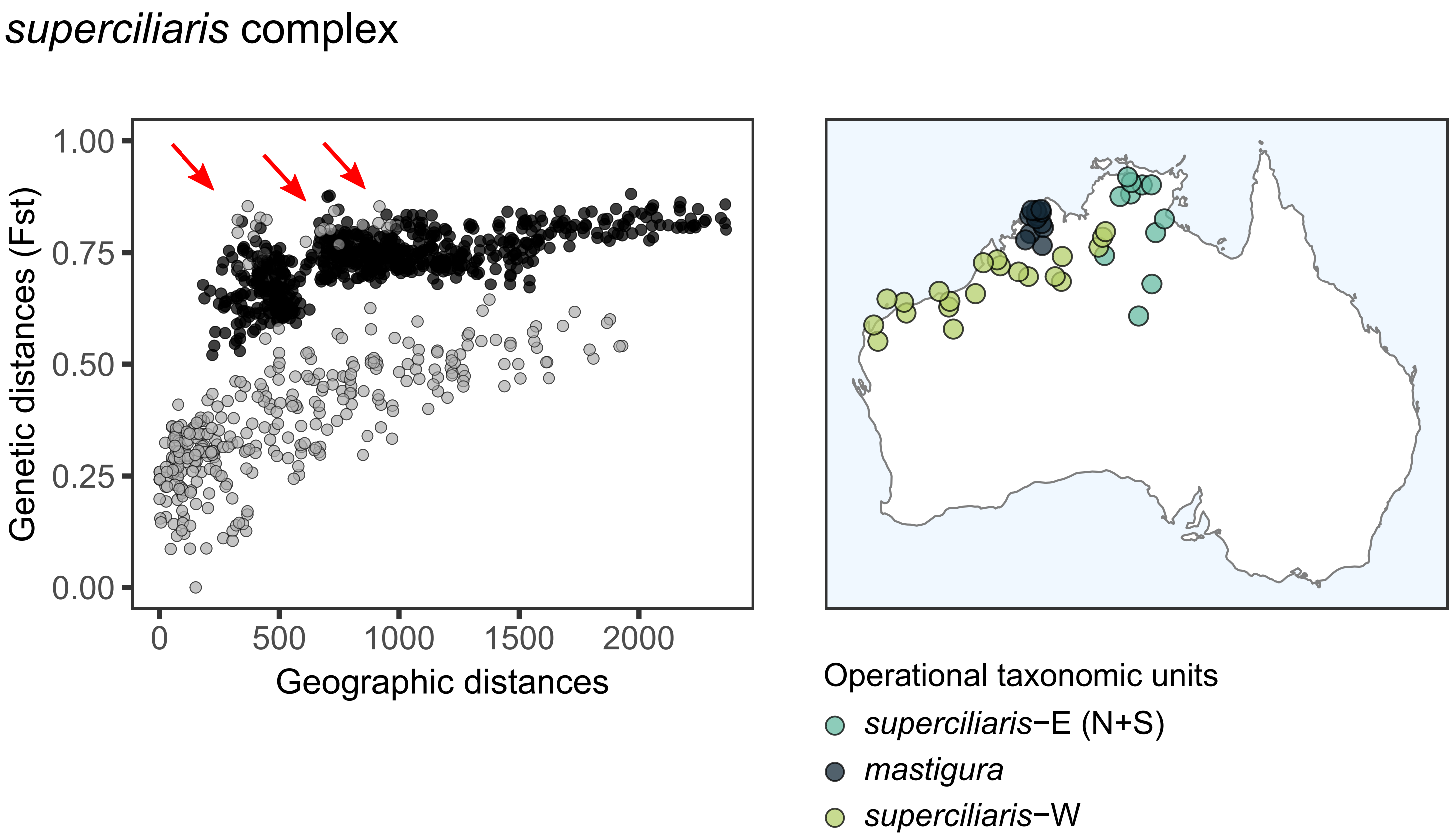

Figure S8. Excess allele sharing among operational taxonomic units based on the D−statistic. Black outlines indicate significant values.

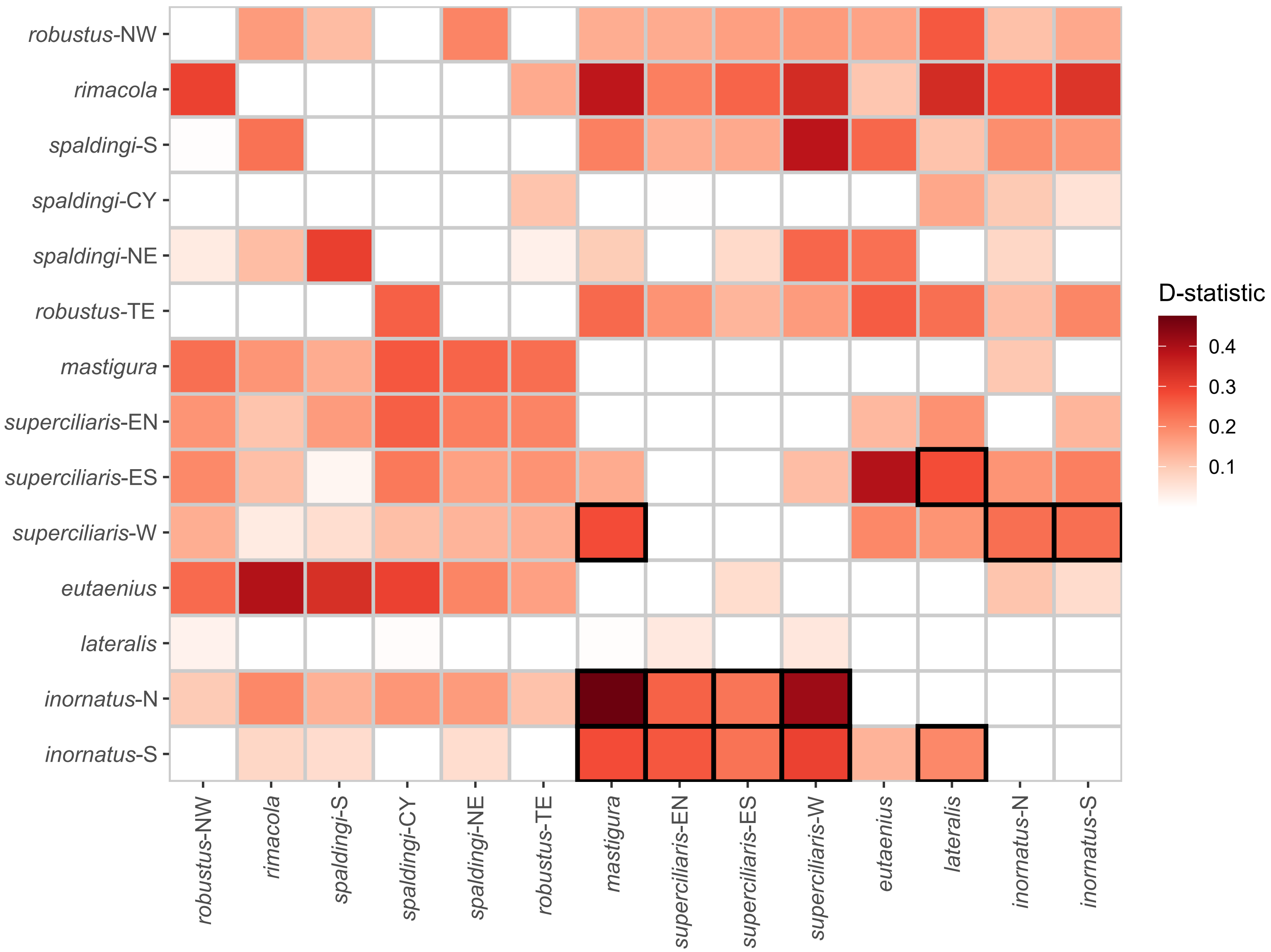

Table S1. ddRAD sample information.

Voucher or Tissue Sample	Sample ID	Tissue ID	Alternative ID	Genus	Original Taxon	Putative Unit	Putative Taxon	State or Territory	Location	Latitude	Longitude	SRA Exp. Accession
ABTC 03841	NA_ABTC03841_Ct_robu	ABTC03841	ROBUA03841	Ctenotus	robustus	spaldingi−S	spaldingi	QLD	14 km W Cooyar	−27.00000	151.8300	SRX2727431
ABTC 31797	NA_ABTC31797_Ct_saxa	ABTC31797	SAXAA31797	Ctenotus	saxatilis	inornatus−S	inornatus	NT	Log Cabin Dam MacDonnell Ranges	−23.80000	133.2000	SRX11811164
ABTC 60781	NA_ABTC60781_Ct_hele	ABTC60781	HELEA60781	Ctenotus	helenae	inornatus−S	inornatus	NT	30 km SW Sangster's Bore	−20.83000	130.4200	SRX2727422
AM R130167	NA_ABTC61569_Ct_robu	ABTC61569	ROBUA61569	Ctenotus	robustus	spaldingi−S	spaldingi	NSW	Smith's Lake	−32.40000	152.5000	SRX11811175
AMS R111493	AMSR_111493_Ct_spal	ABTC11090		Ctenotus	spaldingi	spaldingi−CY	spaldingi	QLD	Watson River	−13.36400	141.7300	SRX2727524
AMS R111494	AMSR_111494_Ct_spal	ABTC11091		Ctenotus	spaldingi	spaldingi−CY	spaldingi	QLD	False Pera Head	−13.08300	141.6160	SRX2727523
ANWC R10126	NA_CCM1710_Ct_inor			Ctenotus	inornatus	inornatus−N	inornatus	NT	Willeroo station tip	−15.29606	131.5851	SRX2727385
CCM 0044	NA_CCM0044_Ct_euta			Ctenotus	eutaenius	spaldingi−S	spaldingi	QLD	NA	−19.13263	146.8690	SRX2727420
CCM 0823	NA_CCM0823_Ct_robu			Ctenotus	robustus	mastigura	cf. mastigura	WA	Carson escarpment TD1	−15.33830	126.5917	SRX2727416
CCM 0830	NA_CCM0830_Ct_inor			Ctenotus	inornatus	mastigura	cf. mastigura	WA	Carson escarpment near camp	−15.33940	126.5893	SRX2727415
CCM 0847	NA_CCM0847_Ct_robu			Ctenotus	robustus	mastigura	cf. mastigura	WA	Old Mitchell Q5	−15.13280	126.1478	SRX2727414
CCM 0848	NA_CCM0848_Ct_inor			Ctenotus	inornatus	inornatus−N	inornatus	WA	Spring Creek SS2	−15.20020	125.9067	SRX2727413
CCM 0849	NA_CCM0849_Ct_inor			Ctenotus	inornatus	mastigura	cf. mastigura	WA	Spring Creek SS2	−15.20020	125.9067	SRX2727412
CCM 0850	NA_CCM0850_Ct_inor			Ctenotus	inornatus	mastigura	cf. mastigura	WA	Spring Creek SS1	−15.19890	125.9035	SRX2727411
CCM 0946	NA_CCM0946_Ct_inor			Ctenotus	inornatus	mastigura	cf. mastigura	WA	King Ed Riv Theda campsite 30 km N of Homestead	−14.51903	126.4581	SRX2727410
CCM 0947	NA_CCM0947_Ct_inor			Ctenotus	inornatus	mastigura	cf. mastigura	WA	Baitbox Hill Theda site 7 10 km N of HS	−14.73119	126.4600	SRX2727409
CCM 0956	NA_CCM0956_Ct_robu			Ctenotus	robustus	robustus−NW	robustus	WA	Baitbox Hill Theda site 2 10 km N of HS	−14.75187	126.4772	SRX2727408
CCM 1409	NA_CCM1409_Ct_robu			Ctenotus	robustus	robustus−NW	robustus	WA	Potts Black soil	−16.46680	125.3723	SRX2727397
CCM 1412	NA_CCM1412_Ct_robu			Ctenotus	robustus	robustus−NW	robustus	WA	Potts riparian	−16.49400	125.3417	SRX2727396
CCM 1415	NA_CCM1415_Ct_robu			Ctenotus	robustus	robustus−NW	robustus	WA	Potts riparian campsite	−16.48940	125.3518	SRX2727395
CCM 2292	NA_CCM2292_Ct_inor	CCM2292		Ctenotus	inornatus	superciliaris−E (N)	superciliaris	NT	Kakadu fire plot 27	−13.94278	132.9029	SRX11811102
CCM 2497	NA_CCM2497_Ct_spal	CCM2497		Ctenotus	spaldingi	spaldingi−NE	spaldingi	NT	Kakadu fire plot 133B	−13.74413	132.6244	SRX11811103
CCM 2518	NA_CCM2518_Ct_vert	CCM2518		Ctenotus	vertebralis	superciliaris−E (N)	superciliaris	NT	Kakadu fire plot 26	−13.35180	132.4723	NA
CCM 2565	NA_CCM2565_Ct_vert	CCM2565		Ctenotus	vertebralis	superciliaris−E (N)	superciliaris	NT	Kakadu fire plot 126	−12.99761	132.9327	NA
CCM 2866	NA_CCM2866_Ct_robu	CCM2866		Ctenotus	robustus	robustus−NW	robustus	WA	Bluebush SV08 Mornington	−17.55431	126.1646	SRX11811106
CCM 3739	NA_CCM3739_Ct_inor	CCM3739		Ctenotus	inornatus	inornatus−N	inornatus	NT	Groote Eyland	−13.90577	136.8206	SRX11811107
CCM 3869	NA_CCM3869_Ct_inor	CCM3869		Ctenotus	inornatus	superciliaris−E (N)	superciliaris	NT	Ngangkan	−12.55387	134.0290	SRX11811108
CCM 4920	NA_CCM4920_Ct_essi			Ctenotus	essingtonii	inornatus−N	inornatus	NA	Edith Falls campsite Nitmaluk NP	−14.17983	132.1861	NA
CCM 5477	NA_CCM5477_Ct_vert			Ctenotus	vertebralis	spaldingi−NE	spaldingi	NA	Site 14 elliott trap Nitmiluk NP	−14.03203	132.3069	NA
CUMV 14589	CUMV_14589_Ct_hele	DLR0036		Ctenotus	helenae	inornatus−S	inornatus	WA	Lorna Glen Stn	−26.22600	121.5575	SRX2727463
CUMV 14590	CUMV_14590_Ct_hele	DLR0119		Ctenotus	helenae	inornatus−S	inornatus	WA	Lorna Glen Stn	−26.22600	121.5575	SRX2727462

Voucher or Tissue Sample	Sample ID	Tissue ID	Alternative ID	Genus	Original Taxon	Putative Unit	Putative Taxon	State or Territory	Location	Latitude	Longitude	SRA Exp. Accession
CUMV 14591	CUMV_14591_Ct_hele	DLR0134		Ctenotus	helenae	inornatus−S	inornatus	WA	Lorna Glen Stn	−26.22600	121.5575	SRX2727461
CUMV 14593	CUMV_14593_Ct_hele	DLR0093		Ctenotus	helenae	inornatus−S	inornatus	WA	Lorna Glen Stn	−26.22600	121.5575	SRX2727460
CUMV 14594	CUMV_14594_Ct_hele	DLR0116		Ctenotus	helenae	inornatus−S	inornatus	WA	Lorna Glen Stn	−26.22600	121.5575	SRX2727459
CUMV 14595	CUMV_14595_Ct_hele	DLR0132		Ctenotus	helenae	inornatus−S	inornatus	WA	Lorna Glen Stn	−26.22600	121.5575	SRX2727458
CUMV 14596	CUMV_14596_Ct_hele	DLR0179		Ctenotus	helenae	inornatus−S	inornatus	WA	Lorna Glen Stn	−26.22600	121.5575	SRX2727457
CUMV 14597	CUMV_14597_Ct_hele	DLR0070		Ctenotus	helenae	inornatus−S	inornatus	WA	Lorna Glen Stn	−26.22600	121.5575	SRX2727456
CUMV 14598	CUMV_14598_Ct_hele	DLR0090		Ctenotus	helenae	inornatus−S	inornatus	WA	Lorna Glen Stn	−26.22600	121.5575	SRX2727455
CUMV 14599	CUMV_14599_Ct_hele	DLR0130		Ctenotus	helenae	inornatus−S	inornatus	WA	Lorna Glen Stn	−26.22600	121.5575	SRX2727454
CUMV 14600	CUMV_14600_Ct_hele	DLR0049	HELEDLR0049	Ctenotus	helenae	inornatus−S	inornatus	WA	Lorna Glen Stn	−26.22600	121.5575	SRX2727453
CUMV 14601	CUMV_14601_Ct_hele	DLR0141		Ctenotus	helenae	inornatus−S	inornatus	WA	Lorna Glen Stn	−26.22600	121.5575	SRX2727452
CUMV 14602	CUMV_14602_Ct_hele	DLR0193	HELEDLR0193	Ctenotus	helenae	inornatus−S	inornatus	WA	Lorna Glen Stn	−26.22600	121.5575	SRX2727451
CUMV 14604	CUMV_14604_Ct_hele	DLR0223	HELEDLR0223	Ctenotus	helenae	inornatus−S	inornatus	WA	Lorna Glen Stn	−26.22600	121.5575	SRX2727450
CUMV 14606	CUMV_14606_Ct_hele	DLR0563	HELEDLR0563	Ctenotus	helenae	inornatus−S	inornatus	WA	Yamarna Stn	−28.13300	123.8667	SRX2727449
CUMV 14611	CUMV_14611_Ct_hele	DLR0278	HELEDLR0278	Ctenotus	helenae	inornatus−S	inornatus	WA	Yamarna Stn	−28.13300	123.8667	SRX2727448
CUMV 14612	CUMV_14612_Ct_hele	DLR0376	HELEDLR0376	Ctenotus	helenae	inornatus−S	inornatus	WA	Lake Mason Stn	−27.58600	119.5208	SRX11811131
CUMV 14655	CUMV_14655_Ct_hele	DLR0592		Ctenotus	helenae	inornatus−S	inornatus	WA	Yamarna Stn	−28.13300	123.8667	SRX2727447
CUMV 14656	CUMV_14656_Ct_hele	DLR0605		Ctenotus	helenae	inornatus−S	inornatus	WA	Yamarna Stn	−28.13300	123.8667	SRX2727446
CUMV 14681	CUMV_14681_Ct_scho	DLR0032	SCHODLR0032	Ctenotus	schomburgkii	schomburgkii	schomburgkii	WA	Lorna Glen Stn	−26.22600	121.5575	SRX2727445
CUMV 14700	CUMV_14700_Ct_scho	DLR0370	SCHODLR0370	Ctenotus	schomburgkii	schomburgkii	schomburgkii	WA	Lake Mason Stn	−27.58600	119.5208	SRX2727443
NMVD 67682	NMVD_67682_Ct_saxa	ABTC09987	SAXAA09987	Ctenotus	saxatilis	inornatus−S	inornatus	NT	Ormiston	−23.70000	132.7000	SRX2727380
NMVD 67793	NMVD_67793_Ct_leae	ABTC10009		Ctenotus	leae	inornatus−S	inornatus	NT	Finke River	−24.04250	132.7060	SRX2727379
NTM R13503	NTMR_13503_Ct_saxa	ABTC28062	SAXAA28062	Ctenotus	saxatilis	superciliaris−W	superciliaris	NT	Victoria R Gregory NP	−16.82300	130.4200	SRX2727378
NTM R13838	NTMR_13838_Ct_bore	ABTC28390	BOREA28390	Ctenotus	borealis	robustus−NW	robustus	NT	Kakadu NP	−13.03000	132.4300	SRX2727375
NTM R16340	NTMR_16340_Ct_inor	ABTC28499	INORA28499	Ctenotus	inornatus	superciliaris−E (S)	superciliaris	NT	Wave Hill Stn	−17.30000	131.0000	SRX2727372
NTM R17738	NTMR_17738_Ct_cogg	ABTC29190	COGGA29190	Ctenotus	coggeri	robustus−TE	robustus	NT	Jabiluka Project Area	−12.50000	132.8500	SRX2727371
NTM R17739	NTMR_17739_Ct_bore	ABTC29191		Ctenotus	borealis	robustus−NW	robustus	NT	Casuarina Coastal Reserve	−12.37000	130.8500	SRX2727370
NTM R18323	NTMR_18323_Ct_robu	ABTC30302		Ctenotus	robustus	robustus−NW	robustus	NT	Willeroo	−15.16083	131.6692	SRX2727368
NTM R20378	NTMR_20378_Ct_robu	ABTC29172	ROBUA29172	Ctenotus	robustus	robustus−TE	robustus	NT	Fogg Dam	−12.55000	131.3000	SRX11811111
NTM R20628	NTMR_20628_Ct_saxa	ABTC29398	SAXAA29398	Ctenotus	saxatilis	inornatus−S	inornatus	NT	Finke Gorge NP	−24.10000	132.7000	SRX2727366
NTM R20687	NTMR_20687_Ct_robu	ABTC28421	ROBUA28421	Ctenotus	inornatus	inornatus−N	inornatus	NT	Mt Bundy Stn	−13.30000	131.1000	SRX2727365
NTM R20846	NTMR_20846_Ct_saxa	ABTC28173	SAXAA28173	Ctenotus	saxatilis	superciliaris−E (N)	superciliaris	NT	Kakadu	−12.90000	132.6700	SRX2727364

Voucher or Tissue Sample	Sample ID	Tissue ID	Alternative ID	Genus	Original Taxon	Putative Unit	Putative Taxon	State or Territory	Location	Latitude	Longitude	SRA Exp. Accession
NTM R21670	NTMR_21670_Ct_spal	ABTC29600		Ctenotus	spaldingi	spaldingi−NE	spaldingi	NT	10 km N Larrimah	−15.53300	133.1830	SRX2727361
NTM R22166	NTMR_22166_Ct_robu	ABTC29697		Ctenotus	robustus	robustus−TE	robustus	NT	Litchfield NP	−13.26472	130.9633	SRX2727360
NTM R22167	NTMR_22167_Ct_bore	ABTC29698	BOREA29698	Ctenotus	borealis	robustus−NW	robustus	NT	Litchfield NP	−13.26500	130.9622	SRX2727359
NTM R22175	NTMR_22175_Ct_robu	ABTC29704	ROBUA29704	Ctenotus	robustus	robustus−NW	robustus	NT	Litchfield NP	−13.26500	130.9614	SRX2727358
NTM R22188	NTMR_22188_Ct_essi	ABTC29724		Ctenotus	essingtonii	essingtonii	essingtonii	NT	Litchfield NP	−13.41361	130.8972	SRX2727357
NTM R22191	NTMR_22191_Ct_essi	ABTC29726	ESSIA29726	Ctenotus	essingtonii	essingtonii	essingtonii	NT	Litchfield NP	−13.41400	130.8972	SRX2727356
NTM R22298	NTMR_22298_Ct_spal	ABTC30415		Ctenotus	spaldingi	spaldingi−NE	spaldingi	NT	Limmen Gate NP	−15.48333	135.4122	SRX2727355
NTM R22299	NTMR_22299_Ct_spal	ABTC30414		Ctenotus	spaldingi	spaldingi−NE	spaldingi	NT	7 km N Nathan River Station Limmen Gate NP	−15.53306	135.4081	SRX2727354
NTM R22301	NTMR_22301_Ct_robu	ABTC30416	ROBUA30416	Ctenotus	robustus	robustus−NW	robustus	NT	7 km N Nathan R Stn Limmen Gate NP	−15.53300	135.4081	SRX2727353
NTM R22325	NTMR_22325_Ct_spal	ABTC30410		Ctenotus	spaldingi	spaldingi−NE	spaldingi	NT	Tawallah Creek Limmen Gate NP	−16.01583	135.6669	SRX2727352
NTM R22432	NTMR_22432_Ct_saxa	ABTC30404	SAXAA30404	Ctenotus	saxatilis	superciliaris−E (S)	superciliaris	NT	Nathan River Stn Limmen Gate NP	−15.57800	135.4297	SRX2727350
NTM R22620	NTMR_22620_Ct_robu	ABTC29870		Ctenotus	robustus	robustus−NW	robustus	NT	Long Billabong Roper River	−15.30667	135.3408	SRX2727348
NTM R22621	NTMR_22621_Ct_spal	ABTC29871		Ctenotus	spaldingi	spaldingi−NE	spaldingi	NT	Long Billabong Roper River	−15.30667	135.3408	SRX2727347
NTM R22622	NTMR_22622_Ct_spal	ABTC29872		Ctenotus	spaldingi	spaldingi−NE	spaldingi	NT	Long Billabong Roper River	−15.30667	135.3408	SRX2727346
NTM R22934	NTMR_22934_Ct_robu	ABTC30181	ROBUA30181	Ctenotus	robustus	robustus−NW	robustus	NT	Spirit Hills Keep R	−15.30700	129.1583	SRX2727345
NTM R23004	NTMR_23004_Ct_bore	ABTC30072		Ctenotus	borealis	robustus−NW	robustus	NT	creek SW Pickertaramoor	−11.78222	130.7744	SRX2727344
NTM R23791	NTMR_23791_Ct_robu	ABTC30603	ROBUA30603	Ctenotus	robustus	robustus−NW	robustus	NT	Wickham R Gregory NP	−16.85300	130.1856	SRX2727342
NTM R23960	NTMR_23960_Ct_robu	ABTC30705		Ctenotus	robustus	robustus−NW	robustus	NT	Ramingining area Arafura Swamp	−12.21750	134.9847	SRX2727341
NTM R25983	NTMR_25983_Ct_spal	ABTC70692		Ctenotus	spaldingi	superciliaris−W	superciliaris	NT	Jasper Gorge Gregory National Park	−16.03778	130.7900	SRX2727339
NTM R26117	NTMR_26117_Ct_inor	ABTC72530	INORA72530	Ctenotus	inornatus	superciliaris−E (N)	superciliaris	NT	Upper Reaches Arnhemland Plateau	−13.28300	133.5333	SRX2727338
NTM R37354	NA_CCM0535_Ct_inor			Ctenotus	inornatus	superciliaris−W	superciliaris	NT	Vic River Region Escarpment Walk	−15.61091	131.1160	SRX2727418
QM 48384	QM_48384_Ct_robu	ABTC16215	ROBUA16215	Ctenotus	robustus	spaldingi−S	spaldingi	QLD	Winton	−22.40000	143.0000	SRX2727337
QM 82086	QM_82086_Ct_spal	ABTC105811	SPALA105811	Ctenotus	spaldingi	spaldingi−NE	spaldingi	QLD	Lion's Den Pub near Black Mountain	−15.80000	145.2500	SRX2727333
QMJ 90645	NA_ABTC113741_Ct_saxa	ABTC113741		Ctenotus	saxatilis	lateralis	lateralis	QLD	23.5 km NE Alderley Homestead	−22.38500	139.8564	SRX11811142
QMJ 94601	NA_CCM0090_Ct_late	QMA013599		Ctenotus	lateralis	eutaenius	cf. eutaenius	QLD	Cobb Gorge Cobb12	−18.82119	143.4066	SRX2727419
SAMA R19910	SAMR_19910_Ct_robu	ABTC53561	ROBUA53561	Ctenotus	robustus	spaldingi−S	spaldingi	SA	Barber Hill Gawler Rngs	−32.20000	135.1167	SRX2727223
SAMA R21493	SAMR_21493_Ct_robu	ABTC53607	ROBUA53607	Ctenotus	robustus	spaldingi−S	spaldingi	SA	Coorong NP	−35.56700	138.9667	SRX11811148
SAMA R22246	SAMR_22246_Ct_leon	ABTC53612		Ctenotus	leonhardii	leonhardii	leonhardii	SA	Kingoonya	−30.91700	135.3167	SRX11811149
SAMA R28536	SAMR_28536_Ct_robu	ABTC53759	ROBUA53759	Ctenotus	robustus	spaldingi−S	spaldingi	SA	Fresh Wells 67 km NW Iron Knob	−32.40000	136.5333	SRX11811150
SAMA R29707	SAMR_29707_Ct_brac	ABTC53772	BRACA53772	Ctenotus	brachyonyx	inornatus−S	inornatus	SA	Danggali CP	−33.20000	140.9167	SRX2727221
SAMA R32276	SAMR_32276_Ct_hele	ABTC64312	HELEA64312	Ctenotus	helenae	inornatus−S	inornatus	SA	50 km SW Halinor Lake SA	−29.52500	130.1500	SRX2727218

Voucher or Tissue Sample	Sample ID	Tissue ID	Alternative ID	Genus	Original Taxon	Putative Unit	Putative Taxon	State or Territory	Location	Latitude	Longitude	SRA Exp. Accession
SAMA R32932	SAMR_32932_Ct_robu	ABTC14912	ROBUA14912	Ctenotus	robustus	spaldingi−S	spaldingi	SA	16 km W Penola	−37.36700	140.6500	SRX11811154
SAMA R33525	SAMR_33525_Ct_robu	ABTC03662	ROBUA03662	Ctenotus	robustus	spaldingi−S	spaldingi	NSW	Lancoona HS	−33.36700	145.8833	SRX11811155
SAMA R33560	SAMR_33560_Ct_robu	ABTC03710	ROBUA03710	Ctenotus	robustus	spaldingi−S	spaldingi	NSW	11 km W Narrabri	−30.25000	149.8500	SRX2727217
SAMA R33571	SAMAR_33571_Ct_taen	ABTC3723		Ctenotus	taeniolatus	taeniolatus	taeniolatus	NSW	21 km W Narrabrai	−30.16667	149.9333	SRX2727309
SAMA R33695	SAMR_33695_Ct_robu	ABTC03961	ROBUA03961	Ctenotus	robustus	spaldingi−S	spaldingi	NSW	Yamba tip	−29.43300	153.3667	SRX11811156
SAMA R33707	SAMR_33707_Ct_robu	ABTC04017	ROBUA04017	Ctenotus	robustus	spaldingi−S	spaldingi	NSW	25 km N Bathurst	−33.20000	149.6667	SRX2727216
SAMA R33727	SAMAR_33727_Ct_taen	ABTC4010		Ctenotus	taeniolatus	taeniolatus	taeniolatus	NSW	Denman tip	−32.38333	150.6833	SRX2727308
SAMA R33876	SAMR_33876_Ct_robu	ABTC03826	ROBUA03826	Ctenotus	robustus	spaldingi−S	spaldingi	QLD	Willowbank Caravan Pk	−27.61700	152.7833	SRX11811157
SAMA R34123	SAMR_34123_Ct_hill	ABTC11807	HILLA11807	Ctenotus	hilli	inornatus−N	inornatus	NT	Jabiru East	−12.65000	132.8833	SRX2727215
SAMA R34180	SAMAR_34180_Ct_bore	ABTC11881		Ctenotus	borealis	superciliaris−E (S)	superciliaris	NT	Tanumbirini Station	−16.45000	134.6167	SRX2727307
SAMA R34202	SAMAR_34202_Ct_spal	ABTC11919		Ctenotus	spaldingi	spaldingi−NE	spaldingi	QLD	Westmoreland Station	−17.33333	138.2500	SRX2727306
SAMA R34261	SAMAR_34261_Ct_late	ABTC11988		Ctenotus	lateralis	lateralis	lateralis	QLD	6 km E Camooweel	−19.91667	138.1667	SRX2727304
SAMA R35964	SAMR_35964_Ct_hele	ABTC00445	HELEA00445	Ctenotus	helenae	inornatus−S	inornatus	SA	Dalhousie Ruins SA	−26.51700	135.4667	SRX2727213
SAMA R36252	SAMR_36252_Ct_brac	ABTC56697	BRACA56697	Ctenotus	brachyonyx	inornatus−S	inornatus	SA	Billiat CP	−34.90000	140.4500	SRX2727210
SAMA R36313	SAMR_36313_Ct_robu	ABTC56710	ROBUA56710	Ctenotus	robustus	spaldingi−S	spaldingi	SA	Telowie Gorge	−33.01700	138.1000	SRX11811159
SAMA R36349	SAMR_36349_Ct_brac	ABTC56718		Ctenotus	brachyonyx	inornatus−S	inornatus	SA	20 km ESE Kalladeina Bore	−27.71700	139.3167	SRX2727209
SAMA R36603	SAMR_36603_Ct_robu	ABTC17100	ROBUA17100	Ctenotus	robustus	spaldingi−S	spaldingi	NSW	Esdale NW of Canberra	−35.08300	148.9167	SRX2727208
SAMA R37942	SAMR_37942_Ct_atla	ABTC57019		Ctenotus	atlas	atlas	atlas	SA	Munyaroo CP 8 km SW Moonabie HS	−33.29400	137.2083	SRX2727206
SAMA R37975	SAMR_37975_Ct_robu	ABTC57046	ROBUA57046	Ctenotus	robustus	spaldingi−S	spaldingi	SA	Burra Ck Gorge Res 17 km SE Burra	−33.83300	139.0167	SRX2727204
SAMA R38776	SAMR_38776_Ct_saxa	ABTC12005	SAXAA12005	Ctenotus	saxatilis	superciliaris−E (S)	superciliaris	NT	Phillip Creek Mission (abandoned)	−19.28300	134.2167	SRX11811160
SAMA R39480	SAMR_39480_Ct_robu	ABTC34469	ROBUA34469	Ctenotus	robustus	spaldingi−S	spaldingi	SA	20KM NE of Murray Bridge	−35.02200	139.4639	SRX11811161
SAMA R40718	SAMR_40718_Ct_robu	ABTC57450		Ctenotus	robustus	spaldingi−S	spaldingi	SA	Cooks North Middleback Rngs	−33.16700	137.1333	SRX11811162
SAMA R41330	SAMR_41330_Ct_brac	ABTC39994	BRACA39994	Ctenotus	brachyonyx	inornatus−S	inornatus	SA	Oakbank Out Stn	−33.12800	140.6056	SRX2727202
SAMA R42687	SAMAR_42687_Ct_late	ABTC8937		Ctenotus	lateralis	lateralis	lateralis	QLD	7 km NE Mt Isa Telecom Repeator Station	−20.71667	139.5500	SRX2727286
SAMA R42758	SAMR_42758_Ct_late	ABTC09000	LATEA09000	Ctenotus	lateralis	lateralis	lateralis	QLD	S Of Winton	−22.66700	142.9333	SRX2727197
SAMA R42768	SAMAR_42768_Ct_late	ABTC9010		Ctenotus	lateralis	lateralis	lateralis	QLD	Glen Kyree Station S Winton	−22.96667	142.9000	SRX2727282
SAMA R42837	SAMAR_42837_Ct_late	ABTC9082		Ctenotus	lateralis	lateralis	lateralis	QLD	43 Km N Diamantina Station	−23.51667	141.4000	SRX2727277
SAMA R42918	SAMR_42918_Ct_saxa	ABTC09164	SAXAA09164	Ctenotus	saxatilis	inornatus−S	inornatus	QLD	71 Km W of Windorah	−25.36700	141.9333	SRX2727193
SAMA R44367	SAMR_44367_Ct_hele	ABTC41506	HELEA41506	Ctenotus	helenae	inornatus−S	inornatus	SA	12.5 km ENE Mt Cooparinna	−26.34600	130.0900	SRX11811165
SAMA R44377	SAMR_44377_Ct_hele	ABTC41498	HELEA41498	Ctenotus	helenae	inornatus−S	inornatus	SA	20 km NE Mt Cooparinna	−26.29700	130.1517	SRX11811166
SAMA R45215	SAMR_45215_Ct_brac	ABTC58077	BRACA58077	Ctenotus	brachyonyx	inornatus−S	inornatus	SA	N edge Peebinga CP	−34.95800	140.8333	SRX2727184

Voucher or Tissue Sample	Sample ID	Tissue ID	Alternative ID	Genus	Original Taxon	Putative Unit	Putative Taxon	State or Territory	Location	Latitude	Longitude	SRA Exp. Accession
SAMA R45424	SAMAR_45424_Le_bipe	ABTC41733		Lerista	bipes	bipes	bipes	SA	9.4 km SE Maryinna Hill	−27.01944	131.2911	SRX11811124
SAMA R45480	SAMR_45480_Ct_saxa	ABTC41729	SAXAA41729	Ctenotus	saxatilis	inornatus−S	inornatus	SA	9.7 km S Ampeinna Hills	−27.15700	131.1311	SRX11811169
SAMA R46119	SAMR_46119_Ct_hele	ABTC41775	HELEA41775	Ctenotus	helenae	inornatus−S	inornatus	SA	27 km NE Pipalyatjara	−26.05300	129.4081	SRX11811170
SAMA R46204	SAMR_46204_Ct_robu	ABTC35548		Ctenotus	robustus	spaldingi−S	spaldingi	SA	4 km N Halifax Hill	−29.68400	135.8153	SRX11811171
SAMA R46880	SAMR_46880_Ct_saxa	ABTC36128	SAXAA36128	Ctenotus	saxatilis	inornatus−S	inornatus	SA	11.1 km ESE Mt Goodiar Witjira NP	−26.68800	135.7167	SRX11811172
SAMA R46944	SAMR_46944_Ct_leon	ABTC35989	LEONA35989	Ctenotus	leonhardii	leonhardii	leonhardii	SA	1 km WNW Top Camp Well	−26.46300	134.9458	SRX11811173
SAMA R47028	SAMR_47028_Ct_robu	ABTC36230		Ctenotus	robustus	spaldingi−S	spaldingi	SA	35 km ENE Kingston SE	−36.73800	140.2397	SRX2727175
SAMA R48684	SAMR_48684_Ct_robu	ABTC58575	ROBUA58575	Ctenotus	robustus	spaldingi−S	spaldingi	SA	Moonaree Stn	−31.70500	135.5256	SRX11811174
SAMA R48710	SAMR_48710_Ct_hele	ABTC41974	HELEA41974	Ctenotus	helenae	inornatus−S	inornatus	SA	4 km W Mt Lindsay SA	−27.02900	129.8392	SRX11811176
SAMA R48962	SAMR_48962_Ct_robu	ABTC37169	ROBUA37169	Ctenotus	robustus	spaldingi−S	spaldingi	SA	Andamooka Stn	−30.74200	137.3136	SRX11811177
SAMA R49609	SAMR_49609_Ct_robu	ABTC37540		Ctenotus	robustus	spaldingi−S	spaldingi	SA	7 km NE Hatherleigh SA	−37.43800	140.3136	SRX11811179
SAMA R50208	SAMR_50208_Ct_saxa	ABTC42206	SAXAA42206	Ctenotus	saxatilis	inornatus−S	inornatus	SA	11.2 km SW Sentinel Hill	−26.14100	132.3592	SRX2727163
SAMA R50209	SAMR_50209_Ct_saxa	ABTC42208	SAXAA42208	Ctenotus	saxatilis	inornatus−S	inornatus	SA	11.2 km SW Sentinel Hill	−26.14100	132.3592	SRX2727162
SAMA R50372	SAMR_50372_Ct_robu	ABTC38130	ROBUA38130	Ctenotus	robustus	spaldingi−S	spaldingi	SA	1 km SE Mt Aroona	−30.58400	138.3689	SRX11811180
SAMA R51093	SAMAR_51093_Ct_spal	ABTC13415		Ctenotus	spaldingi	spaldingi−NE	spaldingi	NT	2 km S Elliott	−17.56667	133.5333	SRX2727246
SAMA R52303	SAMR_52303_Ct_robu	ABTC39414	ROBUA39414	Ctenotus	robustus	spaldingi−S	spaldingi	SA	1.5 km S Camel Yard Spring	−30.66000	139.0911	SRX11811181
SAMA R52956	SAMR_52956_Ct_robu	ABTC74190	ROBUA74190	Ctenotus	robustus	spaldingi−S	spaldingi	SA	Arkaroola	−30.11900	139.4483	SRX2727157
SAMA R53975	SAMR_53975_Ct_saxa	ABTC72677		Ctenotus	saxatilis	superciliaris−E (S)	superciliaris	NT	Barrow Creek channel just N town	−21.50000	133.9000	SRX2727155
SAMA R54008	SAMR_54008_Ct_saxa	ABTC72702	SAXAA72702	Ctenotus	saxatilis	inornatus−S	inornatus	NT	2 km N Barrow Creek	−21.50600	133.8939	SRX2727154
SAMA R54340	SAMR_54340_Ct_late	ABTC72759	LATEA72759	Ctenotus	lateralis	lateralis	lateralis	QLD	East Leichardt Dam	−20.77500	139.7856	SRX2727149
SAMA R54433	SAMAR_54433_Ct_late	ABTC72838		Ctenotus	lateralis	lateralis	lateralis	QLD	21 km S Burke & Wills RH	−19.39472	140.2367	SRX2727241
SAMA R54434	SAMR_54434_Ct_late	ABTC72839	LATEA72839	Ctenotus	lateralis	lateralis	lateralis	QLD	21 km S Burke & Wills RH	−19.39500	140.2367	SRX2727147
SAMA R54463	SAMAR_54463_Ct_late	ABTC72875		Ctenotus	lateralis	lateralis	lateralis	QLD	Burke & Wills RH Dump	−19.22639	140.3481	SRX2727240
SAMA R54485	SAMAR_54485_Ct_spal	ABTC72903		Ctenotus	spaldingi	spaldingi−NE	spaldingi	QLD	22 km S Torrens Creek	−20.98583	145.0317	SRX2727239
SAMA R55259	SAMAR_55259_Ct_late	ABTC82415		Ctenotus	lateralis	lateralis	lateralis	QLD	Phosphate Hill Bloodwood site	−21.91306	140.0217	SRX2727238
SAMA R55675	SAMAR_55675_Ct_spal	ABTC76994		Ctenotus	spaldingi	spaldingi−NE	spaldingi	QLD	40 km S Torrens Creek on Torrens Creek−Aramac Road	−21.09167	145.0044	SRX2727236
SAMA R55725	SAMR_55725_Ct_spal	ABTC77041	SPALA77041	Ctenotus	spaldingi	spaldingi−NE	spaldingi	QLD	33 km NNE Hughenden on Kennedy Developmental Rd	−20.62400	144.3997	SRX2727138
SAMA R55731	SAMAR_55731_Ct_robu	ABTC77047		Ctenotus	robustus	spaldingi−S	spaldingi	QLD	6.5 km E Julia Creek on Flinders Highway	−20.65528	141.7975	SRX2727233
SAMA R55746	SAMR_55746_Ct_robu	ABTC77063	ROBUA77063	Ctenotus	robustus	spaldingi−S	spaldingi	QLD	37 km SSE Julia Ck	−20.97500	141.8917	SRX2727136
SAMA R55799	SAMAR_55799_Ct_late	ABTC77111		Ctenotus	lateralis	eutaenius	cf. eutaenius	QLD	35 km E Mt Surprise on Gulf Developmental Road	−18.12833	144.6328	SRX2727232
SAMA R55874	SAMR_55874_Ct_euta	ABTC77199	EUTAA77199	Ctenotus	eutaenius	eutaenius	cf. eutaenius	QLD	Charters Towers	−20.08900	146.2525	SRX2727135

Voucher or Tissue Sample	Sample ID	Tissue ID	Alternative ID	Genus	Original Taxon	Putative Unit	Putative Taxon	State or Territory	Location	Latitude	Longitude	SRA Exp. Accession
SAMA R57397	SAMR_57397_Ct_atla	ABTC95560	ATLAA95560	Ctenotus	atlas	atlas	atlas	SA	9.5 km SSW Murninnie	−33.39389	137.3428	SRX2727130
SAMA R65339	SAMR_65339_Ct_late	ABTC113697		Ctenotus	lateralis	lateralis	lateralis	QLD	23.5 km NE Alderley Homestead	−22.38500	139.8564	SRX2727119
SAMA R65407	SAMR_65407_Ct_late	ABTC113882		Ctenotus	lateralis	lateralis	lateralis	QLD	17.1 km W Noonbah Homestead	−24.10167	143.0175	SRX2727113
SAMA R65416	SAMR_65416_Ct_late	ABTC113822		Ctenotus	lateralis	lateralis	lateralis	QLD	3.7 km SSE Noonbah Homestead	−24.13611	143.2017	SRX2727112
UMMZ 242606	UMMZ_242606_Ct_aust	UMFS20669		Ctenotus	australis	australis	australis	WA	Tamala Station	−26.65780	113.6476	SRX2727110
UMMZ 242607	UMMZ_242607_Ct_aust	UMFS20670		Ctenotus	australis	australis	australis	WA	Tamala Station	−26.65758	113.6470	SRX2727109
UMMZ 242614	UMMZ_242614_Ct_hele	UMFS20505		Ctenotus	helenae	inornatus−S	inornatus	WA	Dampier Downs Station	−18.11428	123.5507	SRX2727105
UMMZ 242616	UMMZ_242616_Ct_hele	UMFS20510		Ctenotus	helenae	inornatus−S	inornatus	WA	Dampier Downs Station	−18.11428	123.5507	SRX2727104
UMMZ 242623	UMMZ_242623_Ct_hele	UMFS20603		Ctenotus	helenae	inornatus−S	inornatus	WA	Stretch Lagoon	−19.67899	127.5867	SRX2727103
UMMZ 242624	UMMZ_242624_Ct_inor	UMFS20506		Ctenotus	inornatus	superciliaris−W	superciliaris	WA	Dampier Downs Station	−18.09191	123.5694	SRX2727102
UMMZ 242625	UMMZ_242625_Ct_inor	UMFS20516		Ctenotus	inornatus	superciliaris−W	superciliaris	WA	Dampier Downs Station	−18.11428	123.5507	SRX2727101
UMMZ 242629	UMMZ_242629_Ct_inor	UMFS20597		Ctenotus	inornatus	superciliaris−W	superciliaris	WA	Carranya Station	−19.24250	127.7828	SRX2727100
UMMZ 242630	UMMZ_242630_Ct_inor	UMFS20599		Ctenotus	inornatus	superciliaris−W	superciliaris	WA	Carranya Station	−19.24257	127.7806	SRX2727099
UMMZ 242633	UMMZ_242633_Ct_pant	UMFS20512		Ctenotus	pantherinus	pantherinus	pantherinus	WA	Dampier Downs Station	−18.11428	123.5507	SRX2727098
UMMZ 242639	UMMZ_242639_Ct_pant	UMFS20600		Ctenotus	pantherinus	pantherinus	pantherinus	WA	Carranya Station	−19.23930	127.8264	SRX2727095
UMMZ 244288	UMMZ_244288_ct_inor	UMFS21396		Ctenotus	fallens	inornatus−S	inornatus	WA	Peron Peninsula Site PP1	−26.05799	113.6156	NA
WAM R084577	WAMR_084577_Ct_hele	WAMR084577	HELEW084577	Ctenotus	helenae	inornatus−S	inornatus	WA	North Lake Throssell	−27.25000	124.4167	SRX2727083
WAM R094929	WAMR_094929_Ct_hele	WAMR094929	HELEW094929	Ctenotus	helenae	inornatus−S	inornatus	WA	Thompson Hills	−21.33300	124.7500	SRX11811187
WAM R102423	WAMR_102423_Ct_saxa	WAMR102423	SAXAW102423	Ctenotus	saxatilis	inornatus−S	inornatus	WA	Barlee Range Nature Reserve	−23.10200	116.0078	SRX11811188
WAM R102671	WAMR_102671_Ct_hele	WAMR102671	HELEW102671	Ctenotus	helenae	inornatus−S	inornatus	WA	Little Sandy Desert	−24.05400	120.4067	SRX2727074
WAM R102771	WAMR_102771_Ct_hele	WAMR102771	HELE102771	Ctenotus	helenae	inornatus−S	inornatus	WA	Little Sandy Desert	−24.53200	120.2911	SRX11811191
WAM R108698	WAMR_108698_Ct_saxa	WAMR108698	SAXAW108698	Ctenotus	saxatilis	superciliaris−W	superciliaris	WA	Cheerabun HS	−18.18300	125.1167	SRX11811085
WAM R108742	WAMR_108742_Ct_hele	WAMR108742	HELEW108742	Ctenotus	helenae	inornatus−S	inornatus	WA	Banana Springs	−18.90000	128.8000	SRX2727071
WAM R108745	WAMR_108745_Ct_saxa	WAMR108745	SAXAW108745	Ctenotus	saxatilis	superciliaris−W	superciliaris	WA	Mabel Downs Station	−17.28300	128.1833	SRX11811087
WAM R108766	WAMR_108766_Ct_saxa	WAMR108766	SAXAW108766	Ctenotus	saxatilis	superciliaris−W	superciliaris	WA	Supplejack Bore	−18.91700	125.2667	SRX2727070
WAM R108816	WAMR_108816_Ct_saxa	WAMR108816	SAXAW108816	Ctenotus	saxatilis	superciliaris−W	superciliaris	WA	Cherralta Homestead	−21.03300	116.8167	SRX11811066
WAM R108911	WAMR_108911_Ct_hele	WAMR108911	HELEW108911	Ctenotus	helenae	inornatus−S	inornatus	WA	Telfer	−21.88300	122.3667	SRX2727069
WAM R111809	WAMR_111809_Le_bipe	WAMR111809		Lerista	bipes	bipes	bipes	WA	Bdrs01	−23.39200	120.5220	SRX11811067
WAM R115114	WAMR_115114_Ct_aust	WAMR115114	AUSTW115114	Ctenotus	australis	australis	australis	WA	Ken Hearst Park	−32.08300	115.8833	SRX2727059
WAM R115119	WAMR_115119_Ct_aust	WAMR115119	AUSTW115119	Ctenotus	australis	australis	australis	WA	Bold Park	−31.93300	115.7703	SRX2727058
WAM R117679	WAMR_117679_Ct_saxa	WAMR117679	SAXAW117679	Ctenotus	saxatilis	superciliaris−W	superciliaris	WA	Varanus Island	−20.56700	115.5667	SRX2727055

Voucher or Tissue Sample	Sample ID	Tissue ID	Alternative ID	Genus	Original Taxon	Putative Unit	Putative Taxon	State or Territory	Location	Latitude	Longitude	SRA Exp. Accession
WAM R126010	WAMR_126010_Ct_rima	WAMR126010	RIMAW126010	Ctenotus	rimacola	rimacola	rimacola	NA	Mt Septimus	−15.5870	128.9961	SRX2727048
WAM R126015	WAMR_126015_Ct_rima	WAMR126015	RIMAW126015	Ctenotus	rimacola	rimacola	rimacola	WA	Septimus Mount	−15.5870	128.9961	SRX2727047
WAM R126017	WAMR_126017_Ct_inor	WAMR126017	INORW126017	Ctenotus	inornatus	inornatus−N	inornatus	WA	Kimberley Research Station	−15.6580	128.6864	SRX11811069
WAM R129923	WAMR_129923_Ct_hele	WAMR129923	HELEW129923	Ctenotus	helenae	inornatus−S	inornatus	WA	West Angelas	−23.2500	118.6667	SRX11811073
WAM R131009	WAMR_131009_Ct_hele	WAMR131009	HELEW131009	Ctenotus	helenae	inornatus−S	inornatus	WA	Millstream−Chichester NP	−21.1900	117.1722	SRX11811074
WAM R131018	WAMR_131018_Ct_fall	WAMR131018	FALLW131018	Ctenotus	fallens	inornatus−S	inornatus	WA	Hamelin Homestead	−26.5670	114.2333	SRX11811075
WAM R131371	WAMR_131371_Ct_hele	WAMR131371	HELEW131371	Ctenotus	helenae	inornatus−S	inornatus	WA	Nanutarra Roadhouse	−22.8330	115.0333	SRX11811077
WAM R132522	WAMR_132522_Ct_saxa	WAMR132522	SAXAW132522	Ctenotus	saxatilis	superciliaris−W	superciliaris	WA	Burrup Peninsula	−20.6160	116.7850	SRX11811079
WAM R132682	WAMR_132682_Ct_saxa	WAMR132682	SAXAW132682	Ctenotus	saxatilis	superciliaris−W	superciliaris	WA	Shay Gap	−20.6160	120.2761	SRX11811080
WAM R132686	WAMR_132686_Ct_saxa	WAMR132686		Ctenotus	saxatilis	superciliaris−W	superciliaris	WA	Shay Gap	−20.5790	120.3186	SRX11811081
WAM R132723	WAMR_132723_Ct_saxa	WAMR132723	SAXAW132723	Ctenotus	saxatilis	superciliaris−W	superciliaris	WA	Goldsworthy	−20.3180	119.4233	SRX11811082
WAM R135152	WAMR_135152_Le_ips	WAMR135152		Lerista	ips	ips	ips	WA	Near Telfer Dome	−22.1833	122.2667	SRX11811083
WAM R135396	WAMR_135396_Ct_hele	WAMR135396	HELEW135396	Ctenotus	helenae	inornatus−S	inornatus	WA	Mt Brockman Station	−22.4200	117.4300	SRX11811089
WAM R135692	WAMR_135692_Ct_saxa	WAMR135692	SAXAW135692	Ctenotus	saxatilis	superciliaris−W	superciliaris	WA	Broome	−17.9830	122.3333	SRX2727029
WAM R137950	WAMR_137950_Ct_robu	WAMR137950	ROBUW137950	Ctenotus	robustus	robustus−NW	robustus	WA	Kununurra	−15.5890	128.9833	SRX11811090
WAM R139005	WAMR_139005_Ct_saxa	WAMR139005		Ctenotus	saxatilis	superciliaris−W	superciliaris	WA	Mandora	−19.7460	121.4575	SRX11811092
WAM R139296	WAMR_139296_Ct_hele	WAMR139296	HELEW139296	Ctenotus	helenae	inornatus−S	inornatus	WA	Meentheena	−21.2450	120.3222	SRX2727593
WAM R139414	WAMR_139414_Ct_nigr	WAMR139414	NIGRW139414	Ctenotus	nigrilineatus	nigrilineatus	nigrilineatus	WA	Meentheena	−21.2870	120.4594	SRX2727590
WAM R139415	WAMR_139415_Ct_nigr	WAMR139415	NIGRW139415	Ctenotus	nigrilineatus	nigrilineatus	nigrilineatus	WA	Meentheena	−21.2900	120.4664	SRX2727589
WAM R139523	WAMR_139523_Ct_hele	WAMR139523	HELEW139523	Ctenotus	helenae	inornatus−S	inornatus	WA	Giralia	−22.8260	114.4447	SRX2727587
WAM R140720	WAMR_140720_Ct_hele	WAMR140720		Ctenotus	helenae	inornatus−S	inornatus	WA	Hope Downs	−22.8210	119.3258	SRX11811094
WAM R141131	WAMR_141131_Ct_hele	WAMR141131	HELEW141131	Ctenotus	helenae	inornatus−S	inornatus	WA	Leinster Downs Station	−27.9650	120.3892	SRX2727582
WAM R141301	WAMR_141301_Ct_hele	WAMR141301	HELEW141301	Ctenotus	helenae	inornatus−S	inornatus	WA	Cape Preston	−21.0660	116.1492	SRX2727579
WAM R141379	WAMR_141379_Ct_robu	WAMR141379	ROBUW141379	Ctenotus	robustus	robustus−NW	robustus	WA	Cape Preston	−21.1020	116.1317	SRX2727578
WAM R145567	WAMR_145567_Ct_hele	WAMR145567		Ctenotus	helenae	inornatus−S	inornatus	WA	Port Hedland	−20.6100	118.6100	SRX11811096
WAM R145686	WAMR_145686_Ct_robu	WAMR145686	ROBUW145686	Ctenotus	robustus	robustus−NW	robustus	WA	Abydos	−22.1010	118.9914	SRX2727572
WAM R145698	WAMR_145698_Ct_hele	WAMR145698		Ctenotus	helenae	inornatus−S	inornatus	WA	Weeli Wolli Creek	−22.9580	119.1789	SRX11811097
WAM R145926	WAMR_145926_Ct_hele	WAMR145926	HELEW145926	Ctenotus	helenae	inornatus−S	inornatus	WA	Cundeelee	−30.7230	123.4239	SRX2727571
WAM R146012	WAMR_146012_Ct_robu	WAMR146012	ROBUW146012	Ctenotus	robustus	robustus−NW	robustus	WA	Kimbolton	−16.7430	124.0950	SRX2727570
WAM R146913	WAMR_146913_Ct_seve	WAMR146913	SEVEW146913	Ctenotus	severus	inornatus−S	inornatus	WA	Mount Gibson	−29.5850	117.2703	SRX2727567
WAM R152991	WAMR_152991_Ct_seve	WAMR152991	SEVEW152991	Ctenotus	severus	inornatus−S	inornatus	WA	Walga Rock	−27.3990	117.4708	SRX2727561

Voucher or Tissue Sample	Sample ID	Tissue ID	Alternative ID	Genus	Original Taxon	Putative Unit	Putative Taxon	State or Territory	Location	Latitude	Longitude	SRA Exp. Accession
WAM R153812	WAMR_153812_Ct_saxa	WAMR153812	SAXAW153812	Ctenotus	saxatilis	superciliaris−W	superciliaris	WA	Yardie Homestead Caravan	−21.89400	114.0094	SRX11811098
WAM R154016	WAMR_154016_Ct_fall	WAMR154016	FALLW154016	Ctenotus	fallens	inornatus−S	inornatus	WA	Muchea	−31.64200	115.9175	SRX2727559
WAM R156159	WAMR_156159_Ct_seve	WAMR156159	SEVEW156159	Ctenotus	severus	inornatus−S	inornatus	WA	Waldburg Homestead	−24.75000	117.3667	SRX2727557
WAM R157646	WAMR_157646_Ct_hele	WAMR157646	HELEW157646	Ctenotus	helenae	inornatus−S	inornatus	WA	Newman	−23.31200	119.7956	SRX2727549
WAM R157958	WAMR_157958_Le_ips	WAMR157958		Lerista	ips	ips	ips	WA	Lake Disappointment	−23.29630	122.6769	SRX11811099
WAM R158204	WAMR_158204_Ct_saxa	WAMR158204	SAXAW158204	Ctenotus	saxatilis	superciliaris−W	superciliaris	WA	Roy Hill	−22.40300	119.8611	SRX2727547
WAM R158376	WAMR_158376_Ct_saxa	WAMR158376	SAXAW158376	Ctenotus	saxatilis	superciliaris−W	superciliaris	WA	Giralia	−22.64400	114.4150	SRX2727545
WAM R166390	WAMR_166390_Ct_hele	ABTC91786	HELEA91786	Ctenotus	helenae	inornatus−S	inornatus	WA	Morgan Range	−25.93860	128.3897	SRX11811100
WAM R166391	WAMR_166391_Ct_hele	ABTC91631	HELEA91631	Ctenotus	helenae	inornatus−S	inornatus	WA	0.5 km E Pungkulpirri Waterhole Walter James Ranges	−24.65420	128.7553	SRX2727533
WAM R166392	WAMR_166392_Ct_hele	ABTC91638	HELEA91638	Ctenotus	helenae	inornatus−S	inornatus	WA	Kutjuntari Rockhole	−24.89140	128.7692	SRX2727532
WAM R174671	NA_CCM1389_Ct_robu			Ctenotus	robustus	robustus−NW	robustus	WA	Potts Black soil	−16.46680	125.3723	SRX2727399
WAM R174672	NA_CCM1580_Ct_inor			Ctenotus	inornatus	inornatus−N	inornatus	WA	Police valley camp	−16.83117	126.2281	SRX2727386
WAM R174673	NA_CCM1061_Ct_sp			Ctenotus	sp	mastigura	cf. mastigura	WA	Gibb River Camp	−16.09690	126.5112	SRX2727403
WAM R174674	NA_CCM0980_Ct_mast			Ctenotus	mastigura	mastigura	cf. mastigura	WA	Baitbox Hill Theda site 3 10 km N of HS	−14.74225	126.4669	SRX2727406
WAM R174675	NA_CCM0753_Ct_robu			Ctenotus	robustus	mastigura	cf. mastigura	WA	Old Mitchell Road	−15.13930	126.1571	SRX2727417
WAM R174676	NA_CCM1206_Ct_robu			Ctenotus	robustus	mastigura	cf. mastigura	WA	Mitchell Plateau Surveyors Pool	−14.67100	125.7330	SRX2727402
WAM R174677	NA_CCM1249_Ct_inor			Ctenotus	inornatus	inornatus−N	inornatus	WA	Silent Grove ranger station	−17.06662	125.2501	SRX2727401
WAM R174680	NA_CCM1445_Ct_inor			Ctenotus	inornatus	inornatus−N	inornatus	WA	Chamberlain valley riparian	−17.29230	127.1720	SRX2727391
WAM R174681	NA_CCM1529_Ct_inor			Ctenotus	inornatus	mastigura	cf. mastigura	WA	Hahn River rocks	−16.81627	126.0768	SRX2727389
WAM R174683	NA_CCM1388_Ct_robu			Ctenotus	robustus	robustus−NW	robustus	WA	Potts riparian	−16.49270	125.3447	SRX2727400
WAM R174685	NA_CCM1579_Ct_inor			Ctenotus	inornatus	mastigura	cf. mastigura	WA	Barnett River Gorge	−16.53559	126.1286	SRX2727387
WAM R174687	NA_CCM1390_Ct_inor			Ctenotus	inornatus	mastigura	cf. mastigura	WA	Grevillea gorge	−16.49954	125.3364	SRX2727398
WAM R174689	NA_CCM1530_Ct_robu			Ctenotus	robustus	inornatus−N	inornatus	WA	Police Valley site	−16.82743	126.2164	SRX2727388
WAM R174690	NA_CCM1444_Ct_inor			Ctenotus	inornatus	inornatus−N	inornatus	WA	Chamberlain valley woodland	−17.29320	127.2175	SRX2727392

Table S2. Mitochondrial (cytb) sample information.

Voucher or Tissue Sample	Sample ID	Tissue ID	Alternative ID	Original Taxon	OTU	Putative Taxon	State or Territory	Location	Latitude	Longitude	GenBank Accession
ABTC03841	NA_ABTC03841_Ct_robu	ABTC03841	ROBUA03841	robustus	spaldingi−S	spaldingi	QLD	14 km W Cooyar	−27.0000	151.8300	KJ505611
ABTC12568	NA_ABTC12568_Ct_hele	ABTC12568	HELEA12568	helenae	inornatus−S	inornatus	NT	Kings Creek Stn	−24.4330	131.8167	KJ505394
ABTC21767	NA_ABTC21767_Ct_inor	ABTC21767	INORA21767	inornatus	inornatus−N	inornatus	NT	Cape Arnhem	−12.3300	136.9500	KJ505485
ABTC23980	NA_ABTC23980_Ct_hele	ABTC23980	HELEA23980	helenae	inornatus−S	inornatus	NT	Uluru NP	NA	NA	KJ505395
ABTC23990	NA_ABTC23990_Ct_hele	ABTC23990	HELEA23990	helenae	inornatus−S	inornatus	NT	Uluru NP	NA	NA	KJ505396
ABTC24077	NA_ABTC24077_Ct_hele	ABTC24077	HELEA24077	helenae	inornatus−S	inornatus	NT	MacDonnell Ranges	−23.8000	132.5000	KJ505397
ABTC24098	NA_ABTC24098_Ct_hele	ABTC24098	HELEA24098	helenae	inornatus−S	inornatus	NT	MacDonnell Ranges	NA	NA	KJ505398
ABTC31797	NA_ABTC31797_Ct_saxa	ABTC31797	SAXAA31797	saxatilis	inornatus−S	inornatus	NT	Log Cabin Dam MacDonnell Ranges	−23.8000	133.2000	KJ505693
ABTC31798	NA_ABTC31798_Ct_saxa	ABTC31798	SAXAA31798	saxatilis	inornatus−S	inornatus	NT	Log Cabin Dam MacDonnell Ranges	−23.8000	133.2000	KJ505694
ABTC60756	NA_ABTC60756_Ct_hele	ABTC60756	HELEA60756	helenae	inornatus−S	inornatus	NT	30 km SW Sangster's Bore	−20.8300	130.4200	KJ505405
ABTC60781	NA_ABTC60781_Ct_hele	ABTC60781	HELEA60781	helenae	inornatus−S	inornatus	NT	30 km SW Sangster's Bore	−20.8300	130.4200	KJ505406
AMR102758	AMR_102758_Ct_robu	ABTC11084	ROBUA11084	robustus	spaldingi−S	spaldingi	ACT	Brindabella Range	−35.4000	148.8000	KJ505615
AMSR111493	AMSR_111493_Ct_spal	ABTC11090		spaldingi	spaldingi−CY	spaldingi	QLD	Watson River	−13.3640	141.7300	OQ091785
AMSR111494	AMSR_111494_Ct_spal	ABTC11091		spaldingi	spaldingi−CY	spaldingi	QLD	False Pera Head	−13.0830	141.6160	OQ091786
ANWCR05240	ANWC_R05240_Ct_spal	ABTC106062	ROBUA106062	spaldingi	spaldingi−CY	spaldingi	QLD	Eastern Mcilwraith Range Lowlands	−13.7806	143.5028	KJ506051
ANWCR05241	ANWC_R05241_Ct_spal	ABTC106063		spaldingi	spaldingi−CY	spaldingi	QLD	Eastern Mcilwraith Range Lowlands	−13.7806	143.5028	KJ506003
ANWCR05242	ANWC_R05242_Ct_spal	ABTC106064		spaldingi	spaldingi−CY	spaldingi	QLD	Eastern Mcilwraith Range Lowlands	−13.7806	143.5028	KJ506053
ANWCR05268	ANWC_R05268_Ct_spal	ABTC106065	ROBUA106065	spaldingi	spaldingi−CY	spaldingi	QLD	Eastern Mcilwraith Range Lowlands	−13.8861	143.4806	KJ506004
ANWCR05369	ANWC_R05369_Ct_robu	ABTC106058	ROBUA106058	robustus	spaldingi−NE	spaldingi	QLD	Shoalwater Bay Army Training Reserve N Rockhampton	−22.2606	150.5211	KJ506050
ANWCR05400	ANWC_R05400_Ct_robu	ABTC106059	ROBUA106059	robustus	spaldingi−NE	spaldingi	QLD	Shoalwater Bay Army Training Reserve N Rockhampton	−22.7097	150.6292	KJ505999
ANWCR05425	ANWC_R05425_Ct_robu	ABTC106060	ROBUA106060	robustus	spaldingi−NE	spaldingi	QLD	Shoalwater Bay Army Training Reserve N Rockhampton	−22.7083	150.2750	KJ506000
ANWCR05463	ANWC_R05463_Ct_robu	ABTC106061	ROBUA106061	robustus	spaldingi−NE	spaldingi	QLD	Shoalwater Bay Army Training Reserve N Rockhampton	−22.4361	150.3333	KJ506001
ANWCR06669	ANWC_R06669_Ct_robu	ABTC106057	ROBUA106057	robustus	spaldingi−S	spaldingi	NSW	Kilnyana 19.5 km SE Berrigan Savernake Area	−35.7864	146.9597	KJ506049
ANWCR06670	ANWC_R06670_Ct_robu	ABTC106055	ROBUA106055	robustus	spaldingi−S	spaldingi	NSW	Arbourfollie 3 km SE Berrigan Savernake Area	−35.6819	146.8256	KJ505998
ANWCR06679	ANWC_R06679_Ct_robu	ABTC106056		robustus	spaldingi−S	spaldingi	NSW	Brynton 15 km SSE Berrigan Savernake Area	−35.7897	145.8542	KJ506048
CCM0044	NA_CCM0044_Ct_euta			eutaenius	spaldingi−S	spaldingi	QLD	The Vines Magnetic Island	−19.1326	146.8690	OQ091801
CCM0090	NA_CCM0090_Ct_late			lateralis	eutaenius	cf. eutaenius	QLD	Cobb Gorge Cobb12	−18.8212	143.4066	OQ091802
CCM0535	NA_CCM0535_Ct_inor			inornatus	superciliaris−W	superciliaris	NT	Vic River Region Escarpment Walk	−15.6109	131.1160	OQ091803
CCM0753	NA_CCM0753_Ct_robu			robustus	mastigura	cf. mastigura	WA	Old Mitchell Road	−15.1393	126.1571	OQ091804
CCM0823	NA_CCM0823_Ct_robu			robustus	mastigura	cf. mastigura	WA	Carson escarpment TD1	−15.3383	126.5917	OQ091805

Voucher or Tissue Sample	Sample ID	Tissue ID	Alternative ID	Original Taxon	OTU	Putative Taxon	State or Territory	Location	Latitude	Longitude	GenBank Accession
CCM0830	NA_CCM0830_Ct_inor			inornatus	mastigura	cf. mastigura	WA	Carson escarpment near camp	−15.3394	126.5893	OQ091806
CCM0847	NA_CCM0847_Ct_robu			robustus	mastigura	cf. mastigura	WA	Old Mitchell Q5	−15.1328	126.1478	OQ091807
CCM0848	NA_CCM0848_Ct_inor			inornatus	inornatus−N	inornatus	WA	Spring Creek SS2	−15.2002	125.9067	OQ091808
CCM0850	NA_CCM0850_Ct_inor			inornatus	mastigura	cf. mastigura	WA	Spring Creek SS1	−15.1989	125.9035	OQ091809
CCM0946	NA_CCM0946_Ct_inor			inornatus	mastigura	cf. mastigura	WA	King Ed Riv Theda campsite 30 km N of Homestead	−14.5190	126.4581	OQ091810
CCM0947	NA_CCM0947_Ct_inor			inornatus	mastigura	cf. mastigura	WA	Baitbox Hill Theda site 7 10 km N of HS	−14.7312	126.4600	OQ091811
CCM0956	NA_CCM0956_Ct_robu			robustus	robustus−NW	robustus	WA	Baitbox Hill Theda site 2 10 km N of HS	−14.7519	126.4772	OQ091812
CCM0980	NA_CCM0980_Ct_mast			mastigura	mastigura	cf. mastigura	WA	Baitbox Hill Theda site 3 10 km N of HS	−14.7423	126.4669	OQ091813
CCM1206	NA_CCM1206_Ct_robu			robustus	mastigura	cf. mastigura	WA	Mitchell Plateau Surveyors Pool	−14.6710	125.7330	OQ091814
CCM1249	NA_CCM1249_Ct_inor			inornatus	inornatus−N	inornatus	WA	Silent Grove ranger station	−17.0666	125.2501	OQ091815
CCM1388	NA_CCM1388_Ct_robu			robustus	robustus−NW	robustus	WA	Potts riparian	−16.4927	125.3447	OQ091816
CCM1389	NA_CCM1389_Ct_robu			robustus	robustus−NW	robustus	WA	Potts Black soil	−16.4668	125.3723	OQ091817
CCM1390	NA_CCM1390_Ct_inor			inornatus	mastigura	cf. mastigura	WA	Grevillea gorge	−16.4995	125.3364	OQ091818
CCM1409	NA_CCM1409_Ct_robu			robustus	robustus−NW	robustus	WA	Potts Black soil	−16.4668	125.3723	OQ091819
CCM1412	NA_CCM1412_Ct_robu			robustus	robustus−NW	robustus	WA	Potts riparian	−16.4940	125.3417	OQ091820
CCM1415	NA_CCM1415_Ct_robu			robustus	robustus−NW	robustus	WA	Potts riparian campsite	−16.4894	125.3518	OQ091821
CCM1444	NA_CCM1444_Ct_inor			inornatus	inornatus−N	inornatus	WA	Chamberlain valley woodland	−17.2932	127.2175	OQ091822
CCM1445	NA_CCM1445_Ct_inor			inornatus	inornatus−N	inornatus	WA	Chamberlain valley riparian	−17.2923	127.1720	OQ091823
CCM1529	NA_CCM1529_Ct_inor			inornatus	mastigura	cf. mastigura	WA	Hahn River rocks	−16.8163	126.0768	OQ091824
CCM1530	NA_CCM1530_Ct_robu			robustus	inornatus−N	inornatus	WA	Police Valley site	−16.8274	126.2164	OQ091825
CCM1579	NA_CCM1579_Ct_inor			inornatus	mastigura	cf. mastigura	WA	Barnett River Gorge	−16.5356	126.1286	OQ091826
CCM1580	NA_CCM1580_Ct_inor			inornatus	inornatus−N	inornatus	WA	Police valley camp	−16.8312	126.2281	OQ091827
CCM1710	NA_CCM1710_Ct_inor			inornatus	inornatus−N	inornatus	NT	Willeroo station tip	−15.2961	131.5851	OQ091828
CCM1988	NA_CCM1988_Ct_inor	CCM1988		inornatus	superciliaris−E (N)	superciliaris	NT	Kakadu fire plot 64B	−13.1793	132.9871	OQ091829
CCM1990	NA_CCM1990_Ct_inor	CCM1990		inornatus	superciliaris−E (N)	superciliaris	NT	Kakadu fire plot 64B	−13.1793	132.9871	OQ091830
CCM1991	NA_CCM1991_Ct_vert	CCM1991		vertebralis	superciliaris−E (N)	superciliaris	NT	Kakadu fire plot 64	−13.1784	132.9882	OQ091831
CCM1993	NA_CCM1993_Ct_inor	CCM1993		inornatus	superciliaris−E (N)	superciliaris	NT	Kakadu fire plot 64B	−13.1793	132.9871	OQ091832
CCM1994	NA_CCM1994_Ct_inor	CCM1994		inornatus	superciliaris−E (N)	superciliaris	NT	Kakadu fire plot 64	−13.1784	132.9882	OQ091833
CCM1995	NA_CCM1995_Ct_inor	CCM1995		inornatus	superciliaris−E (N)	superciliaris	NT	Kakadu fire plot 64	−13.1784	132.9882	OQ091834
CCM2023	NA_CCM2023_Ct_asti	CCM2023		astictus	superciliaris−E (S)	superciliaris	NT	Hartz Range camp Roper Hwy	−14.7112	134.2886	OQ091835

Voucher or Tissue Sample	Sample ID	Tissue ID	Alternative ID	Original Taxon	OTU	Putative Taxon	State or Territory	Location	Latitude	Longitude	GenBank Accession
CCM2169	NA_CCM2169_Ct_supe	CCM2169		superciliaris	superciliaris−E (S)	superciliaris	NT	Hartz Range camp Roper Hwy	−14.7112	134.2886	OQ091836
CCM2175	NA_CCM2175_Ct_supe	CCM2175		superciliaris	superciliaris−E (S)	superciliaris	NT	Hartz Range camp Roper Hwy	−14.7112	134.2886	OQ091837
CCM2196	NA_CCM2196_Ct_asti	CCM2196		astictus	superciliaris−E (S)	superciliaris	NT	Tomato Island tip	−14.7567	134.6849	OQ091838
CCM2197	NA_CCM2197_Ct_asti	CCM2197		astictus	superciliaris−E (S)	superciliaris	NT	Tomato Island tip	−14.7567	134.6849	OQ091839
CCM2292	NA_CCM2292_Ct_inor	CCM2292		inornatus	superciliaris−E (N)	superciliaris	NT	Kakadu fire plot 27	−13.9428	132.9029	OQ091840
CCM2316	NA_CCM2316_Ct_vert	CCM2316		vertebralis	superciliaris−E (N)	superciliaris	NT	Kakadu fire plot 65B	−13.3525	132.9854	OQ091841
CCM2317	NA_CCM2317_Ct_vert	CCM2317		vertebralis	superciliaris−E (N)	superciliaris	NT	Kakadu fire plot 65	−13.3503	132.9849	OQ091842
CCM2320	NA_CCM2320_Ct_vert	CCM2320		vertebralis	superciliaris−E (N)	superciliaris	NT	Kakadu fire plot 65	−13.3503	132.9849	OQ091843
CCM2331	NA_CCM2331_Ct_inor	CCM2331		inornatus	superciliaris−E (N)	superciliaris	NT	Kakadu fire plot 29	−13.2817	132.8453	OQ091844
CCM2344	NA_CCM2344_Ct_vert	CCM2344		vertebralis	superciliaris−E (N)	superciliaris	NT	Kakadu fire plot 66B	−13.2228	132.8513	OQ091845
CCM2357	NA_CCM2357_Ct_inor	CCM2357		inornatus	superciliaris−E (N)	superciliaris	NT	Kakadu fire plot 62B	−13.0941	132.9921	OQ091846
CCM2365	NA_CCM2365_Ct_inor	CCM2365		inornatus	superciliaris−E (N)	superciliaris	NT	Kakadu fire plot 153	−12.9947	132.8831	OQ091847
CCM2366	NA_CCM2366_Ct_inor	CCM2366		inornatus	superciliaris−E (N)	superciliaris	NT	Kakadu fire plot 153	−12.9947	132.8831	OQ091848
CCM2370	NA_CCM2370_Ct_inor	CCM2370		inornatus	superciliaris−E (N)	superciliaris	NT	Kakadu fire plot 153	−12.9947	132.8831	OQ091849
CCM2386	NA_CCM2386_Ct_inor	CCM2386		inornatus	superciliaris−E (N)	superciliaris	NT	Kakadu fire plot 121	−12.8442	132.9877	OQ091850
CCM2390	NA_CCM2390_Ct_inor	CCM2390		inornatus	superciliaris−E (N)	superciliaris	NT	Kakadu fire plot 121	−12.8442	132.9877	OQ091851
CCM2391	NA_CCM2391_Ct_inor	CCM2391		inornatus	superciliaris−E (N)	superciliaris	NT	Kakadu fire plot 151	−12.8442	132.9877	OQ091852
CCM2398	NA_CCM2398_Ct_inor	CCM2398		inornatus	superciliaris−E (N)	superciliaris	NT	Kakadu fire plot 121B	−12.8443	132.9866	OQ091853
CCM2399	NA_CCM2399_Ct_inor	CCM2399		inornatus	superciliaris−E (N)	superciliaris	NT	Kakadu fire plot 121B	−12.8443	132.9866	OQ091854
CCM2494	NA_CCM2494_Ct_vert	CCM2494		vertebralis	superciliaris−E (N)	superciliaris	NT	Kakadu fire plot 133B	−13.7441	132.6244	OQ091855
CCM2497	NA_CCM2497_Ct_spal	CCM2497		spaldingi	spaldingi−NE	spaldingi	NT	Kakadu fire plot 133B	−13.7441	132.6244	OQ091856
CCM2499	NA_CCM2499_Ct_vert	CCM2499		vertebralis	superciliaris−E (N)	superciliaris	NT	Kakadu fire plot 128	−13.6363	132.6057	OQ091857
CCM2518	NA_CCM2518_Ct_vert	CCM2518		vertebralis	superciliaris−E (N)	superciliaris	NT	Kakadu fire plot 26	−13.3518	132.4723	OQ091858
CCM2523	NA_CCM2523_Ct_vert	CCM2523		vertebralis	superciliaris−E (N)	superciliaris	NT	Kakadu fire plot 26B	−13.3530	132.4748	OQ091859
CCM2564	NA_CCM2564_Ct_arnh	CCM2564		arnhemensis	superciliaris−E (N)	superciliaris	NT	Kakadu fire plot 126	−12.9976	132.9327	OQ091860
CCM2565	NA_CCM2565_Ct_vert	CCM2565		vertebralis	superciliaris−E (N)	superciliaris	NT	Kakadu fire plot 126	−12.9976	132.9327	OQ091861
CCM2570	NA_CCM2570_Ct_arnh	CCM2570		arnhemensis	superciliaris−E (N)	superciliaris	NT	Kakadu fire plot 140	−12.8615	132.9794	OQ091862
CCM2571	NA_CCM2571_Ct_arnh	CCM2571		arnhemensis	superciliaris−E (N)	superciliaris	NT	Kakadu fire plot 140	−12.8615	132.9794	OQ091863
CCM2831	NA_CCM2831_Ct_inor	CCM2831		inornatus	inornatus−N	inornatus	WA	Roy Creek Mornington	−17.5262	126.1998	OQ091864
CCM2846	NA_CCM2846_Ct_robu	CCM2846		robustus	robustus−NW	robustus	WA	Marion driveway Tablelands	−17.2270	126.8770	OQ091865

Voucher or Tissue Sample	Sample ID	Tissue ID	Alternative ID	Original Taxon	OTU	Putative Taxon	State or Territory	Location	Latitude	Longitude	GenBank Accession
CCM2855	NA_CCM2855_Ct_inor	CCM2855		inornatus	inornatus−N	inornatus	WA	Chamberlain Valley	−17.1441	127.3370	OQ091866
CCM2866	NA_CCM2866_Ct_robu	CCM2866		robustus	robustus−NW	robustus	WA	Bluebush SV08 Mornington	−17.5543	126.1646	OQ091867
CCM3004	NA_CCM3004_Ct_robu	CCM3004		robustus	superciliaris−W	superciliaris	NT	Calcite Flow Camp	−16.0503	130.4021	OQ091868
CCM3568	NA_CCM3568_Ct_inor	CCM3568		inornatus	inornatus−N	inornatus	NT	Surprise01 Pungalina	−16.7928	137.2490	OQ091869
CCM3569	NA_CCM3569_Ct_inor	CCM3569		inornatus	inornatus−N	inornatus	NT	Surprise02 Pungalina	−16.7980	137.2500	OQ091870
CCM3717	NA_CCM3717_Ct_inor	CCM3717		inornatus	inornatus−N	inornatus	NT	Groote Eyland	−13.8487	136.5268	OQ091871
CCM3739	NA_CCM3739_Ct_inor	CCM3739		inornatus	inornatus−N	inornatus	NT	Groote Eyland	−13.9058	136.8206	OQ091872
CCM3820	NA_CCM3820_Ct_bore	CCM3820		borealis	robustus−NW	robustus	NT	Kapalga Knp	−12.6476	132.3601	OQ091873
CCM3869	NA_CCM3869_Ct_inor	CCM3869		inornatus	superciliaris−E (N)	superciliaris	NT	Ngangkan	−12.5539	134.0290	OQ091874
CCM3918	NA_CCM3918_Ct_inor	CCM3918		inornatus	superciliaris−E (N)	superciliaris	NT	Kamarrkawarn	−12.6952	134.0571	OQ091875
CCM3923	NA_CCM3923_Ct_inor	CCM3923		inornatus	superciliaris−E (N)	superciliaris	NT	Kamarrkawarn	−12.6079	134.0255	OQ091876
CCM4006	NA_CCM4006_Ct_bore	CCM4006		borealis	robustus−NW	robustus	NT	Garig Gunak Barlu National Park	−11.1576	132.3423	OQ091877
CCM4016	NA_CCM4016_Ct_quir	CCM4016		quirinus	spaldingi−NE	spaldingi	NT	Inglis Islands	−11.9779	136.3052	OQ091878
CCM4018	NA_CCM4018_Ct_inor	CCM4018		inornatus	inornatus−N	inornatus	NT	Inglis Islands	−11.9765	136.3020	OQ091879
CCM4039	NA_CCM4039_Ct_inor	CCM4039		inornatus	inornatus−N	inornatus	NT	Drysdale Islands	−11.7076	135.9724	OQ091880
CCM4044	NA_CCM4044_Ct_robu	CCM4044		robustus	robustus−NW	robustus	NT	Kamarrkawarn	−12.6434	134.0616	OQ091881
CUMV14589	CUMV_14589_Ct_hele	DLR0036		helenae	inornatus−S	inornatus	WA	Lorna Glen Stn	−26.2260	121.5575	OQ091787
CUMV14590	CUMV_14590_Ct_hele	DLR0119		helenae	inornatus−S	inornatus	WA	Lorna Glen Stn	−26.2260	121.5575	OQ091788
CUMV14591	CUMV_14591_Ct_hele	DLR0134		helenae	inornatus−S	inornatus	WA	Lorna Glen Stn	−26.2260	121.5575	OQ091789
CUMV14593	CUMV_14593_Ct_hele	DLR0093		helenae	inornatus−S	inornatus	WA	Lorna Glen Stn	−26.2260	121.5575	OQ091790
CUMV14594	CUMV_14594_Ct_hele	DLR0116		helenae	inornatus−S	inornatus	WA	Lorna Glen Stn	−26.2260	121.5575	OQ091791
CUMV14595	CUMV_14595_Ct_hele	DLR0132		helenae	inornatus−S	inornatus	WA	Lorna Glen Stn	−26.2260	121.5575	OQ091792
CUMV14596	CUMV_14596_Ct_hele	DLR0179		helenae	inornatus−S	inornatus	WA	Lorna Glen Stn	−26.2260	121.5575	OQ091793
CUMV14597	CUMV_14597_Ct_hele	DLR0070		helenae	inornatus−S	inornatus	WA	Lorna Glen Stn	−26.2260	121.5575	OQ091794
CUMV14598	CUMV_14598_Ct_hele	DLR0090		helenae	inornatus−S	inornatus	WA	Lorna Glen Stn	−26.2260	121.5575	OQ091795
CUMV14599	CUMV_14599_Ct_hele	DLR0130		helenae	inornatus−S	inornatus	WA	Lorna Glen Stn	−26.2260	121.5575	OQ091796
CUMV14600	CUMV_14600_Ct_hele	DLR0049	HELEDLR0049	helenae	inornatus−S	inornatus	WA	Lorna Glen Stn	−26.2260	121.5575	HQ332285
CUMV14601	CUMV_14601_Ct_hele	DLR0141		helenae	inornatus−S	inornatus	WA	Lorna Glen Stn	−26.2260	121.5575	OQ091797
CUMV14602	CUMV_14602_Ct_hele	DLR0193	HELEDLR0193	helenae	inornatus−S	inornatus	WA	Lorna Glen Stn	−26.2260	121.5575	HQ332286
CUMV14604	CUMV_14604_Ct_hele	DLR0223	HELEDLR0223	helenae	inornatus−S	inornatus	WA	Lorna Glen Stn	−26.2260	121.5575	HQ332287

Voucher or Tissue Sample	Sample ID	Tissue ID	Alternative ID	Original Taxon	OTU	Putative Taxon	State or Territory	Location	Latitude	Longitude	GenBank Accession
CUMV14605	CUMV_14605_Ct_hele	DLR0234	HELEDLR0234	helenae	inornatus−S	inornatus	WA	Lorna Glen Stn	−26.2260	121.5575	HQ332288
CUMV14606	CUMV_14606_Ct_hele	DLR0563	HELEDLR0563	helenae	inornatus−S	inornatus	WA	Yamarna Stn	−28.1330	123.8667	KJ505414
CUMV14608	CUMV_14608_Ct_hele	DLR0356	HELEDLR0356	helenae	inornatus−S	inornatus	WA	Yamarna Stn	−28.1330	123.8667	KJ505410
CUMV14609	CUMV_14609_Ct_hele	DLR0363	HELEDLR0363	helenae	inornatus−S	inornatus	WA	Yamarna Stn	−28.1330	123.8667	KJ505411
CUMV14610	CUMV_14610_Ct_hele	DLR0366	HELEDLR0366	helenae	inornatus−S	inornatus	WA	Yamarna Stn	−28.1330	123.8667	KJ505412
CUMV14611	CUMV_14611_Ct_hele	DLR0278	HELEDLR0278	helenae	inornatus−S	inornatus	WA	Yamarna Stn	−28.1330	123.8667	KJ505409
CUMV14612	CUMV_14612_Ct_hele	DLR0376	HELEDLR0376	helenae	inornatus−S	inornatus	WA	Lake Mason Stn	−27.5860	119.5208	KJ505413
CUMV14655	CUMV_14655_Ct_hele	DLR0592		helenae	inornatus−S	inornatus	WA	Yamarna Stn	−28.1330	123.8667	OQ091798
CUMV14656	CUMV_14656_Ct_hele	DLR0605		helenae	inornatus−S	inornatus	WA	Yamarna Stn	−28.1330	123.8667	OQ091799
CUMV14657	CUMV_14657_Ct_hele	DLR0621		helenae	inornatus−S	inornatus	WA	Yamarna Stn	−28.1330	123.8667	OQ091800
CUMV14663	CUMV_14663_Ct_hele	DLR0652	HELEDLR0652	helenae	inornatus−S	inornatus	WA	Yamarna Stn	−28.1330	123.8667	KJ505415
DLR0625	NA_DLR0625_Ct_hele	DLR0625		helenae	inornatus−S	inornatus	WA	Yamarna Stn	−28.1330	123.8667	OQ091882
ERPQ31307	NA_LATESP034_Ct_late	P−034 LAT	LATESP034	lateralis	lateralis	lateralis	NA	NA	NA	NA	KJ505500
ERPQ31340	NA_ROBUSP049_Ct_robu	P−049 ROB	ROBUSP049	robustus	spaldingi−NE	spaldingi	NA	NA	NA	NA	KJ505662
ERPQ31348	NA_NULLSP042_Ct_null	P−042 NUL	NULLSP042	nullum	inornatus−S	inornatus	NA	NA	NA	NA	KJ505026
ERPQ31349	NA_SPALSP056_Ct_spal	P−056 SPL	SPALSP056	spaldingi	spaldingi−NE	spaldingi	NA	NA	NA	NA	KJ505810
NMVD67682	NMVD_67682_Ct_saxa	ABTC09987	SAXAA09987	saxatilis	inornatus−S	inornatus	NT	Ormiston	−23.7000	132.7000	KJ505677
NMVD67683	NMVD_67683_Ct_saxa	ABTC09988		saxatilis	inornatus−S	inornatus	NT	Ormiston	−23.7000	132.7000	KJ505678
NMVD67969	NMVD_67969_Ct_hele	ABTC10043	HELEA10043	helenae	inornatus−S	inornatus	NT	22 km W Erldunda Roadhouse	−25.0000	133.2000	KJ505393
NTMR13503	NTMR_13503_Ct_saxa	ABTC28062	SAXAA28062	saxatilis	superciliaris−W	superciliaris	NT	Victoria R Gregory NP	−16.8230	130.4200	KJ505688
NTMR13838	NTMR_13838_Ct_bore	ABTC28390	BOREA28390	borealis	robustus−NW	robustus	NT	Kakadu NP	−13.0300	132.4300	KJ505262
NTMR16340	NTMR_16340_Ct_inor	ABTC28499	INORA28499	inornatus	superciliaris−E (S)	superciliaris	NT	Wave Hill Stn	−17.3000	131.0000	KJ505486
NTMR17738	NTMR_17738_Ct_cogg	ABTC29190	COGGA29190	coggeri	robustus−TE	robustus	NT	Jabiluka Project Area	−12.5000	132.8500	KJ504999
NTMR17739	NTMR_17739_Ct_bore	ABTC29191		borealis	robustus−NW	robustus	NT	Casuarina Coastal Reserve	−12.3700	130.8500	OQ091884
NTMR18323	NTMR_18323_Ct_robu	ABTC30302		robustus	robustus−NW	robustus	NT	Willeroo	−15.1608	131.6692	OQ091885
NTMR20243	NTMR_20243_Ct_saxa	ABTC29237	SAXAA29237	saxatilis	superciliaris−E (S)	superciliaris	NT	20 km N Carpentaria Hwy	NA	NA	KJ505690
NTMR20378	NTMR_20378_Ct_robu	ABTC29172	ROBUA29172	robustus	robustus−TE	robustus	NT	Fogg Dam	−12.5500	131.3000	KJ505620
NTMR20628	NTMR_20628_Ct_saxa	ABTC29398	SAXAA29398	saxatilis	inornatus−S	inornatus	NT	Finke Gorge NP	−24.1000	132.7000	KJ505691
NTMR20687	NTMR_20687_Ct_robu	ABTC28421	ROBUA28421	inornatus	inornatus−N	inornatus	NT	Mt. Bundy Stn	−13.3000	131.1000	KJ505619
NTMR20846	NTMR_20846_Ct_saxa	ABTC28173	SAXAA28173	saxatilis	superciliaris−E (N)	superciliaris	NT	Kakadu	−12.9000	132.6700	KJ505689

Voucher or Tissue Sample	Sample ID	Tissue ID	Alternative ID	Original Taxon	OTU	Putative Taxon	State or Territory	Location	Latitude	Longitude	GenBank Accession
NTMR21670	NTMR_21670_Ct_spal	ABTC29600		spaldingi	spaldingi−NE	spaldingi	NT	10 km N Larrimah	−15.5330	133.1830	OQ091886
NTMR22166	NTMR_22166_Ct_robu	ABTC29697		robustus	robustus−TE	robustus	NT	Litchfield NP	−13.2647	130.9633	OQ091887
NTMR22167	NTMR_22167_Ct_bore	ABTC29698	BOREA29698	borealis	robustus−NW	robustus	NT	Litchfield NP	−13.2650	130.9622	KJ505263
NTMR22175	NTMR_22175_Ct_robu	ABTC29704	ROBUA29704	robustus	robustus−NW	robustus	NT	Litchfield NP	−13.2650	130.9614	KJ505622
NTMR22185	NTMR_22185_Ct_inor	ABTC29709	INORA29709	inornatus	superciliaris−E (S)	superciliaris	NT	Litchfield NP	−13.4100	130.8972	KJ505487
NTMR22298	NTMR_22298_Ct_spal	ABTC30415		spaldingi	spaldingi−NE	spaldingi	NT	Limmen Gate NP	−15.4833	135.4122	OQ091888
NTMR22299	NTMR_22299_Ct_spal	ABTC30414		spaldingi	spaldingi−NE	spaldingi	NT	7 km N Nathan River Station Limmen Gate NP	−15.5331	135.4081	OQ091889
NTMR22301	NTMR_22301_Ct_robu	ABTC30416	ROBUA30416	robustus	robustus−NW	robustus	NT	7 km N Nathan R Stn Limmen Gate NP	−15.5330	135.4081	KJ505625
NTMR22325	NTMR_22325_Ct_spal	ABTC30410		spaldingi	spaldingi−NE	spaldingi	NT	Tawallah Creek Limmen Gate NP	−16.0158	135.6669	OQ091890
NTMR22432	NTMR_22432_Ct_saxa	ABTC30404	SAXAA30404	saxatilis	superciliaris−E (S)	superciliaris	NT	Nathan River Stn Limmen Gate NP	−15.5780	135.4297	KJ505692
NTMR22620	NTMR_22620_Ct_robu	ABTC29870		robustus	robustus−NW	robustus	NT	Long Billabong Roper River	−15.3067	135.3408	OQ091891
NTMR22621	NTMR_22621_Ct_spal	ABTC29871		spaldingi	spaldingi−NE	spaldingi	NT	Long Billabong Roper River	−15.3067	135.3408	OQ091892
NTMR22622	NTMR_22622_Ct_spal	ABTC29872		spaldingi	spaldingi−NE	spaldingi	NT	Long Billabong Roper River	−15.3067	135.3408	OQ091893
NTMR22934	NTMR_22934_Ct_robu	ABTC30181	ROBUA30181	robustus	robustus−NW	robustus	NT	Spirit Hills Keep R	−15.3070	129.1583	KJ505623
NTMR22935	NTMR_22935_Ct_robu	ABTC30182	ROBUA30182	robustus	robustus−NW	robustus	NT	Spirit Hills Keep R	−15.3070	129.1583	KJ505624
NTMR23004	NTMR_23004_Ct_bore	ABTC30072		borealis	robustus−NW	robustus	NT	creek SW Pickertaramoor	−11.7822	130.7744	OQ091894
NTMR23777	NTMR_23777_Ct_robu	ABTC30599	ROBUA30599	robustus	robustus−NW	robustus	NT	Wickham R Gregory NP	−16.8190	130.1742	KJ505626
NTMR23791	NTMR_23791_Ct_robu	ABTC30603	ROBUA30603	robustus	robustus−NW	robustus	NT	Wickham R Gregory NP	−16.8530	130.1856	KJ505627
NTMR23946	NTMR_23946_Ct_robu	ABTC30691	ROBUA30691	robustus	robustus−TE	robustus	NT	Ramingining area Arafura Swamp	−12.1700	134.9686	KJ505628
NTMR23960	NTMR_23960_Ct_robu	ABTC30705		robustus	robustus−NW	robustus	NT	Ramingining area Arafura Swamp	−12.2175	134.9847	OQ091895
NTMR25983	NTMR_25983_Ct_spal	ABTC70692		spaldingi	superciliaris−W	superciliaris	NT	Jasper Gorge Gregory National Park	−16.0378	130.7900	OQ091896
NTMR26117	NTMR_26117_Ct_inor	ABTC72530	INORA72530	inornatus	superciliaris−E (N)	superciliaris	NT	Upper Reaches Arnhemland Plateau	−13.2830	133.5333	KJ505489
PMO214	NA_PMO214_Ct_inor	PMO214		inornatus	superciliaris−W	superciliaris	WA	Near Broome tip	−17.8971	122.2396	OQ091883
QMJ48384	QM_48384_Ct_robu	ABTC16215	ROBUA16215	robustus	spaldingi−S	spaldingi	QLD	Winton	−22.4000	143.0000	KJ505617
QMJ82086	QM_82086_Ct_spal	ABTC105811	SPALA105811	spaldingi	spaldingi−NE	spaldingi	QLD	Lion's Den Pub near Black Mountain	−15.8000	145.2500	KJ506016
QMJ86746	QM_86746_Ct_capr	ABTC105818	CAPRIA105818	capricorni	inornatus−S	inornatus	QLD	Calabah Station	−27.0300	146.5500	KJ505882
QMJ87459	QM_87459_Ct_spal	ABTC105821	SPALA105821	spaldingi	spaldingi−NE	spaldingi	QLD	Porcupine Gorge NP	−20.3420	144.4597	KJ506017
QMJ87533	QM_87533_Ct_spal	ABTC105822	SPALA105822	spaldingi	spaldingi−CY	spaldingi	QLD	Mabuiag Island	−9.9460	142.1969	KJ506018
QMJ87556	QM_87556_Ct_spal	ABTC105823		spaldingi	spaldingi−CY	spaldingi	QLD	Moa Island	−10.1500	142.2500	KJ506052
SAMAR19910	SAMR_19910_Ct_robu	ABTC53561	ROBUA53561	robustus	spaldingi−S	spaldingi	SA	Barber Hill Gawler Rngs	−32.2000	135.1167	KJ505638

Voucher or Tissue Sample	Sample ID	Tissue ID	Alternative ID	Original Taxon	OTU	Putative Taxon	State or Territory	Location	Latitude	Longitude	GenBank Accession
SAMAR21493	SAMR_21493_Ct_robu	ABTC53607	ROBUA53607	robustus	spaldingi−S	spaldingi	SA	Coorong NP	−35.5670	138.9667	KJ505639
SAMAR22765	SAMR_22765_Ct_brac	P−071 BRA	BRACSP071	brachyonyx	inornatus−S	inornatus	SA	16 km SW Waikerie	−34.2000	140.0000	KJ505274
SAMAR27413	SAMR_27413_Ct_robu	ABTC53726	ROBUA53726	robustus	spaldingi−S	spaldingi	SA	4 km W of Palmer	−34.8420	139.1333	KJ505640
SAMAR27414	SAMR_27414_Ct_robu	ABTC53727		robustus	spaldingi−S	spaldingi	SA	4 km W of Palmer	−34.8420	139.1333	KJ505641
SAMAR28213	SAMR_28213_Ct_saxa	ABTC53823	SAXAA53823	saxatilis	inornatus−S	inornatus	SA	Dalhousie Ruins	−26.5170	135.4667	KJ505704
SAMAR28536	SAMR_28536_Ct_robu	ABTC53759	ROBUA53759	robustus	spaldingi−S	spaldingi	SA	Fresh Wells 67 km NW Iron Knob	−32.4000	136.5333	KJ505642
SAMAR29707	SAMR_29707_Ct_brac	ABTC53772	BRACA53772	brachyonyx	inornatus−S	inornatus	SA	Danggali CP	−33.2000	140.9167	KJ505269
SAMAR32203	SAMR_32203_Ct_hele	ABTC64253	HELEA64253	helenae	inornatus−S	inornatus	SA	50 km SW Halinor Lake SA	−29.5320	130.1394	KJ505407
SAMAR32276	SAMR_32276_Ct_hele	ABTC64312	HELEA64312	helenae	inornatus−S	inornatus	SA	50 km SW Halinor Lake SA	−29.5250	130.1500	KJ505408
SAMAR32932	SAMR_32932_Ct_robu	ABTC14912	ROBUA14912	robustus	spaldingi−S	spaldingi	SA	16 km W Penola	−37.3670	140.6500	KJ505616
SAMAR33525	SAMR_33525_Ct_robu	ABTC03662	ROBUA03662	robustus	spaldingi−S	spaldingi	NSW	Lancoona HS	−33.3670	145.8833	KJ505605
SAMAR33526	SAMR_33526_Ct_robu	ABTC03663	ROBUA03663	robustus	spaldingi−S	spaldingi	NSW	Lancoona HS	−33.3670	145.8833	KJ505606
SAMAR33559	SAMR_33559_Ct_robu	ABTC03709	ROBUA03709	robustus	spaldingi−S	spaldingi	NSW	11 km W Narrabri	−30.2500	149.8500	KJ505607
SAMAR33560	SAMR_33560_Ct_robu	ABTC03710	ROBUA03710	robustus	spaldingi−S	spaldingi	NSW	11 km W Narrabri	−30.2500	149.8500	KJ505608
SAMAR33695	SAMR_33695_Ct_robu	ABTC03961	ROBUA03961	robustus	spaldingi−S	spaldingi	NSW	Yamba tip	−29.4330	153.3667	KJ505612
SAMAR33706	SAMR_33706_Ct_robu	ABTC03982	ROBUA03982	robustus	spaldingi−S	spaldingi	NSW	Cairncross SF	−31.3830	152.6000	KJ505613
SAMAR33707	SAMR_33707_Ct_robu	ABTC04017	ROBUA04017	robustus	spaldingi−S	spaldingi	NSW	25 km N Bathurst	−33.2000	149.6667	KJ505614
SAMAR33876	SAMR_33876_Ct_robu	ABTC03826	ROBUA03826	robustus	spaldingi−S	spaldingi	QLD	Willowbank Caravan Pk	−27.6170	152.7833	KJ505609
SAMAR33877	SAMR_33877_Ct_robu	ABTC03827	ROBUA03827	robustus	spaldingi−S	spaldingi	QLD	Willowbank Caravan Pk	−27.6170	152.7833	KJ505610
SAMAR34123	SAMR_34123_Ct_hill	ABTC11807	HILLA11807	inornatus	inornatus−N	inornatus	NT	Jabiru East	−12.6500	132.8833	KJ505927
SAMAR34166	SAMR_34166_Ct_inor	ABTC11862	INORA11862	inornatus	superciliaris−E (N)	superciliaris	NT	El Sharana Mine Site	−13.5170	132.5000	KJ505484
SAMAR34180	SAMAR_34180_Ct_bore	ABTC11881		borealis	superciliaris−E (S)	superciliaris	NT	Tanumbirini Station	−16.4500	134.6167	OQ091897
SAMAR34202	SAMAR_34202_Ct_spal	ABTC11919		spaldingi	spaldingi−NE	spaldingi	QLD	Westmoreland Station	−17.3333	138.2500	OQ091898
SAMAR34261	SAMAR_34261_Ct_late	ABTC11988		lateralis	lateralis	lateralis	QLD	6 km E Camooweel	−19.9167	138.1667	OQ091899
SAMAR35032	SAMR_35032_Ct_brac	ABTC16838	BRACA16838	brachyonyx	inornatus−S	inornatus	SA	12 km S Bloodweed Bore	−26.9500	140.9500	KJ505265
SAMAR35036	SAMR_35036_Ct_brac	ABTC23999		brachyonyx	inornatus−S	inornatus	SA	70 km E Moomba	−28.1170	140.8667	KJ505266
SAMAR35964	SAMR_35964_Ct_hele	ABTC00445	HELEA00445	helenae	inornatus−S	inornatus	SA	Dalhousie Ruins SA	−26.5170	135.4667	KJ505392
SAMAR35965	SAMR_35965_Ct_brac	ABTC00446	BRACA00446	brachyonyx	inornatus−S	inornatus	SA	Pedirka	−26.6500	135.2000	KJ505264
SAMAR36246	SAMR_36246_Ct_inor	ABTC56691	INORA56691	superciliaris	superciliaris−E (S)	superciliaris	NT	Dunmara	−16.6830	133.4167	KJ505488
SAMAR36252	SAMR_36252_Ct_brac	ABTC56697	BRACA56697	brachyonyx	inornatus−S	inornatus	SA	Billiat CP	−34.9000	140.4500	KJ505270

Voucher or Tissue Sample	Sample ID	Tissue ID	Alternative ID	Original Taxon	OTU	Putative Taxon	State or Territory	Location	Latitude	Longitude	GenBank Accession
SAMAR36313	SAMR_36313_Ct_robu	ABTC56710	ROBUA56710	robustus	spaldingi−S	spaldingi	SA	Telowie Gorge	−33.0170	138.1000	KJ505643
SAMAR36349	SAMR_36349_Ct_brac	ABTC56718		brachyonyx	inornatus−S	inornatus	SA	20 km ESE Kalladeina Bore	−27.7170	139.3167	KJ505271
SAMAR36603	SAMR_36603_Ct_robu	ABTC17100	ROBUA17100	robustus	spaldingi−S	spaldingi	NSW	Esdale NW of Canberra	−35.0830	148.9167	KJ505618
SAMAR37975	SAMR_37975_Ct_robu	ABTC57046	ROBUA57046	robustus	spaldingi−S	spaldingi	SA	Burra Ck Gorge Res 17 km SE Burra	−33.8330	139.0167	KJ505644
SAMAR38760	SAMR_38760_Ct_saxa	ABTC12181	SAXAA12181	saxatilis	superciliaris−E (S)	superciliaris	NT	One Tank Hill 2 km E Tennant Creek	−19.6500	134.2000	KJ505684
SAMAR38761	SAMR_38761_Ct_saxa	ABTC12182	SAXAA12182	saxatilis	superciliaris−E (S)	superciliaris	NT	One Tank Hill 2 km E Tennant Creek	−19.6500	134.2000	KJ505685
SAMAR38762	SAMR_38762_Ct_saxa	ABTC12183	SAXAA12183	saxatilis	superciliaris−E (S)	superciliaris	NT	One Tank Hill 2 km E Tennant Creek	−19.6500	134.2000	KJ505686
SAMAR38766	SAMR_38766_Ct_saxa	ABTC12104	SAXAA12104	saxatilis	superciliaris−E (S)	superciliaris	NT	Tennant Creek	−19.6500	134.1833	KJ505683
SAMAR38776	SAMR_38776_Ct_saxa	ABTC12005	SAXAA12005	saxatilis	superciliaris−E (S)	superciliaris	NT	Phillip Creek Mission (abandoned)	−19.2830	134.2167	KJ505679
SAMAR38808	SAMR_38808_Ct_saxa	ABTC12038	SAXAA12038	saxatilis	inornatus−S	inornatus	NT	Barrow Creek	−21.5330	133.8833	KJ505680
SAMAR38810	SAMR_38810_Ct_saxa	ABTC12040		saxatilis	inornatus−S	inornatus	NT	Barrow Creek	−21.5330	133.8833	KJ505681
SAMAR38811	SAMR_38811_Ct_saxa	ABTC12041	SAXAA12041	saxatilis	inornatus−S	inornatus	NT	Barrow Creek	−21.5330	133.8833	KJ505682
SAMAR39480	SAMR_39480_Ct_robu	ABTC34469	ROBUA34469	robustus	spaldingi−S	spaldingi	SA	20 km NE of Murray Bridge	−35.0220	139.4639	KJ505629
SAMAR40718	SAMR_40718_Ct_robu	ABTC57450		robustus	spaldingi−S	spaldingi	SA	Cooks North Middleback Rngs	−33.1670	137.1333	KJ505645
SAMAR41330	SAMR_41330_Ct_brac	ABTC39994	BRACA39994	brachyonyx	inornatus−S	inornatus	SA	Oakbank Out Stn	−33.1280	140.6056	KJ505268
SAMAR41804	SAMR_41804_Ct_robu	ABTC57559	ROBUA57559	robustus	spaldingi−S	spaldingi	SA	Dutchmans Stern CP	−32.2920	137.9500	KJ505646
SAMAR41805	SAMR_41805_Ct_robu	ABTC57560	ROBUA57560	robustus	spaldingi−S	spaldingi	SA	Dutchmans Stern CP	−32.2920	137.9500	KJ505647
SAMAR42687	SAMAR_42687_Ct_late	ABTC8937		lateralis	lateralis	lateralis	QLD	7 km NE Mt Isa Telecom Repeator Station	−20.7167	139.5500	OQ091900
SAMAR42758	SAMR_42758_Ct_late	ABTC09000	LATEA09000	lateralis	lateralis	lateralis	QLD	S of Winton	−22.6670	142.9333	KJ505499
SAMAR42768	SAMAR_42768_Ct_late	ABTC9010		lateralis	lateralis	lateralis	QLD	Glen Kyree Station S Winton	−22.9667	142.9000	OQ091901
SAMAR42837	SAMAR_42837_Ct_late	ABTC9082		lateralis	lateralis	lateralis	QLD	43 Km N Diamantina Station	−23.5167	141.4000	OQ091902
SAMAR42918	SAMR_42918_Ct_saxa	ABTC09164	SAXAA09164	saxatilis	inornatus−S	inornatus	QLD	71 Km W of Windorah	−25.3670	141.9333	KJ505676
SAMAR44367	SAMR_44367_Ct_hele	ABTC41506	HELEA41506	helenae	inornatus−S	inornatus	SA	12.5 km ENE Mt Cooparinna	−26.3460	130.0900	KJ505400
SAMAR44377	SAMR_44377_Ct_hele	ABTC41498	HELEA41498	helenae	inornatus−S	inornatus	SA	20 km NE Mt Cooparinna	−26.2970	130.1517	KJ505399
SAMAR44832	SAMR_44832_Ct_saxa	ABTC41666	SAXAA41666	saxatilis	inornatus−S	inornatus	SA	3 km NE Mt Woodroffe	−26.2920	131.7864	KJ505698
SAMAR44833	SAMR_44833_Ct_saxa	ABTC41667	SAXAA41667	saxatilis	inornatus−S	inornatus	SA	3 km NE Mt Woodroffe	−26.2920	131.7864	KJ505699
SAMAR44879	SAMR_44879_Ct_saxa	ABTC41651	SAXAA41651	saxatilis	inornatus−S	inornatus	SA	4 km SSW Mt Cuthbert	−26.1360	132.0667	KJ505696
SAMAR44887	SAMR_44887_Ct_saxa	ABTC41661	SAXAA41661	saxatilis	inornatus−S	inornatus	SA	4 km SSW Mt Cuthbert	−26.1360	132.0667	KJ505697
SAMAR45215	SAMR_45215_Ct_brac	ABTC58077	BRACA58077	brachyonyx	inornatus−S	inornatus	SA	N edge Peebinga CP	−34.9580	140.8333	KJ505272
SAMAR45314	SAMR_45314_Ct_robu	ABTC58150	ROBUA58150	robustus	spaldingi−S	spaldingi	SA	Lincoln Gap Stn	−32.5800	137.5583	KJ505648

Voucher or Tissue Sample	Sample ID	Tissue ID	Alternative ID	Original Taxon	OTU	Putative Taxon	State or Territory	Location	Latitude	Longitude	GenBank Accession
SAMAR45480	SAMR_45480_Ct_saxa	ABTC41729	SAXAA41729	saxatilis	inornatus−S	inornatus	SA	9.7 km S Ampeinna Hills	−27.1570	131.1311	KJ505700
SAMAR45752	SAMR_45752_Ct_brac	ABTC35349		brachyonyx	inornatus−S	inornatus	SA	3.6 km E Bookacala Hill	−26.0280	140.1850	KJ505267
SAMAR46119	SAMR_46119_Ct_hele	ABTC41775	HELEA41775	helenae	inornatus−S	inornatus	SA	27 km NE Pipalyatjara	−26.0530	129.4081	KJ505401
SAMAR46196	SAMR_46196_Ct_robu	ABTC35545	ROBUA35545	robustus	spaldingi−S	spaldingi	SA	11.5 km SE Wares Peak	−29.6620	135.7656	KJ505630
SAMAR46204	SAMR_46204_Ct_robu	ABTC35548		robustus	spaldingi−S	spaldingi	SA	4 km N Halifax Hill	−29.6840	135.8153	KJ505631
SAMAR46880	SAMR_46880_Ct_saxa	ABTC36128	SAXAA36128	saxatilis	inornatus−S	inornatus	SA	11.1 km ESE Mt Goodiar Witjira NP	−26.6880	135.7167	KJ505695
SAMAR47028	SAMR_47028_Ct_robu	ABTC36230		robustus	spaldingi−S	spaldingi	SA	35 km ENE Kingston SE	−36.7380	140.2397	KJ505632
SAMAR48684	SAMR_48684_Ct_robu	ABTC58575	ROBUA58575	robustus	spaldingi−S	spaldingi	SA	Moonaree Stn	−31.7050	135.5256	KJ505030
SAMAR48710	SAMR_48710_Ct_hele	ABTC41974	HELEA41974	helenae	inornatus−S	inornatus	SA	4 km W Mt Lindsay SA	−27.0290	129.8392	KJ505402
SAMAR48713	SAMR_48713_Ct_hele	ABTC41977	HELEA41977	helenae	inornatus−S	inornatus	SA	4 km W Mt Lindsay SA	−27.0290	129.8392	KJ505403
SAMAR48840	SAMR_48840_Ct_robu	ABTC37113	ROBUA37113	robustus	spaldingi−S	spaldingi	SA	Arcoona Stn	−31.2810	136.5900	KJ505633
SAMAR48962	SAMR_48962_Ct_robu	ABTC37169	ROBUA37169	robustus	spaldingi−S	spaldingi	SA	Andamooka Stn	−30.7420	137.3136	KJ505634
SAMAR49609	SAMR_49609_Ct_robu	ABTC37540		robustus	spaldingi−S	spaldingi	SA	7 km NE Hatherleigh SA	−37.4380	140.3136	KJ505635
SAMAR50208	SAMR_50208_Ct_saxa	ABTC42206	SAXAA42206	saxatilis	inornatus−S	inornatus	SA	11.2 km SW Sentinel Hill	−26.1410	132.3592	KJ505701
SAMAR50209	SAMR_50209_Ct_saxa	ABTC42208	SAXAA42208	saxatilis	inornatus−S	inornatus	SA	11.2 km SW Sentinel Hill	−26.1410	132.3592	KJ505702
SAMAR50372	SAMR_50372_Ct_robu	ABTC38130	ROBUA38130	robustus	spaldingi−S	spaldingi	SA	1 km SE Mt Aroona	−30.5840	138.3689	KJ505636
SAMAR51089	SAMR_51089_Ct_saxa	ABTC13418	SAXAA13418	saxatilis	inornatus−S	inornatus	NT	Tennant Creek Dump	−19.6500	134.1833	KJ505687
SAMAR51093	SAMAR_51093_Ct_spal	ABTC13415		spaldingi	spaldingi−NE	spaldingi	NT	2 km S Elliott	−17.5667	133.5333	OQ091903
SAMAR51538	SAMR_51538_Ct_saxa	ABTC42345		saxatilis	inornatus−S	inornatus	SA	35 km ESE Amata	−26.2530	131.4803	KJ505703
SAMAR52303	SAMR_52303_Ct_robu	ABTC39414	ROBUA39414	robustus	spaldingi−S	spaldingi	SA	1.5 km S Camel Yard Spring	−30.6600	139.0911	KJ505637
SAMAR52956	SAMR_52956_Ct_robu	ABTC74190	ROBUA74190	robustus	spaldingi−S	spaldingi	SA	Arkaroola	−30.1190	139.4483	KJ505656
SAMAR53973	SAMR_53973_Ct_saxa	ABTC72676		saxatilis	inornatus−S	inornatus	NT	Barrow Creek channel just N town	−21.5000	133.9000	KJ505705
SAMAR53975	SAMR_53975_Ct_saxa	ABTC72677		saxatilis	superciliaris−E (S)	superciliaris	NT	Barrow Creek channel just N town	−21.5000	133.9000	KJ505706
SAMAR54008	SAMR_54008_Ct_saxa	ABTC72702	SAXAA72702	saxatilis	inornatus−S	inornatus	NT	2 km N Barrow Creek	−21.5060	133.8939	KJ505707
SAMAR54340	SAMR_54340_Ct_late	ABTC72759	LATEA72759	lateralis	lateralis	lateralis	QLD	East Leichardt Dam	−20.7750	139.7856	KJ505944
SAMAR54433	SAMAR_54433_Ct_late	ABTC72838		lateralis	lateralis	lateralis	QLD	21 km S Burke & Wills RH	−19.3947	140.2367	OQ091904
SAMAR54434	SAMR_54434_Ct_late	ABTC72839	LATEA72839	lateralis	lateralis	lateralis	QLD	21 km S Burke & Wills RH	−19.3950	140.2367	KJ505021
SAMAR54463	SAMAR_54463_Ct_late	ABTC72875		lateralis	lateralis	lateralis	QLD	Burke & Wills RH Dump	−19.2264	140.3481	OQ091905
SAMAR54485	SAMAR_54485_Ct_spal	ABTC72903		spaldingi	spaldingi−NE	spaldingi	QLD	22 km S Torrens Creek	−20.9858	145.0317	OQ091906
SAMAR55259	SAMAR_55259_Ct_late	ABTC82415		lateralis	lateralis	lateralis	QLD	Phosphate Hill Bloodwood site	−21.9131	140.0217	OQ091907

Voucher or Tissue Sample	Sample ID	Tissue ID	Alternative ID	Original Taxon	OTU	Putative Taxon	State or Territory	Location	Latitude	Longitude	GenBank Accession
SAMAR55340	SAMR_55340_Ct_brac	ABTC74639	BRACA74639	brachyonyx	inornatus−S	inornatus	SA	14 km ENE Gluepot HS	−33.7510	140.2747	KJ505273
SAMAR55675	SAMAR_55675_Ct_spal	ABTC76994		spaldingi	spaldingi−NE	spaldingi	QLD	40 km S Torrens Creek on Torrens Creek−Aramac Road	−21.0917	145.0044	OQ091908
SAMAR55725	SAMR_55725_Ct_spal	ABTC77041	SPALA77041	spaldingi	spaldingi−NE	spaldingi	QLD	33 km NNE Hughenden on Kennedy Developmental Rd	−20.6240	144.3997	KJ505038
SAMAR55731	SAMAR_55731_Ct_robu	ABTC77047		robustus	spaldingi−S	spaldingi	QLD	6.5 km E Julia Creek on Flinders Highway	−20.6553	141.7975	OQ091909
SAMAR55745	SAMR_55745_Ct_robu	ABTC77062	ROBUA77062	robustus	spaldingi−S	spaldingi	QLD	37 km SSE Julia Ck	−20.9750	141.8917	KJ505657
SAMAR55746	SAMR_55746_Ct_robu	ABTC77063	ROBUA77063	robustus	spaldingi−S	spaldingi	QLD	37 km SSE Julia Ck	−20.9750	141.8917	KJ505658
SAMAR55799	SAMAR_55799_Ct_late	ABTC77111		lateralis	eutaenius	cf. eutaenius	QLD	35 km E Mt Surprise on Gulf Developmental Road	−18.1283	144.6328	OQ091910
SAMAR55874	SAMR_55874_Ct_euta	ABTC77199	EUTAA77199	eutaenius	eutaenius	cf. eutaenius	QLD	Charters Towers	−20.0890	146.2525	KJ505009
SAMAR55880	SAMR_55880_Ct_robu	ABTC77204	ROBUA77204	robustus	spaldingi−S	spaldingi	QLD	69 km S Alpha on Alpha−Tambo Rd	−24.1980	146.5508	KJ505659
SAMAR55881	SAMR_55881_Ct_robu	ABTC77205	ROBUA77205	robustus	spaldingi−S	spaldingi	QLD	69 km S Alpha on Alpha−Tambo Rd	−24.1980	146.5508	KJ505660
SAMAR55891	SAMR_55891_Ct_robu	ABTC79480	ROBUA79480	robustus	spaldingi−S	spaldingi	QLD	Tambo Dump	−24.8600	146.2558	KJ505661
SAMAR62032	SAMR_62032_Ct_hele	ABTC91462	HELEA91462	helenae	inornatus−S	inornatus	WA	3.2 km N Pungkulpirri Waterhole Walter James Range	−24.6286	128.7556	KJ505920
SAMAR62119	SAMR_62119_Ct_hele	ABTC91688	HELEA91688	helenae	inornatus−S	inornatus	WA	Morgan Range 17.3 km ENE Blackstone	−25.9325	128.4411	KJ505924
SAMAR62198	SAMR_62198_Ct_hele	ABTC91549	HELEA91549	helenae	inornatus−S	inornatus	WA	Kutjuntari Rockhole	−24.8914	128.7692	KJ505921
SAMAR65339	SAMR_65339_Ct_late	ABTC113697		lateralis	lateralis	lateralis	QLD	23.5 km NE Alderley Homestead	−22.3850	139.8564	OQ091911
SAMAR65407	SAMR_65407_Ct_late	ABTC113882		lateralis	lateralis	lateralis	QLD	17.1 km W Noonbah Homestead	−24.1017	143.0175	OQ091912
SAMAR65416	SAMR_65416_Ct_late	ABTC113822		lateralis	lateralis	lateralis	QLD	3.7 km SSE Noonbah Homestead	−24.1361	143.2017	OQ091913
UMMZ242614	UMMZ_242614_Ct_hele	UMFS20505		helenae	inornatus−S	inornatus	WA	Dampier Downs Station	−18.1143	123.5507	OQ091914
UMMZ242616	UMMZ_242616_Ct_hele	UMFS20510		helenae	inornatus−S	inornatus	WA	Dampier Downs Station	−18.1143	123.5507	OQ091915
UMMZ242623	UMMZ_242623_Ct_hele	UMFS20603		helenae	inornatus−S	inornatus	WA	Stretch Lagoon	−19.6790	127.5867	OQ091916
UMMZ242624	UMMZ_242624_Ct_inor	UMFS20506		inornatus	superciliaris−W	superciliaris	WA	Dampier Downs Station	−18.0919	123.5694	OQ091917
UMMZ242625	UMMZ_242625_Ct_inor	UMFS20516		inornatus	superciliaris−W	superciliaris	WA	Dampier Downs Station	−18.1143	123.5507	OQ091918
UMMZ242629	UMMZ_242629_Ct_inor	UMFS20597		inornatus	superciliaris−W	superciliaris	WA	Carranya Station	−19.2425	127.7828	OQ091919
UMMZ242630	UMMZ_242630_Ct_inor	UMFS20599		inornatus	superciliaris−W	superciliaris	WA	Carranya Station	−19.2426	127.7806	OQ091920
WAMR084577	WAMR_084577_Ct_hele	WAMR084577	HELEW084577	helenae	inornatus−S	inornatus	WA	North Lake Throssell	−27.2500	124.4167	KJ505416
WAMR090719	WAMR_090719_Ct_hele	WAMR090719	HELEW090719	helenae	inornatus−S	inornatus	WA	Woodstock Station	−21.6100	119.0214	KJ505417
WAMR090727	WAMR_090727_Ct_hele	WAMR090727	HELEW090727	helenae	inornatus−S	inornatus	WA	Woodstock Station	−21.6100	119.0214	KJ505418
WAMR090839	WAMR_090839_Ct_saxa	WAMR090839	SAXAW090839	saxatilis	superciliaris−W	superciliaris	WA	Woodstock Station	−21.6170	119.0233	KJ505708
WAMR090862	WAMR_090862_Ct_hele	WAMR090862	HELEW090862	helenae	inornatus−S	inornatus	WA	Gallery Hill	−21.6680	119.0408	KJ505419
WAMR094929	WAMR_094929_Ct_hele	WAMR094929	HELEW094929	helenae	inornatus−S	inornatus	WA	Thompson Hills	−21.3330	124.7500	KJ505420

Voucher or Tissue Sample	Sample ID	Tissue ID	Alternative ID	Original Taxon	OTU	Putative Taxon	State or Territory	Location	Latitude	Longitude	GenBank Accession
WAMR097178	WAMR_097178_Ct_seve	P−054 SEV	SEVESP054	severus	inornatus−S	inornatus	WA	NA	−26.800	117.2000	KJ505807
WAMR099344	WAMR_099344_Ct_hele	WAMR099344	HELEW099344	helenae	inornatus−S	inornatus	WA	Woodstock Station	−21.612	118.9556	KJ505421
WAMR100648	WAMR_100648_Ct_saxa	WAMR100648	SAXAW100648	saxatilis	superciliaris−W	superciliaris	WA	Woodstock Station	−21.526	119.1492	KJ505709
WAMR100649	WAMR_100649_Ct_saxa	WAMR100649	SAXAW100649	saxatilis	superciliaris−W	superciliaris	WA	Woodstock Station	−21.526	119.1492	KJ505710
WAMR100697	WAMR_100697_Ct_hele	WAMR100697		helenae	inornatus−S	inornatus	WA	Woodstock Station	−21.609	119.0214	KJ505422
WAMR102145	WAMR_102145_Ct_saxa	WAMR102145		saxatilis	inornatus−S	inornatus	WA	Mt Windell	−22.603	118.4597	KJ505711
WAMR102423	WAMR_102423_Ct_saxa	WAMR102423	SAXAW102423	saxatilis	inornatus−S	inornatus	WA	Barlee Range Nature Reserve	−23.102	116.0078	KJ505712
WAMR102492	WAMR_102492_Ct_hele	WAMR102492	HELEW102492	helenae	inornatus−S	inornatus	WA	Barlee Range Nature Reserve	−23.044	115.8125	KJ505424
WAMR102671	WAMR_102671_Ct_hele	WAMR102671	HELEW102671	helenae	inornatus−S	inornatus	WA	Little Sandy Desert	−24.054	120.4067	KJ505425
WAMR102771	WAMR_102771_Ct_hele	WAMR102771	HELE102771	helenae	inornatus−S	inornatus	WA	Little Sandy Desert	−24.532	120.2911	KJ505426
WAMR104008	WAMR_104008_Ct_hele	WAMR104008	HELEW104008	helenae	inornatus−S	inornatus	WA	Woodstock Station	−21.671	119.0417	KJ505427
WAMR104069	WAMR_104069_Ct_hele	WAMR104069	HELEW104069	helenae	inornatus−S	inornatus	WA	Woodstock Station	−21.609	119.0214	KJ505428
WAMR104103	WAMR_104103_Ct_saxa	WAMR104103	SAXAW104103	saxatilis	superciliaris−W	superciliaris	WA	Woodstock Station	−21.596	119.0889	KJ505713
WAMR106093	WAMR_106093_Ct_saxa	WAMR106093	SAXAW106093	saxatilis	superciliaris−W	superciliaris	WA	Bluebell Island	−20.400	115.5167	KJ505714
WAMR108347	WAMR_108347_Ct_saxa	WAMR108347	SAXAW108347	saxatilis	superciliaris−W	superciliaris	WA	Onslow	−21.597	115.0603	KJ505715
WAMR108593	WAMR_108593_Ct_hele	WAMR108593	HELEW108593	helenae	inornatus−S	inornatus	WA	Pannawonica	−21.817	116.2333	KJ505429
WAMR108698	WAMR_108698_Ct_saxa	WAMR108698	SAXAW108698	saxatilis	superciliaris−W	superciliaris	WA	Cheerabun HS	−18.183	125.1167	KJ505716
WAMR108699	WAMR_108699_Ct_saxa	WAMR108699	SAXAW108699	saxatilis	superciliaris−W	superciliaris	WA	Cheerabun HS	−18.183	125.1167	KJ505717
WAMR108742	WAMR_108742_Ct_hele	WAMR108742	HELEW108742	helenae	inornatus−S	inornatus	WA	Banana Springs	−18.900	128.8000	KJ505430
WAMR108743	WAMR_108743_Ct_hele	WAMR108743	HELEW108743	helenae	inornatus−S	inornatus	WA	Banana Springs	−18.900	128.8000	KJ505431
WAMR108745	WAMR_108745_Ct_saxa	WAMR108745	SAXAW108745	saxatilis	superciliaris−W	superciliaris	WA	Mabel Downs Station	−17.283	128.1833	KJ505718
WAMR108746	WAMR_108746_Ct_saxa	WAMR108746	SAXAW108746	saxatilis	superciliaris−W	superciliaris	WA	Mabel Downs Station	−17.283	128.1833	KJ505719
WAMR108766	WAMR_108766_Ct_saxa	WAMR108766	SAXAW108766	saxatilis	superciliaris−W	superciliaris	WA	Supplejack Bore	−18.917	125.2667	KJ505720
WAMR108775	WAMR_108775_Ct_saxa	WAMR108775		saxatilis	superciliaris−W	superciliaris	WA	Mabel Downs Station	−17.283	128.1833	OQ091921
WAMR108776	WAMR_108776_Ct_saxa	WAMR108776	SAXAW108776	saxatilis	superciliaris−W	superciliaris	WA	Mabel Downs Station	−17.283	128.1833	KJ505721
WAMR108816	WAMR_108816_Ct_saxa	WAMR108816	SAXAW108816	saxatilis	superciliaris−W	superciliaris	WA	Cherralta Homestead	−21.033	116.8167	KJ505722
WAMR108911	WAMR_108911_Ct_hele	WAMR108911	HELEW108911	helenae	inornatus−S	inornatus	WA	Telfer	−21.883	122.3667	KJ505432
WAMR112190	WAMR_112190_Ct_saxa	WAMR112190	SAXAW112190	saxatilis	superciliaris−W	superciliaris	WA	Onslow	−21.741	115.1139	KJ505723
WAMR112193	WAMR_112193_Ct_saxa	WAMR112193	SAXAW112193	saxatilis	superciliaris−W	superciliaris	WA	Onslow	−21.676	115.1458	KJ505724
WAMR117679	WAMR_117679_Ct_saxa	WAMR117679	SAXAW117679	saxatilis	superciliaris−W	superciliaris	WA	Varanus Island	−20.567	115.5667	KJ505725

Voucher or Tissue Sample	Sample ID	Tissue ID	Alternative ID	Original Taxon	OTU	Putative Taxon	State or Territory	Location	Latitude	Longitude	GenBank Accession
WAMR119842	WAMR_119842_Ct_hele	WAMR119842	HELEW119842	helenae	inornatus−S	inornatus	WA	Yandicoogina	−22.717	119.0167	KJ505433
WAMR119843	WAMR_119843_Ct_hele	WAMR119843	HELEW119843	helenae	inornatus−S	inornatus	WA	Yandicoogina	−22.717	119.0167	KJ505434
WAMR120012	WAMR_120012_Ct_saxa	WAMR120012		saxatilis	inornatus−S	inornatus	WA	Hope Downs	−22.946	119.1625	KJ505726
WAMR120013	WAMR_120013_Ct_saxa	WAMR120013	SAXAW120013	saxatilis	inornatus−S	inornatus	WA	Hope Downs	−22.946	119.1625	KJ505727
WAMR121996	WAMR_121996_Ct_hele	WAMR121996	HELEW121996	helenae	inornatus−S	inornatus	WA	Weeli Wolli Spring	−22.917	119.2167	KJ505435
WAMR125009	WAMR_125009_Ct_saxa	WAMR125009	SAXAW125009	saxatilis	superciliaris−W	superciliaris	WA	Yandicoogina	−22.740	119.0525	KJ505728
WAMR125038	WAMR_125038_Ct_saxa	WAMR125038	SAXAW125038	saxatilis	superciliaris−W	superciliaris	WA	Yandicoogina	−22.740	119.0525	KJ505729
WAMR126010	WAMR_126010_Ct_rima	WAMR126010	RIMAW126010	rimacola	rimacola	rimacola	NA	Mt Septimus	−15.587	128.9961	KJ505029
WAMR126015	WAMR_126015_Ct_rima	WAMR126015	RIMAW126015	rimacola	rimacola	rimacola	WA	Septimus Mount	−15.587	128.9961	KJ505604
WAMR126017	WAMR_126017_Ct_inor	WAMR126017	INORW126017	inornatus	inornatus−N	inornatus	WA	Kimberley Research Station	−15.658	128.6864	KJ505490
WAMR126034	WAMR_126034_Ct_inor	WAMR126034	INORW126034	inornatus	inornatus−N	inornatus	WA	Septimus Mount	−15.587	128.9961	KJ505491
WAMR127782	WAMR_127782_Ct_saxa	WAMR127782		saxatilis	inornatus−S	inornatus	WA	Mount Tom Price Mine	−22.796	117.7900	KJ505730
WAMR127831	WAMR_127831_Ct_robu	WAMR127831	ROBUW127831	robustus	robustus−NW	robustus	WA	Mount Brockman	−22.289	117.2522	KJ505663
WAMR127835	WAMR_127835_Ct_robu	WAMR127835	ROBUW127835	robustus	robustus−NW	robustus	WA	Mount Brockman	−22.308	117.2575	KJ505664
WAMR129245	WAMR_129245_Ct_inor	WAMR129245	INORW129245	inornatus	superciliaris−E (S)	superciliaris	WA	Weaber Plain	−15.462	128.8192	KJ505492
WAMR129291	WAMR_129291_Ct_inor	WAMR129291	INORW129291	inornatus	superciliaris−E (S)	superciliaris	WA	Weaber Plain	−15.361	129.1300	KJ505493
WAMR129615	WAMR_129615_Ct_saxa	WAMR129615	SAXAW129615	saxatilis	superciliaris−W	superciliaris	WA	Newman	−22.917	119.0167	KJ505731
WAMR129923	WAMR_129923_Ct_hele	WAMR129923	HELEW129923	helenae	inornatus−S	inornatus	WA	West Angelas	−23.250	118.6667	KJ505436
WAMR130165	NA_ABTC61575_Ct_robu	ABTC61575		robustus	spaldingi−S	spaldingi	NSW	Smith's Lake	−32.400	152.5000	KJ505655
WAMR130166	NA_ABTC61574_Ct_robu	ABTC61574	ROBUA61574	robustus	spaldingi−S	spaldingi	NSW	Smith's Lake	−32.400	152.5000	KJ505654
WAMR130167	NA_ABTC61569_Ct_robu	ABTC61569	ROBUA61569	robustus	spaldingi−S	spaldingi	NSW	Smith's Lake	−32.400	152.5000	KJ505649
WAMR130173	NA_ABTC61571_Ct_robu	ABTC61571		robustus	spaldingi−S	spaldingi	NSW	Smith's Lake	−32.400	152.5000	KJ505651
WAMR130175	NA_ABTC61572_Ct_robu	ABTC61572		robustus	spaldingi−S	spaldingi	NSW	Smith's Lake	−32.400	152.5000	KJ505652
WAMR130176	NA_ABTC61570_Ct_robu	ABTC61570	ROBUA61570	robustus	spaldingi−S	spaldingi	NSW	Smith's Lake	−32.400	152.5000	KJ505650
WAMR130179	NA_ABTC61573_Ct_robu	ABTC61573	ROBUA61573	robustus	spaldingi−S	spaldingi	NSW	Smith's Lake	−32.400	152.5000	KJ505653
WAMR131009	WAMR_131009_Ct_hele	WAMR131009	HELEW131009	helenae	inornatus−S	inornatus	WA	Millstream−Chichester NP	−21.190	117.1722	KJ505437
WAMR131018	WAMR_131018_Ct_fall	WAMR131018	FALLW131018	inornatus	inornatus−S	inornatus	WA	Hamelin Homestead	−26.567	114.2333	KJ505010
WAMR131371	WAMR_131371_Ct_hele	WAMR131371	HELEW131371	helenae	inornatus−S	inornatus	WA	Nanutarra Roadhouse	−22.833	115.0333	KJ505438
WAMR131372	WAMR_131372_Ct_hele	WAMR131372	HELEW131372	helenae	inornatus−S	inornatus	WA	Nanutarra Roadhouse	−22.833	115.0333	KJ505439
WAMR131754	WAMR_131754_Ct_hele	WAMR131754	HELEW131754	helenae	inornatus−S	inornatus	WA	West Angelas	−22.874	118.5597	KJ505440

Voucher or Tissue Sample	Sample ID	Tissue ID	Alternative ID	Original Taxon	OTU	Putative Taxon	State or Territory	Location	Latitude	Longitude	GenBank Accession
WAMR131756	WAMR_131756_Ct_hele	WAMR131756	HELEW131756	helenae	inornatus−S	inornatus	WA	West Angelas	−22.874	118.5597	KJ505441
WAMR132516	WAMR_132516_Ct_saxa	WAMR132516	SAXAW132516	saxatilis	superciliaris−W	superciliaris	WA	Burrup Peninsula	−20.675	116.7561	KJ505732
WAMR132517	WAMR_132517_Ct_saxa	WAMR132517	SAXAW132517	saxatilis	superciliaris−W	superciliaris	WA	Burrup Peninsula	−20.675	116.7561	KJ505733
WAMR132522	WAMR_132522_Ct_saxa	WAMR132522	SAXAW132522	saxatilis	superciliaris−W	superciliaris	WA	Burrup Peninsula	−20.616	116.7850	KJ505734
WAMR132524	WAMR_132524_Ct_saxa	WAMR132524	SAXAW132524	saxatilis	superciliaris−W	superciliaris	WA	Burrup Peninsula	−20.672	116.7561	KJ505735
WAMR132682	WAMR_132682_Ct_saxa	WAMR132682	SAXAW132682	saxatilis	superciliaris−W	superciliaris	WA	Shay Gap	−20.616	120.2761	KJ505736
WAMR132686	WAMR_132686_Ct_saxa	WAMR132686		saxatilis	superciliaris−W	superciliaris	WA	Shay Gap	−20.579	120.3186	KJ505737
WAMR132690	WAMR_132690_Ct_saxa	WAMR132690	SAXAW132690	saxatilis	superciliaris−W	superciliaris	WA	Shay Gap	−20.600	120.2842	KJ505738
WAMR132723	WAMR_132723_Ct_saxa	WAMR132723	SAXAW132723	saxatilis	superciliaris−W	superciliaris	WA	Goldsworthy	−20.318	119.4233	KJ505739
WAMR135363	WAMR_135363_Ct_robu	WAMR135363	ROBUW135363	robustus	robustus−NW	robustus	WA	Mt Brockman Station	−22.311	117.2522	KJ505665
WAMR135395	WAMR_135395_Ct_hele	WAMR135395	HELEW135395	helenae	inornatus−S	inornatus	WA	Mt Brockman Station	−22.419	117.4089	KJ505442
WAMR135396	WAMR_135396_Ct_hele	WAMR135396	HELEW135396	helenae	inornatus−S	inornatus	WA	Mt Brockman Station	−22.420	117.4300	KJ505443
WAMR135400	WAMR_135400_Ct_hele	WAMR135400		helenae	inornatus−S	inornatus	WA	Mt Brockman Station	−22.400	117.4000	KJ505444
WAMR135404	WAMR_135404_Ct_saxa	WAMR135404	SAXAW135404	saxatilis	superciliaris−W	superciliaris	WA	Mt Brockman Station	−22.350	117.3500	KJ505740
WAMR135692	WAMR_135692_Ct_saxa	WAMR135692	SAXAW135692	saxatilis	superciliaris−W	superciliaris	WA	Broome	−17.983	122.3333	KJ505741
WAMR136872	WAMR_136872_Ct_burb	ABTC105740	BURBA105740	burbidgei	burbidgei	burbidgei	WA	Darcy Island	−15.330	124.3700	KJ504996
WAMR136874	WAMR_136874_Ct_burb	ABTC105741		burbidgei	burbidgei	burbidgei	WA	Darcy Island	−15.330	124.3700	KJ505876
WAMR137949	WAMR_137949_Ct_robu	WAMR137949	ROBUW137949	robustus	robustus−NW	robustus	WA	Kununurra	−15.356	129.1192	KJ505666
WAMR137950	WAMR_137950_Ct_robu	WAMR137950	ROBUW137950	robustus	robustus−NW	robustus	WA	Kununurra	−15.589	128.9833	KJ505667
WAMR137975	WAMR_137975_Ct_saxa	WAMR137975	SAXAW137975	saxatilis	superciliaris−W	superciliaris	WA	Coral Bay	−21.883	114.0167	KJ505742
WAMR139005	WAMR_139005_Ct_saxa	WAMR139005		saxatilis	superciliaris−W	superciliaris	WA	Mandora	−19.746	121.4575	KJ505743
WAMR139228	WAMR_139228_Ct_saxa	WAMR139228	SAXAW139228	saxatilis	superciliaris−W	superciliaris	WA	Meentheena Homestead	−21.266	120.4553	KJ505744
WAMR139260	WAMR_139260_Ct_saxa	WAMR139260	SAXAW139260	saxatilis	superciliaris−W	superciliaris	WA	Meentheena	−21.287	120.4594	KJ505746
WAMR139296	WAMR_139296_Ct_hele	WAMR139296	HELEW139296	helenae	inornatus−S	inornatus	WA	Meentheena	−21.245	120.3222	KJ505445
WAMR139450	WAMR_139450_Ct_saxa	WAMR139450	SAXAW139450	saxatilis	superciliaris−W	superciliaris	WA	Mount Minnie	−22.006	115.3422	KJ505747
WAMR139523	WAMR_139523_Ct_hele	WAMR139523	HELEW139523	helenae	inornatus−S	inornatus	WA	Giralia	−22.826	114.4447	KJ505446
WAMR140706	WAMR_140706_Ct_hele	WAMR140706	HELEW140706	helenae	inornatus−S	inornatus	WA	Hope Downs	−22.733	119.4086	KJ505447
WAMR140709	WAMR_140709_Ct_saxa	WAMR140709	SAXAW140709	saxatilis	superciliaris−W	superciliaris	WA	Hope Downs	−22.883	119.2528	KJ505748
WAMR140711	WAMR_140711_Ct_hele	WAMR140711	HELEW140711	helenae	inornatus−S	inornatus	WA	Hope Downs	−22.837	119.3758	KJ505448
WAMR140720	WAMR_140720_Ct_hele	WAMR140720		helenae	inornatus−S	inornatus	WA	Hope Downs	−22.821	119.3258	KJ505449

Voucher or Tissue Sample	Sample ID	Tissue ID	Alternative ID	Original Taxon	OTU	Putative Taxon	State or Territory	Location	Latitude	Longitude	GenBank Accession
WAMR141131	WAMR_141131_Ct_hele	WAMR141131	HELEW141131	helenae	inornatus−S	inornatus	WA	Leinster Downs Station	−27.965	120.3892	KJ505450
WAMR141300	WAMR_141300_Ct_hele	WAMR141300	HELEW141300	helenae	inornatus−S	inornatus	WA	Cape Preston	−21.011	116.1872	KJ505451
WAMR141301	WAMR_141301_Ct_hele	WAMR141301	HELEW141301	helenae	inornatus−S	inornatus	WA	Cape Preston	−21.066	116.1492	KJ505452
WAMR141372	WAMR_141372_Ct_robu	WAMR141372	ROBUW141372	robustus	robustus−NW	robustus	WA	Cape Preston	−21.044	116.1872	KJ505668
WAMR141379	WAMR_141379_Ct_robu	WAMR141379	ROBUW141379	robustus	robustus−NW	robustus	WA	Cape Preston	−21.102	116.1317	KJ505669
WAMR141380	WAMR_141380_Ct_saxa	WAMR141380		saxatilis	superciliaris−W	superciliaris	WA	Cape Preston	−20.905	116.2169	KJ505749
WAMR145206	WAMR_145206_Ct_saxa	WAMR145206	SAXAW145206	saxatilis	superciliaris−W	superciliaris	WA	Learmonth Airstrip	−22.243	114.0347	KJ505750
WAMR145210	WAMR_145210_Ct_saxa	WAMR145210	SAXAW145210	saxatilis	superciliaris−W	superciliaris	WA	Learmonth Airstrip	−22.243	114.0347	KJ505751
WAMR145211	WAMR_145211_Ct_saxa	WAMR145211	SAXAW145211	saxatilis	superciliaris−W	superciliaris	WA	Learmonth Airstrip	−22.243	114.0347	KJ505752
WAMR145244	WAMR_145244_Ct_hele	WAMR145244	HELEW145244	helenae	inornatus−S	inornatus	WA	Mount Tom Price Mine	−22.796	117.7900	KJ505453
WAMR145248	WAMR_145248_Ct_hele	WAMR145248	HELEW145248	helenae	inornatus−S	inornatus	WA	Mount Tom Price Mine	−22.808	117.7858	KJ505454
WAMR145254	WAMR_145254_Ct_hele	WAMR145254	HELEW145254	helenae	inornatus−S	inornatus	WA	Mount Tom Price Mine	−22.808	117.7847	KJ505455
WAMR145505	WAMR_145505_Ct_hele	WAMR145505	HELEW145505	helenae	inornatus−S	inornatus	WA	Port Hedland	−22.620	119.2500	KJ505456
WAMR145521	WAMR_145521_Ct_saxa	WAMR145521	SAXAW145521	saxatilis	superciliaris−W	superciliaris	WA	Port Hedland	−20.910	118.6800	KJ505753
WAMR145545	WAMR_145545_Ct_hele	WAMR145545		helenae	inornatus−S	inornatus	WA	Port Hedland	−21.060	118.7500	KJ505457
WAMR145567	WAMR_145567_Ct_hele	WAMR145567		helenae	inornatus−S	inornatus	WA	Port Hedland	−20.610	118.6100	KJ505458
WAMR145571	WAMR_145571_Ct_saxa	WAMR145571	SAXAW145571	saxatilis	superciliaris−W	superciliaris	WA	Port Hedland	−20.610	118.6100	KJ505754
WAMR145576	WAMR_145576_Ct_saxa	WAMR145576	SAXAW145576	saxatilis	superciliaris−W	superciliaris	WA	Port Hedland	−21.010	118.7000	KJ505755
WAMR145577	WAMR_145577_Ct_saxa	WAMR145577	SAXAW145577	saxatilis	superciliaris−W	superciliaris	WA	Port Hedland	−21.160	118.8100	KJ505756
WAMR145686	WAMR_145686_Ct_robu	WAMR145686	ROBUW145686	robustus	robustus−NW	robustus	WA	Abydos	−22.101	118.9914	KJ505670
WAMR145698	WAMR_145698_Ct_hele	WAMR145698		helenae	inornatus−S	inornatus	WA	Weeli Wolli Creek	−22.958	119.1789	KJ505459
WAMR145699	WAMR_145699_Ct_saxa	WAMR145699	SAXAW145699	saxatilis	superciliaris−W	superciliaris	WA	Weeli Wolli Creek	−22.958	119.1789	KJ505757
WAMR145756	WAMR_145756_Ct_hele	WAMR145756	HELEW145756	helenae	inornatus−S	inornatus	WA	Chichester Range	−21.940	118.9608	KJ505460
WAMR145926	WAMR_145926_Ct_hele	WAMR145926	HELEW145926	helenae	inornatus−S	inornatus	WA	Cundeelee	−30.723	123.4239	KJ505461
WAMR145934	WAMR_145934_Ct_hele	WAMR145934	HELEW145934	helenae	inornatus−S	inornatus	WA	Cundeelee	−30.723	123.4239	KJ505462
WAMR146012	WAMR_146012_Ct_robu	WAMR146012	ROBUW146012	robustus	robustus−NW	robustus	WA	Kimbolton	−16.743	124.0950	KJ505671
WAMR146353	WAMR_146353_Ct_robu	WAMR146353		robustus	robustus−NW	robustus	WA	Abydos Station	−22.101	118.9914	KJ505672
WAMR146354	WAMR_146354_Ct_robu	WAMR146354	ROBUW146354	robustus	robustus−NW	robustus	WA	Abydos Station	−22.111	118.9914	KJ505673
WAMR146355	WAMR_146355_Ct_hele	WAMR146355		helenae	inornatus−S	inornatus	WA	Abydos Station	−21.258	118.8161	KJ505463
WAMR146913	WAMR_146913_Ct_seve	WAMR146913	SEVEW146913	severus	inornatus−S	inornatus	WA	Mount Gibson	−29.585	117.2703	KJ505808

Voucher or Tissue Sample	Sample ID	Tissue ID	Alternative ID	Original Taxon	OTU	Putative Taxon	State or Territory	Location	Latitude	Longitude	GenBank Accession
WAMR151162	WAMR_151162_Ct_saxa	WAMR151162	SAXAW151162	saxatilis	superciliaris−W	superciliaris	WA	Tom Price	−22.679	117.7744	KJ505758
WAMR151164	WAMR_151164_Ct_robu	WAMR151164	ROBUW151164	robustus	robustus−NW	robustus	WA	Tom Price	−22.530	117.7381	KJ505674
WAMR151172	WAMR_151172_Ct_hele	WAMR151172	HELEW151172	helenae	inornatus−S	inornatus	WA	Tom Price	−22.620	117.7436	KJ505464
WAMR152991	WAMR_152991_Ct_seve	WAMR152991	SEVEW152991	severus	inornatus−S	inornatus	WA	Walga Rock	−27.399	117.4708	KJ505809
WAMR153812	WAMR_153812_Ct_saxa	WAMR153812	SAXAW153812	saxatilis	superciliaris−W	superciliaris	WA	Yardie Homestead Caravan	−21.894	114.0094	KJ505759
WAMR153904	WAMR_153904_Ct_saxa	WAMR153904	SAXAW153904	saxatilis	superciliaris−W	superciliaris	WA	Chichester Range	−22.017	118.9819	KJ505760
WAMR153905	WAMR_153905_Ct_saxa	WAMR153905	SAXAW153905	saxatilis	superciliaris−W	superciliaris	WA	Weeli Wolli Creek	−22.917	119.2167	KJ505761
WAMR153906	WAMR_153906_Ct_saxa	WAMR153906	SAXAW153906	saxatilis	superciliaris−W	superciliaris	WA	Weeli Wolli Creek	−22.937	119.2069	KJ505762
WAMR154016	WAMR_154016_Ct_fall	WAMR154016	FALLW154016	fallens	inornatus−S	inornatus	WA	Muchea	−31.642	115.9175	KJ505384
WAMR154248	WAMR_154248_Ct_hele	WAMR154248	HELEW154248	helenae	inornatus−S	inornatus	WA	Munjina Roadhouse	−21.988	119.0219	KJ505465
WAMR154265	WAMR_154265_Ct_saxa	WAMR154265	SAXAW154265	saxatilis	superciliaris−W	superciliaris	WA	Munjina Roadhouse	−21.913	118.9717	KJ505763
WAMR154293	WAMR_154293_Ct_hele	WAMR154293	HELEW154293	helenae	inornatus−S	inornatus	WA	Munjina Roadhouse	−22.395	118.9989	KJ505466
WAMR154303	WAMR_154303_Ct_saxa	WAMR154303	SAXAW154303	saxatilis	superciliaris−W	superciliaris	WA	Chichester Range	−22.017	118.9819	KJ505764
WAMR154304	WAMR_154304_Ct_saxa	WAMR154304	SAXAW154304	saxatilis	superciliaris−W	superciliaris	WA	Weeli Wolli Creek	−22.958	119.1789	KJ505765
WAMR154305	WAMR_154305_Ct_saxa	WAMR154305	SAXAW154305	saxatilis	superciliaris−W	superciliaris	WA	Weeli Wolli Creek	−22.937	119.2069	KJ505766
WAMR156159	WAMR_156159_Ct_seve	WAMR156159	SEVEW156159	severus	inornatus−S	inornatus	WA	Waldburg Homestead	−24.750	117.3667	KJ505037
WAMR157036	WAMR_157036_Ct_hele	WAMR157036	HELEW157036	helenae	inornatus−S	inornatus	WA	Munjina Roadhouse	−22.068	118.9953	KJ505467
WAMR157153	WAMR_157153_Ct_hele	WAMR157153	HELEW157153	helenae	inornatus−S	inornatus	WA	Roy Hill	−22.784	120.5006	KJ505468
WAMR157154	WAMR_157154_Ct_hele	WAMR157154	HELEW157154	helenae	inornatus−S	inornatus	WA	Roy Hill	−22.784	120.5006	KJ505469
WAMR157161	WAMR_157161_Ct_hele	WAMR157161	HELEW157161	helenae	inornatus−S	inornatus	WA	Roy Hill	−22.754	120.4775	KJ505470
WAMR157182	WAMR_157182_Ct_saxa	WAMR157182	SAXAW157182	saxatilis	superciliaris−W	superciliaris	WA	Yanrey	−22.250	114.5078	KJ505767
WAMR157202	WAMR_157202_Ct_saxa	WAMR157202	SAXAW157202	saxatilis	superciliaris−W	superciliaris	WA	Yanrey	−22.355	114.5250	KJ505768
WAMR157207	WAMR_157207_Ct_saxa	WAMR157207	SAXAW157207	saxatilis	superciliaris−W	superciliaris	WA	Yanrey	−22.355	114.5250	KJ505769
WAMR157342	WAMR_157342_Ct_hele	WAMR157342	HELEW157342	helenae	inornatus−S	inornatus	WA	Tanami Desert	−19.900	128.8269	KJ505471
WAMR157418	WAMR_157418_Ct_hele	WAMR157418	HELEW157418	helenae	inornatus−S	inornatus	WA	Tanami Desert	−19.589	128.8603	KJ505472
WAMR157451	WAMR_157451_Ct_hele	WAMR157451	HELEW157451	helenae	inornatus−S	inornatus	WA	Tanami Desert	−19.661	128.8833	KJ505473
WAMR157469	WAMR_157469_Ct_hele	WAMR157469	HELEW157469	helenae	inornatus−S	inornatus	WA	Tanami Desert	−19.589	128.8603	KJ505474
WAMR157529	WAMR_157529_Ct_saxa	WAMR157529	SAXAW157529	saxatilis	superciliaris−W	superciliaris	WA	Pannawonica	−21.753	116.0964	KJ505770
WAMR157532	WAMR_157532_Ct_saxa	WAMR157532	SAXAW157532	saxatilis	superciliaris−W	superciliaris	WA	Pannawonica	−21.674	115.8892	KJ505771
WAMR157567	WAMR_157567_Ct_saxa	WAMR157567	SAXAW157567	saxatilis	superciliaris−W	superciliaris	WA	Pannawonica	−21.657	115.8939	KJ505772

Voucher or Tissue Sample	Sample ID	Tissue ID	Alternative ID	Original Taxon	OTU	Putative Taxon	State or Territory	Location	Latitude	Longitude	GenBank Accession
WAMR157591	WAMR_157591_Ct_saxa	WAMR157591	SAXAW157591	saxatilis	inornatus−S	inornatus	WA	Pannawonica	−21.7220	116.1042	KJ505773
WAMR157596	WAMR_157596_Ct_saxa	WAMR157596	SAXAW157596	saxatilis	superciliaris−W	superciliaris	WA	Newman	−23.3100	119.7975	KJ505774
WAMR157629	WAMR_157629_Ct_saxa	WAMR157629	SAXAW157629	saxatilis	superciliaris−W	superciliaris	WA	Newman	−23.3100	119.7975	KJ505775
WAMR157630	WAMR_157630_Ct_hele	WAMR157630	HELEW157630	helenae	inornatus−S	inornatus	WA	Newman	−23.3100	119.7569	KJ505475
WAMR157646	WAMR_157646_Ct_hele	WAMR157646	HELEW157646	helenae	inornatus−S	inornatus	WA	Newman	−23.3120	119.7956	KJ505476
WAMR157708	WAMR_157708_Ct_hele	WAMR157708		helenae	inornatus−S	inornatus	WA	Newman	−22.9340	118.8950	KJ505477
WAMR157720	WAMR_157720_Ct_hele	WAMR157720	HELEW157720	helenae	inornatus−S	inornatus	WA	Newman	−22.9340	118.8883	KJ505478
WAMR157721	WAMR_157721_Ct_hele	WAMR157721	HELEW157721	helenae	inornatus−S	inornatus	WA	Newman	−22.9340	118.8953	KJ505479
WAMR158204	WAMR_158204_Ct_saxa	WAMR158204	SAXAW158204	saxatilis	superciliaris−W	superciliaris	WA	Roy Hill	−22.4030	119.8611	KJ505776
WAMR158273	WAMR_158273_Ct_saxa	WAMR158273	SAXAW158273	saxatilis	superciliaris−W	superciliaris	WA	Roy Hill	−22.3670	119.7147	KJ505777
WAMR158342	WAMR_158342_Ct_saxa	WAMR158342	SAXAW158342	saxatilis	superciliaris−W	superciliaris	WA	Jubilee Well	−22.6010	114.2278	KJ505778
WAMR158376	WAMR_158376_Ct_saxa	WAMR158376	SAXAW158376	saxatilis	superciliaris−W	superciliaris	WA	Giralia	−22.6440	114.4150	KJ505779
WAMR166389	WAMR_166389_Ct_hele	ABTC91779	HELEA91779	helenae	inornatus−S	inornatus	WA	Morgan Range 17.3 km ENE Blackstone	−25.9325	128.4411	KJ505925
WAMR166390	WAMR_166390_Ct_hele	ABTC91786	HELEA91786	helenae	inornatus−S	inornatus	WA	Morgan Range	−25.9386	128.3897	KJ505926
WAMR166391	WAMR_166391_Ct_hele	ABTC91631	HELEA91631	helenae	inornatus−S	inornatus	WA	0.5 km E Pungkulpirri Waterhole Walter James Ranges	−24.6542	128.7553	KJ505922
WAMR166392	WAMR_166392_Ct_hele	ABTC91638	HELEA91638	helenae	inornatus−S	inornatus	WA	Kutjuntari Rockhole	−24.8914	128.7692	KJ505923
WAMR171060	WAMR_171060_Ct_burb	ABTC105769	BURBA105769	burbidgei	burbidgei	burbidgei	WA	Augustus Island	−15.3300	124.5000	KJ505877
WAMR171061	WAMR_171061_Ct_burb	ABTC105770	BURBA105770	burbidgei	burbidgei	burbidgei	WA	Augustus Island	−15.3300	124.5000	KJ505878
WAMR171066	WAMR_171066_Ct_burb	ABTC105775	BURBA105775	burbidgei	burbidgei	burbidgei	WA	Uwins Island	−15.2500	124.8300	KJ505879
WBJ1027	NA_FALLSP021_Ct_fall	P−021 FAL	FALLSP021	fallens	inornatus−S	inornatus	WA	Leusuer Nat Park	−30.1000	115.1000	KJ505383

Table S3. Posterior parameter estimates from G-PhoCS analyses. For details, see text.

**Parameter**	**Median**	**Lower 95% HPD**	**Upper 95% HPD**
N inornatus-S	217434	206579	228783
N mastigura	215954	204605	226645
T inornatus-S vs. mastigura	746053	676316	842105
M inornatus-S --> mastigura	3.16E-07	0.000000244	3.86E-07
M mastigura --> inornatus-S	2.41E-07	1.85E-07	2.98E-07
2NM inornatus-S --> mastigura	0.14	0.11	0.16
2NM mastigura --> inornatus-S	0.1	0.08	0.13
N inornatus-S	242434	228289	253783
N superciliaris-W	86349	82237	89803
T inornatus-S vs. superciliaris-W	648684	586842	734211
M inornatus-S --> superciliaris-W	6.33E-07	5.32E-07	7.37E-07
M superciliaris-W --> inornatus-S	1.26E-07	8.42E-08	1.69E-07
2NM inornatus-S --> superciliaris-W	0.11	0.09	0.12
2NM superciliaris-W --> inornatus-S	0.06	0.04	0.08
N inornatus-S	265461	248191	285362
N superciliaris-ES	41283	38651	43914
T inornatus-S vs. superciliaris-ES	557895	490789	634211
M inornatus-S --> superciliaris-ES	5.69E-07	4.40E-07	7.05E-07
M superciliaris-ES --> inornatus-S	0.000000058	0	0.000000098
2NM inornatus-S --> superciliaris-ES	0.05	0.04	0.06
2NM superciliaris-ES --> inornatus-S	0.03	0	0.05
N inornatus-S	250164	235855	264474
N superciliaris-EN	62336	59704	65132
T inornatus-S vs. superciliaris-EN	630263	572368	701316
M inornatus-S --> superciliaris-EN	2.48E-07	1.76E-07	3.20E-07
M superciliaris-EN --> inornatus-S	6.21E-08	2.39E-08	1.01E-07
2NM inornatus-S --> superciliaris-EN	0.03	0.02	0.04
2NM superciliaris-EN --> inornatus-S	0.03	0.01	0.05
N inornatus-N	141941	134211	149507
N mastigura	232895	216118	249013
T inornatus-N vs. mastigura	710526	610526	871053
M inornatus-N --> mastigura	0.000000352	2.57E-07	4.38E-07
M mastigura --> inornatus-N	4.74E-07	3.78E-07	5.83E-07
2NM inornatus-N --> mastigura	0.16	0.13	0.2
2NM mastigura --> inornatus-N	0.13	0.11	0.16
N inornatus-N	159211	150493	167270
N superciliaris-W	87993	83717	92763
T inornatus-N vs. superciliaris-W	638158	543421	771053
M inornatus-N --> superciliaris-W	7.32E-07	0.00000061	8.62E-07
M superciliaris-W --> inornatus-N	2.94E-07	2.04E-07	3.90E-07
2NM inornatus-N --> superciliaris-W	0.13	0.11	0.15
2NM superciliaris-W --> inornatus-N	0.09	0.07	0.12
N inornatus-N	167763	157730	177796
N superciliaris-ES	43092	39803	46382

T inornatus-N vs. superciliaris-ES	586842	480263	700000
M inornatus-N --> superciliaris-ES	6.32E-07	4.64E-07	8.36E-07
M superciliaris-ES --> inornatus-N	1.17E-07	0	0.00000018
2NM inornatus-N --> superciliaris-ES	0.05	0.04	0.07
2NM superciliaris-ES --> inornatus-N	0.04	0.01	0.07
N inornatus-N	161842	153618	170230
N superciliaris-EN	65625	62829	68914
T inornatus-N vs. superciliaris-EN	660526	575000	740789
M inornatus-N --> superciliaris-EN	2.40E-07	1.66E-07	3.14E-07
M superciliaris-EN --> inornatus-N	1.41E-07	8.48E-08	2.02E-07
2NM inornatus-N --> superciliaris-EN	0.03	0.02	0.04
2NM superciliaris-EN --> inornatus-N	0.05	0.03	0.06
N inornatus-N	151809	146382	156908
N robustus-NW	78947	75493	82730
T inornatus-N vs. robustus-NW	971053	877632	1069737
M inornatus-N --> robustus-NW	2.57E-08	0	4.82E-08
M robustus-NW --> inornatus-N	0.00000004	1.69E-08	6.65E-08
2NM inornatus-N --> robustus-NW	0	0	0.01
2NM robustus-NW --> inornatus-N	0.01	0.01	0.02
N inornatus-N	155921	148191	162500
N robustus-TE	18421	14967	21217
T inornatus-N vs. robustus-TE	940789	789474	1138158
M inornatus-N --> robustus-TE	2.12E-07	5.68E-08	4.79E-07
M robustus-TE --> inornatus-N	0.000000001	0	4.22E-08
2NM inornatus-N --> robustus-TE	0.01	0	0.02
2NM robustus-TE --> inornatus-N	0	0	0.01
N lateralis	113816	107895	119079
N spaldingi-S	83717	80592	87500
T lateralis vs. spaldingi-S	980263	807895	1210526
M lateralis --> spaldingi-S	6.28E-08	3.23E-08	9.68E-08
M spaldingi-S --> lateralis	2.86E-08	0.000000003	5.77E-08
2NM lateralis --> spaldingi-S	0.01	0.01	0.02
2NM spaldingi-S --> lateralis	0.01	0	0.01
N robustus-NW	80592	74342	87171
N superciliaris-W	101974	94901	108882
T robustus-NW vs. superciliaris-W	957895	822368	1150000
M robustus-NW --> superciliaris-W	6.48E-08	1.56E-08	1.40E-07
M superciliaris-W --> robustus-NW	2.00E-10	0	1.14E-08
2NM robustus-NW --> superciliaris-W	0.01	0	0.03
2NM superciliaris-W --> robustus-NW	0	0	0
N Pilbara1	250329	224013	286513
N Pilbara2	93257	81250	134211
T Pilbara1 vs. Pilbara2	405263	180263	571053
M Pilbara1 --> Pilbara2	2.08E-05	1.64E-05	2.32E-05
M Pilbara2 --> Pilbara1	0	0	6.00E-10
2NM Pilbara1 --> Pilbara2	3.9	3.65	4.51
2NM Pilbara2 --> Pilbara1	0	0	0

N Murchison1	64145	52303	75822
N Murchison2	255921	226645	284539
T Murchison1 vs. Murchison2	581579	351316	947368
M Murchison1 --> Murchison2	0	0	4.00E-10
M Murchison2 --> Murchison1	2.70E-05	2.20E-05	3.30E-05
2NM Murchison1 --> Murchison2	0	0	0
2NM Murchison2 --> Murchison1	3.48	3.27	3.7
N inornatus-N1	237500	184046	252138
N inornatus-N2	105592	97368	113487
T inornatus-N1 vs. inornatus-N2	773684	339474	6173684
M inornatus-N1 --> inornatus-N2	1.37E-05	0.000012646	1.48E-05
M inornatus-N2 --> inornatus-N1	0	0	7.00E-10
2NM inornatus-N1 --> inornatus-N2	2.89	2.73	3.07
2NM inornatus-N2 --> inornatus-N1	0	0	0
N Western shrublands	231743	209868	249178
N Pilbara	292599	279934	307237
T Western shrublands vs. Pilbara	463158	415789	521053
M Western shrublands --> Pilbara	3.85E-07	1.67E-07	5.81E-07
M Pilbara --> Western shrublands	2.01E-06	1.60E-06	2.52E-06
2NM Western shrublands --> Pilbara	0.23	0.1	0.33
2NM Pilbara --> Western shrublands	0.93	0.79	1.08
N Western shrublands	248849	236842	260033
N inornatus-N	297039	283224	309868
T Western shrublands vs. inornatus-N	602632	543421	661842
M Western shrublands --> inornatus-N	5.89E-07	4.49E-07	7.27E-07
M inornatus-N --> Western shrublands	6.83E-07	5.70E-07	0.000000803
2NM Western shrublands --> inornatus-N	0.35	0.28	0.42
2NM inornatus-N --> Western shrublands	0.34	0.29	0.39
N inornatus-N	298849	288158	309211
N Pilbara	261678	252467	269901
T inornatus-N vs. Pilbara	631579	590789	673684
M inornatus-N --> Pilbara	2.83E-07	0.000000225	3.41E-07
M Pilbara --> inornatus-N	3.76E-07	2.90E-07	0.000000466
2NM inornatus-N --> Pilbara	0.15	0.12	0.17
2NM Pilbara --> inornatus-N	0.22	0.18	0.27
N C. brachyonyx	14803	11020	18750
N Central deserts	423520	366612	486184
T C. brachyonyx vs. Central deserts	506579	415789	600000
M C. brachyonyx --> Central deserts	0	0	0.000000149
M Central deserts --> C. brachyonyx	7.20E-06	5.06E-06	9.52E-06
2NM C. brachyonyx --> Central deserts	0	0	0.12
2NM Central deserts --> C. brachyonyx	0.21	0.19	0.24
N C. brachyonyx	24671	20395	28783
N Western shrublands	294737	276809	311842
T C. brachyonyx vs. Western shrublands	580263	463158	714474
M C. brachyonyx --> Western shrublands	2.40E-09	0	7.61E-08
M Western shrublands --> C. brachyonyx	3.11E-06	0.000002388	3.98E-06

2NM C. brachyonyx --> Western shrublands	0	0	0.04
2NM Western shrublands --> C. brachyonyx	0.15	0.14	0.17
N C. fallens	56579	52796	60197
N Pilbara	305263	288980	321546
T C. fallens vs. Pilbara	440789	390789	500000
M C. fallens --> Pilbara	0.000000129	4.63E-08	0.000000228
M Pilbara --> C. fallens	1.39E-06	0.000001129	1.70E-06
2NM C. fallens --> Pilbara	0.08	0.03	0.14
2NM Pilbara --> C. fallens	0.16	0.13	0.18
N C. fallens	65132	61184	69572
N Western shrublands	286184	270230	304112
T C. fallens vs. Western shrublands	518421	439474	597368
M C. fallens --> Western shrublands	1.97E-07	0.000000098	3.03E-07
M Western shrublands --> C. fallens	7.38E-07	5.07E-07	9.78E-07
2NM C. fallens --> Western shrublands	0.11	0.06	0.17
2NM Western shrublands --> C. fallens	0.1	0.07	0.12
N Pilbara	282566	254934	314474
N C. severus	90789	83553	97204
T Pilbara vs. C. severus	375000	303947	451316
M Pilbara --> C. severus	2.00E-10	0	1.90E-07
M C. severus --> Pilbara	2.17E-06	1.51E-06	2.77E-06
2NM Pilbara --> C. severus	0	0	0.03
2NM C. severus --> Pilbara	1.23	0.95	1.49
N Western shrublands	275493	254605	301480
N C. severus	87664	78783	97204
T Western shrublands vs. C. severus	502632	396053	618421
M Western shrublands --> C. severus	1.60E-07	0	5.62E-07
M C. severus --> Western shrublands	6.23E-07	3.25E-07	8.98E-07
2NM Western shrublands --> C. severus	0.03	0	0.09
2NM C. severus --> Western shrublands	0.34	0.21	0.49
N inornatus-N	511184	489474	530428
N inornatus-S	271382	258059	283882
T inornatus-N vs. inornatus-S	602632	572368	636842
M inornatus-N --> inornatus-S	9.42E-07	7.92E-07	1.11E-06
M inornatus-S --> inornatus-N	5.63E-07	4.72E-07	6.46E-07
2NM inornatus-N --> inornatus-S	0.51	0.45	0.58
2NM inornatus-S --> inornatus-N	0.58	0.49	0.66
N inornatus-S	541283	519901	562993
N lateralis	197862	189967	205099
T inornatus-S vs. lateralis	650000	615789	684211
M inornatus-S --> lateralis	3.80E-07	3.06E-07	4.60E-07
M lateralis --> inornatus-S	7.93E-08	1.01E-08	1.31E-07
2NM inornatus-S --> lateralis	0.15	0.12	0.18
2NM lateralis --> inornatus-S	0.09	0.01	0.14
N eutaenius	72862	67434	79276
N lateralis	241283	226316	255428
T eutaenius vs. lateralis	622368	544737	734211

M eutaenius --> lateralis	5.98E-08	0	1.09E-07
M lateralis --> eutaenius	8.86E-08	0	2.41E-07
2NM eutaenius --> lateralis	0.03	0	0.05
2NM lateralis --> eutaenius	0.01	0	0.03
N mastigura	576316	557072	595888
N superciliaris-W	153289	148191	158882
T mastigura vs. superciliaris-W	957895	894737	1051316
M mastigura --> superciliaris-W	6.78E-07	6.14E-07	7.42E-07
M superciliaris-W --> mastigura	0.00000018	1.38E-07	2.16E-07
2NM mastigura --> superciliaris-W	0.21	0.19	0.22
2NM superciliaris-W --> mastigura	0.21	0.16	0.25
N superciliaris-ES	81414	77303	85197
N mastigura	589967	568092	611678
T superciliaris-ES vs. mastigura	951316	868421	1043421
M superciliaris-ES --> mastigura	8.67E-08	5.95E-08	1.15E-07
M mastigura --> superciliaris-ES	2.73E-07	0.000000202	3.41E-07
2NM superciliaris-ES --> mastigura	0.1	0.07	0.13
2NM mastigura --> superciliaris-ES	0.04	0.03	0.05
N superciliaris-ES	80428	76645	83882
N superciliaris-W	197368	190954	203783
T superciliaris-ES vs. superciliaris-W	522368	460526	589474
M superciliaris-ES --> superciliaris-W	1.76E-07	1.13E-07	2.44E-07
M superciliaris-W --> superciliaris-ES	2.76E-07	1.38E-07	4.15E-07
2NM superciliaris-ES --> superciliaris-W	0.07	0.05	0.1
2NM superciliaris-W --> superciliaris-ES	0.04	0.02	0.07
N superciliaris-EN	138980	135197	142928
N mastigura	561513	543586	577961
T superciliaris-EN vs. mastigura	1098684	1039474	1165789
M superciliaris-EN --> mastigura	1.07E-07	8.47E-08	1.28E-07
M mastigura --> superciliaris-EN	7.48E-08	5.16E-08	1.01E-07
2NM superciliaris-EN --> mastigura	0.12	0.1	0.14
2NM mastigura --> superciliaris-EN	0.02	0.01	0.03
N superciliaris-EN	147533	142928	151645
N superciliaris-W	195395	189638	201645
T superciliaris-EN vs. superciliaris-W	736842	690789	786842
M superciliaris-EN --> superciliaris-W	1.88E-07	0.000000142	2.33E-07
M superciliaris-W --> superciliaris-EN	5.93E-08	2.84E-08	9.25E-08
2NM superciliaris-EN --> superciliaris-W	0.07	0.06	0.09
2NM superciliaris-W --> superciliaris-EN	0.02	0.01	0.03
N superciliaris-EN	161513	155428	167599
N superciliaris-ES	86184	81579	90789
T superciliaris-EN vs. superciliaris-ES	527632	490789	567105
M superciliaris-EN --> superciliaris-ES	1.99E-07	7.97E-08	3.41E-07
M superciliaris-ES --> superciliaris-EN	8.00E-10	0	5.87E-08
2NM superciliaris-EN --> superciliaris-ES	0.03	0.01	0.06
2NM superciliaris-ES --> superciliaris-EN	0	0	0.02
N C. borealis	202961	168750	234046

N robustus-NW	147862	130099	166283
T C. borealis vs. robustus-NW	339474	256579	432895
M C. borealis --> robustus-NW	1.56E-06	9.06E-07	2.27E-06
M robustus-NW --> C. borealis	2.01E-06	1.13E-06	3.10E-06
2NM C. borealis --> robustus-NW	0.46	0.29	0.63
2NM robustus-NW --> C. borealis	0.82	0.51	1.13
N spaldingi-NE	163487	158388	167928
N spaldingi-S	171217	167599	175658
T spaldingi-NE vs. spaldingi-S	918421	857895	984211
M spaldingi-NE --> spaldingi-S	5.26E-08	3.52E-08	7.26E-08
M spaldingi-S --> spaldingi-NE	2.17E-07	1.84E-07	2.51E-07
2NM spaldingi-NE --> spaldingi-S	0.02	0.01	0.02
2NM spaldingi-S --> spaldingi-NE	0.07	0.06	0.08
N spaldingi-CY	88158	82401	93586
N spaldingi-NE	170888	163980	176974
T spaldingi-CY vs. spaldingi-NE	1134211	978947	1538158
M spaldingi-CY --> spaldingi-NE	9.86E-08	6.77E-08	1.29E-07
M spaldingi-NE --> spaldingi-CY	7.21E-08	1.08E-08	1.39E-07
2NM spaldingi-CY --> spaldingi-NE	0.03	0.02	0.04
2NM spaldingi-NE --> spaldingi-CY	0.01	0	0.02
N spaldingi-NE	166118	161678	170395
N robustus-NW	196875	191941	201645
T spaldingi-NE vs. robustus-NW	1256579	1184211	1323684
M spaldingi-NE --> robustus-NW	1.55E-08	0.000000006	2.52E-08
M robustus-NW --> spaldingi-NE	6.76E-08	5.24E-08	8.47E-08
2NM spaldingi-NE --> robustus-NW	0.01	0	0.01
2NM robustus-NW --> spaldingi-NE	0.02	0.02	0.03
N spaldingi-CY	85197	80428	90296
N spaldingi-S	170559	165789	175658
T spaldingi-CY vs. spaldingi-S	961842	857895	1076316
M spaldingi-CY --> spaldingi-S	0.00000002	0	3.65E-08
M spaldingi-S --> spaldingi-CY	7.07E-08	2.15E-08	1.34E-07
2NM spaldingi-CY --> spaldingi-S	0.01	0	0.01
2NM spaldingi-S --> spaldingi-CY	0.01	0	0.02
N robustus-NW	216612	207566	226645
N robustus-TE	28947	25658	32566
T robustus-NW vs. robustus-TE	815789	728947	919737
M robustus-NW --> robustus-TE	3.08E-07	1.57E-07	4.75E-07
M robustus-TE --> robustus-NW	1.00E-10	0	2.25E-08
2NM robustus-NW --> robustus-TE	0.02	0.01	0.03
2NM robustus-TE --> robustus-NW	0	0	0.01
